# NeRD: a multichannel neural network to predict cellular response of drugs by integrating multidimensional data

**DOI:** 10.1186/s12916-022-02549-0

**Published:** 2022-10-17

**Authors:** Xiaoxiao Cheng, Chong Dai, Yuqi Wen, Xiaoqi Wang, Xiaochen Bo, Song He, Shaoliang Peng

**Affiliations:** 1 College of Computer Science and Electronic Engineering, Hunan University, Changsha, China; 2 College of Life Science and Technology, Beijing University of Chemical Technology, Beijing, China; 3 Department of Biotechnology, Beijing Institute of Health Service and Transfusion Medicine, Beijing, China; 4 The State Key Laboratory of Chemo/Biosensing and Chemometrics, Hunan University, Changsha, China

**Keywords:** Precision medicine, Drug response, Data integration, Deep learning

## Abstract

**Background:**

Considering the heterogeneity of tumors, it is a key issue in precision medicine to predict the drug response of each individual. The accumulation of various types of drug informatics and multi-omics data facilitates the development of efficient models for drug response prediction. However, the selection of high-quality data sources and the design of suitable methods remain a challenge.

**Methods:**

In this paper, we design NeRD, a multidimensional data integration model based on the PRISM drug response database, to predict the cellular response of drugs. Four feature extractors, including drug structure extractor (DSE), molecular fingerprint extractor (MFE), miRNA expression extractor (mEE), and copy number extractor (CNE), are designed for different types and dimensions of data. A fully connected network is used to fuse all features and make predictions.

**Results:**

Experimental results demonstrate the effective integration of the global and local structural features of drugs, as well as the features of cell lines from different omics data. For all metrics tested on the PRISM database, NeRD surpassed previous approaches. We also verified that NeRD has strong reliability in the prediction results of new samples. Moreover, unlike other algorithms, when the amount of training data was reduced, NeRD maintained stable performance.

**Conclusions:**

NeRD’s feature fusion provides a new idea for drug response prediction, which is of great significance for precise cancer treatment.

**Supplementary Information:**

The online version contains supplementary material available at 10.1186/s12916-022-02549-0.

## Background

Due to their heterogeneity, tumors from the same tissue origin and pathologic classification exhibit a high degree of genetic and phenotypic variation in individuals [1]. In practice, this translates to differential reactions to treatment. Therefore, to achieve precision medicine, the genetic background and medical history of patients should be considered [2]. Accurate computational prediction of cancer patients’ responses to drug treatment is essential and meaningful to the achievement of precision medication [3]. However, the lack and inaccessibility of data on cancer patients is the limitation for large-scale computational predictions of drug response. In contrast, cell line-based drug response data are abundant and readily available, providing a basis for drug response prediction. Moreover, using the drug response data of cell lines for drug response prediction is the foundation and the most important step in the realization of precision medicine [4]. Furthermore, the effective integration of various types of drug informatics and multi-omics data presents an opportunity to develop drug response prediction models [5, 6].

With the rapid development of biotechnology and the ongoing progress of sequencing technology, a large amount of multi-omics and pharmacological data has been accumulated [7, 8]. In recent years, data from several large-scale drug screening initiatives have been made available, including Genomics of Drug Sensitivity in Cancer (GDSC) [9], Cancer Cell Line Encyclopedia (CCLE) [10], and the US National Cancer Institute 60 human tumor cell line anticancer drug screen (NCI60) [11]. The GDSC database^1^ is the largest public resource for information on drug sensitivity in cancer cells and molecular markers of drug response. It currently contains nearly 75,000 items of experimental drug sensitivity data, describing the responses of 138 anti-cancer drugs in nearly 700 cancer cell lines [9]. The CCLE database^2^ is a compilation of gene expressions, chromosomal copy numbers, and massively parallel sequencing data from 947 human cancer cell lines, covering the responses of 24 drugs in 504 cancer cell lines [10]. NCI60 is an in vitro drug discovery tool developed in the late 1980s, which aims to replace the use of transplantable animal tumors in anti-cancer drug screening and test the drug responses of 52,671 drugs in 60 cancer cell lines [11]. They have helped advance the field of precision medicine. However, these studies either test the cellular response of numerous compounds to a limited number of cell lines (e.g., the NCI60 panel), or of a limited number of tumor compounds to numerous cell lines (e.g., the GDSC project). The ideal study should involve a number of drugs (most non-oncologic) screened in a large panel of genomically featured cell lines to capture the molecular diversity of human cancer [12].

To address this problem, Yu et al. reported a biotechnological method called profiling relative inhibition simultaneously in mixtures (PRISM) [13]. Jin et al. applied this method to 500 cell lines covering 21 types of solid tumors and mapped the first generation of human cancer cell metastases, which validated the reliability of the method [14]. Corsello et al. used this method to build a PRISM drug repurposing resource^3^ database, for which 4518 drugs were tested for growth-inhibitory activity in 578 human cancer cell lines, i.e., a large-scale drug screening process. They thought that this database could be used to build a drug response prediction model in cancer cell lines, thereby suggesting potentially relevant patient groups [12].

Drug response prediction is a core issue of precision medicine. Benefiting from these public datasets, researchers have developed a variety of effective computational methods to predict drug responses in cancer cell lines, thereby promoting the advancement of anti-cancer drug discovery. The rapid development of machine learning also has had a profound impact on biological and medical applications. Menden et al. first develop cancer pharmaco-omics model using multilayer perceptron (MLP). Menden et al. [15] Ridge regression [16], Lasso regression [17], random forest (RF) [18], and some Bayes-based methods [19, 20] are used to build drug response prediction models. Due to their powerful capabilities in model integration, such algorithms have been used to conduct systematic research on drug response prediction, combined with integrated strategies and multicore multitask learning techniques [21–23]. Nevertheless, because of the complexity of multi-omics data, these methods often face the problem of “small n, big p,” i.e., a feature dimension much greater than the number of samples [24]. This makes it difficult for such methods to effectively extract features from complex omics data. Some researchers [25–27] focus on feature selection, which is a major antidote to the statistical and computational problems that the high-dimensional omics input data typically entail [28], to improve prediction accuracy on classic machine learning models. Auto-HMM-LMF [29] and Dr.VAE [30] used autoencoders to solve the problem of high dimensionality of omics data, but they did not explore the characteristics of drugs. Effective fusion of multi-omics data is also one of the core issues of drug response prediction. Existing fusion categories can be summarized as early-fusion [15, 31], late-fusion [32] and intermediate-fusion [24, 33, 34]. Intermediate-fusion shows better performance in this problem. In tCNNS [33], a set of twin convolutional neural networks (CNNs) was used to combine the simplified molecular input line entry specification (SMILES) of a drug with the genome mutation data of a cell line. However, the limitations of CNNs render it unable to deal with features of different data structures and dimensions. GraphDRP [24] and DeepCDR [34] extract the drug structure information represented by a graph through a graph convolutional network (GCN). Although they made some progress in model performance, they only used a single drug feature. Furthermore, using multisource information fusion with insufficient data to train the model, to maintain good prediction accuracy poses a challenge. The scarcity of data due to the high cost of labeling remains the main problem in biomedical applications.

In response to the above problems, we propose a multichannel Neural network model to predict the cellular Response of Drugs (NeRD), using the PRISM drug response database. NeRD combines a one-dimensional CNN, stacked autoencoder, and GCN to effectively extract and integrate the global and local structure of a drug, as well as the cell line characteristics from multi-omics data. The fully connected network is then used to predict the final drug response score. Experimental results show that our method can effectively integrate multisource information and combine the features of different data structures and dimensions. NeRD outperformed seven comparison methods on all evaluation metrics on the PRISM database. Moreover, when the amount of training data was reduced, NeRD maintained stable performance and was more robust than the comparison algorithms. We summarized our contributions as follows.

- An accurate drug response prediction model NeRD is proposed. The model with a multichannel structure can effectively extract the features with different data structures and dimensions and integrate multisource information of drugs and cell lines.
- The fusion of multisource information makes the model more robust. Unlike other algorithms, when the amount of training data is reduced, NeRD maintains stable performance.
- We use a recently proposed database PRISM and prove its practicability. The database contains more drug-cell line pairs and is worthy of attention by researchers.

## Methods

### Database and data preprocessing

The data we use comes from the PRISM drug repurposing database, which contains the IC50 values, i.e., the concentration of a drug required to inhibit 50% of the cell line activity, for 1448 drugs across 480 cell lines. The lower the value the better the drug’s effect. We retrieved the SMILES feature characterizing the overall structure information of all drugs and the molecular fingerprint feature of local structure information. For cell lines, we selected the DNA copy number and miRNA expression data from multiple omics features. A total of 388 cell lines had data on both of the above omics features. The meanings of features and the reasons for selecting them are as follows.

#### Simplified molecular input line entry specification (SMILES)

An ASCII string represents the three-dimensional chemical structures of drugs. We used the RDKit toolkit [35] to transform a SMILES string to a molecular graph that reflects interactions between atoms inside drugs. Each atom was represented by a node, and the bonds between atoms were represented by edges. And each node contains five types of atom features: atom symbol, atom degree calculated by the number of bonded neighbors and hydrogen atoms, total number of hydrogen atoms, implicit value of the atom, and whether the atom is aromatic. These atom features are encoded into a 78-dimensional binary vector [24]. The RDKit functions we used and their descriptions can be found in Additional file 1: Table S1.

#### Molecular fingerprint

For 1448 drugs, we extracted chemical structure data in SDF format from the PubChem compound database [36]. Each drug was encoded into an 881-dimensional substructure vector defined in PubChem using the R package ChemmineR. Each drug is represented by a binary fingerprint that indicates the existence of a predefined chemical structure fragment. If a drug contains the corresponding chemical fingerprint, the element is 1, and otherwise it is 0.

The above two drug features were selected to extract the global and local structural features of drugs together, so as to improve the reliability of the results. In this paper, the local structure features of drugs refer to the substructure information of drug molecules represented by molecular fingerprints, because molecular fingerprints describe whether drug molecules contain certain substructures. Then the other drug feature, the molecular map, contains the information of the entire molecular structure, that is, the global structural feature of the drug.

#### Omics data

We acquired DNA copy number and miRNA expression data from the CCLE database for 338 cell lines. The DNA copy number data consist of 23,316-dimensional vectors that represent the number of occurrences of a specific DNA sequence in a haploid genome, which can reflect the characteristics of cell lines at the gene level. Studies have shown that copy number alterations are ubiquitous in cancer, and many of which are disadvantageous [37]. They are involved in the formation and progression of cancer and contribute to cancer proneness [38]. Analysis of copy number alteration data can help in cancer diagnosis and treatment by providing a better understanding of the biological and phenotypic effects of cancer [39]. Based on these studies, we have also considered this data as feature data for cancer cell lines. The miRNA expression data consist of 734-dimensional vectors. It is a kind of noncoding RNA molecule that can inhibit or degrade mRNA translation by binding to complementary target mRNA. It plays an important role in cell differentiation, proliferation, and survival [40]. Functional studies have confirmed that a causal relationship exists between abnormal miRNA regulations in many cancer cases. miRNAs, as tumor suppressors or oncogenes (oncomiRs), miRNA mimics, and molecules targeting miRNAs (antimiRs), have shown prospects in preclinical development [41].

#### Data preprocessing

To avoid the adverse effects of the different distributions of the DNA copy number and miRNA expression data on model training, we normalized them before inputting the feature extraction channels. For the drug SMILES (graph) and molecular fingerprint (binary vector), due to the particularity of the data format, we performed no processing before input. The range of values for IC50 is too large, and there are outliers. Therefore, we logarithmically processed the raw data while ensuring that the original IC50 values could be recovered. We also used a box-plot to remove outliers [42]. We took the upper quartile $Q_{3}$ and lower quartile $Q_{1}$ of all response data. Then, we got the interquartile range $IQR=Q_{3}-Q_{1}$. Finally, IC50 values less than $Q_{1}-1.5\times IQR$ and greater than $Q_{3}+1.5\times IQR$ were regarded as outliers. Specifically, the data we use contained 1448 drugs and 388 cell lines. Among them, there are 249,784 data with labels (44.46%). After removing the 15,976 outliers counted by the box-plot, we ended up using a total of 233,808 labels.

### Multichannel-based neural network

#### Overview

**Fig. 1:**
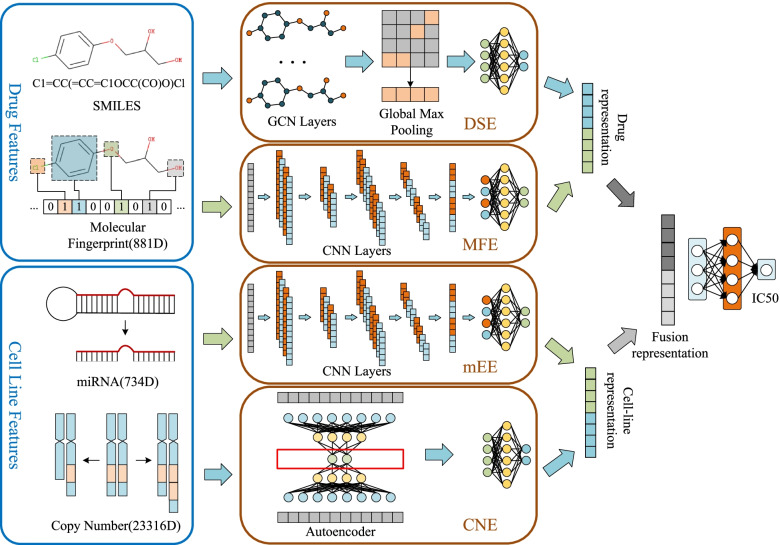
Overall model architecture. The feature extraction part of NeRD contains four feature extractors: drug structure extractor (DSE), molecular fingerprint extractor (MFE), miRNA expression extractor (mEE), and copy number extractor (CNE). After extracting the features, the drug representation and cell-line representation in the same format are combined through the fusion layer, and the IC50 value is predicted

Due to the different data structures and dimensions of drug features and cell line features, we designed different feature extraction networks for the four types of features (Fig. 1).

We use the SMILES sequence containing global structure information and the molecular fingerprint containing local structure information as drug features. The SMILES sequence describes the three-dimensional chemical structure of drugs. To extract the maximum structural information, we use SMILES in the graph form as the input of the drug structure extractor (DSE). To extract feature information from the graph, we use a method that can perform deep learning on graph data, the GCN, through which we can obtain the structural features of drug molecules. Since the data structure of a graph is different from other features and cannot be directly integrated, global maximum pooling is used to convert the feature data from a matrix to a vector, and its features are normalized to 128 dimensions through a fully connected network. Molecular fingerprints describe whether a drug has certain substructures, and can represent its local structural features. Since the data structure of the molecular fingerprint is a standardized binary vector, it can be directly used as the input of the molecular fingerprint extractor (MFE). Then, we use a one-dimensional CNN to extract the features of these substructures, and normalize them to vectors of the same dimension. The two feature vectors representing a drug are spliced to obtain its final feature representation.

We use miRNA expression data and the DNA copy number as features for cell lines. We designed a miRNA expression extractor (mEE) based on a one-dimensional CNN. We input the feature vector describing the miRNA into this channel and extracted its potential features. However, the DNA copy number cannot be directly extracted by the above neural network model due to its ultra-high dimensionality. So we designed a copy number extractor (CNE) based on a stacked autoencoder and performed nonlinear dimensionality reduction on the input data. The obtained low-dimensional feature representation was spliced with the output of the mEE to obtain the final feature representation of the cell lines.

Finally, we fuse the feature representations of drugs and cell lines and use the fully connected layers to predict the drug response in cancer cell lines. We next describe the implementation of these channels.

#### Molecular fingerprint extractor and miRNA expression extractor based on 1D CNN

For input in conventional formats, such as the molecular fingerprints of drugs and miRNA expression of cell lines, we use a one-dimensional CNN to extract their features.

We use three convolutional layers in the model, with 4, 8, and 16 convolution kernels. Each element of a convolution kernel corresponds to a weight coefficient and a bias vector, similar to a neuron of a feedforward neural network [43]. Each neuron in a convolutional layer is connected to multiple neurons in a region close to the previous layer [44]. The size of the region depends on the size of the convolution kernel, which in our model is set to 8. This area is called a receptive field in the literature, whose meaning is analogous to that of a receptive field of a visual cortex cell [45]. When a convolution kernel is working, it scans the input features regularly, conducts matrix element multiplication and summation of input features in the receptive field, and superimposes the deviation [46], so as to achieve the effect of feature extraction,

1 $$\begin{aligned} &\mathbf {Z}^{l+1}(i) =\sum \limits _{k=1}^{K_{l}}\sum \limits _{x=1}^{f}[\mathbf {Z}_{k}^{l}(s_{0}i+x)\mathbf {w}_k^{l+1}(x)]+\mathbf {b},\\ i &\ \in \{0,1, \cdots ,L_{l+1}\}\quad L_{l+1}=\frac{L_{l}+2p-f}{s_{0}}+1. \end{aligned}$$

The summation in the formula is equivalent to solving a cross-correlation. $\mathbf {b}$ is the amount of deviation, and $\mathbf {Z}^{l}$ and $\mathbf {Z}^{l+1}$ represent the input and output, respectively, of the $(l+1)$th convolutional layer. $L^{l+1}$ is the size of $\mathbf {Z}^{l+1}$. The input is assumed to be one-dimensional, and convolved in one dimensional direction only, and the two-dimensional convolution formula [44] is similar to this. $\mathbf {Z}(i)$ represents the values of the feature vector; *K* is the number of channels; and *f*, $s_{0}$, and *p* are the parameters of the convolution layer, which represent the size of the convolution kernel, the stride, and the number of padding layers [46].

After feature extraction in each convolutional layer, the output feature data are passed to the pooling layer for feature selection and information filtering. The general form of $L_{p}$ pooling is

2 $$\begin{aligned} \mathbf {A}_{k}^{l}(i)=\left[ \sum \limits _{x=1}^{f}\mathbf {A}_{k}^{l}(s_{0}i+x)^{p}\right] ^{\frac{1}{p}}, \end{aligned}$$

where *p* is a pre-specified parameter. When $p=1$, $L_{p}$ pooling takes the average value in the pooling area, which is called average pooling; when $p\rightarrow \infty$, $L_{p}$ pooling takes the maximum value in the area, i.e., max pooling [47]. Again, pooling is reduced to one-dimensional space. Our model uses the method of max pooling with a step size of 3, i.e., $p\rightarrow \infty$, $s_{0}=3$. It replaces the result of a single point in the feature vector with the feature statistics of its neighboring regions. After that, the features from the 16 channels are flattened into vectors, and the dimensions are converted to 128.

#### Copy number extractor based on stacked autoencoder

We cannot directly use conventional neural networks to extract features for DNA copy numbers with ultra-high-dimensions; we need to reduce the dimensionality in advance. Traditional methods such as PCA [48] can only reduce dimensionality in linear space and cannot perform nonlinear transformation, so we designed a stacked autoencoder [49] to predict the input by using fewer hidden nodes than the input nodes, i.e., to learn the function: $h(x)\approx x$. In other words, it must learn an approximate identity function so that the output $\hat{x}$ is approximately equal to the input *x*. For this reason, the network needs to encode as much information as possible into hidden nodes [50]. Stacked autoencoders are allowed to contain multiple hidden layers. We can learn more complex coding by adding hidden layers, but we must not make the autoencoder too powerful. If an encoder is too powerful, it just learns to map the input to an arbitrary number, and then the decoder learns its inverse mapping. Obviously, this autoencoder can reconstruct the data very well, but it cannot learn useful data representations. The autoencoder we designed contains six hidden layers, three belonging to the encoder and three to the decoder. The numbers of hidden layer neurons are 1024, 512, 256, 256, 512, and 1024. Because the traditional methods, such as the PCA method, can only reduce the dimensionality in linear space, we add nonlinear activation functions between the linear layers to enable nonlinear transformation. For the objective function during training, we use mean squared error, i.e.,

3 $$\begin{aligned} Loss=\frac{\sum \limits _{i=1}^{n} (\hat{y}_{i}-y_{i})^{2}}{n}, \end{aligned}$$

where *y* is the true value and $\hat{y}$ is the predicted value. For ultra-high-dimensional and complex features of copy numbers, our model can encode these into low-dimensional data and represent the original feature well.

#### Drug structure extractor based on GCN

A CNN is only suitable for tensor data, such as two-dimensional images or one-dimensional text sequences. However, there is much data, whose relationships are difficult to simply express with tensors. For example, to use only a one-dimensional text sequence to represent the SMILES feature of a drug will lose its structural information. Thus, we need to use another common data structure, a graph represented by vertices and edges. Specifically, the SMILES sequence of a drug is transformed to the graph $\mathbf {G}=(\mathbf {V},\mathbf {E})$ through RDKit and stored in the form of a feature matrix $\mathbf {X}$ and an adjacency matrix $\mathbf {A}$. $\mathbf {X}\in \mathbf {R}^{n\times f}$ is composed of *n* nodes in the graph, and each node is represented by an *f*-dimensional vector. $\mathbf {A}\in \mathbf {R}^{n\times n}$ represents an edge between nodes.

In order to extract the features of this kind of graph structure, we need to use a graph network. A currently popular method is to apply convolution to the graph structure, i.e., a GCN [51]. For the graph of SMILES, unlike matrix data, its convolution is difficult to define directly, so the convolution operation in the spatial domain must be transformed to matrix multiplication in the spectral domain,

4 $$\begin{aligned} \mathbf {g}_{\theta } *\mathbf {x}=\mathbf {U}\left( \mathbf {U}^{T}\mathbf {g}_{\theta } \cdot \mathbf {U}^{T}\mathbf {x}\right) , \end{aligned}$$

where *g* is the convolution kernel. The graph $\mathbf {x}$ is represented as $\mathbf {x}=(\mathbf {f}(1)\cdots \mathbf {f}(n))\in \mathbf {R}^{n}$, which is the signal at each point of the graph. $\mathbf {U}$ is the basis of the Fourier transform and the eigenvector of the Laplacian matrix. However, the cost of calculating $\mathbf {U}$ is too high, so after a series of approximate calculations, we obtain an approximate convolution formula,

5 $$\begin{aligned} \mathbf {g}_{\theta } *\mathbf {x}=\theta \left( \tilde{\mathbf {D}}^{-\frac{1}{2}} \tilde{\mathbf {A}}\tilde{\mathbf {D}}^{-\frac{1}{2}}\right) \mathbf {x}, \end{aligned}$$

where $\tilde{\mathbf {A}}$ is the graph adjacency matrix with self-loop added, which sums the node itself when summing the eigenvectors of all adjacent nodes. It is thus possible to combine information of an atom in the drug compound with its neighbors. $\tilde{\mathbf {D}}$ is the diagonal degree matrix of graph $\tilde{\mathbf {A}}$, $\tilde{\mathbf {D}}_{ii}=\sum \nolimits _{j} \tilde{\mathbf {A}}_{ij}$. The derivation process can be found in [51]. Then, after adding the nonlinear activation function $\sigma$, we can train using the graph convolutional network,

6 $$\begin{aligned} \mathbf {H}^{(l+1)}=\sigma \left( \tilde{\mathbf {D}}^{-\frac{1}{2}} \tilde{\mathbf {A}}\tilde{\mathbf {D}}^{-\frac{1}{2}}\mathbf {H}^{(l)} \mathbf {W}^{(l)}\right) , \end{aligned}$$

where $\mathbf {H}$ is the layer, and the superscript is the number of layers. Each additional graph convolution layer can aggregate the features of one more hop of neighbor nodes, thereby capturing as much neighborhood structure information as possible. $\mathbf {H}^{(0)}$ is the feature matrix $\mathbf {X}$, and $\mathbf {W}$ is the trainable parameter matrix. We use three graph convolutional layers in the model, where the dimensions of $\mathbf {W}^{(0)}$, $\mathbf {W}^{(1)}$, and $\mathbf {W}^{(2)}$ are $f\times f$, $f\times 2f$, and $f\times 4f$, respectively. Thus, the dimensions of $\mathbf {H}^{(1)}$, $\mathbf {H}^{(2)}$, and $\mathbf {H}^{(3)}$ are $n\times f$, $n\times 2f$, and $n\times 4f$, respectively. We then use global maximum pooling to convert $\mathbf {H}^{(3)}$ to a 4*f*-dimensional vector. Through the fully connected layers, the output dimension is 128.

#### Fusion layer

After the feature extraction channels, we concatenate the extracted features, fuse them through several fully connected layers, and make predictions. We add batch normalization (BN) layers between the linear layers and the nonlinear activation function to standardize the input of the activation function. This solves the problem of slow training due to inconsistent distributions of various features. Without normalization, the network needs more overhead to learn new distributions, which makes the model more complex and leads to overfitting. It also allows each layer to face the same distribution of input values, reducing the uncertainty caused by changes, and reducing the impact on subsequent layers. Each layer of the network becomes independent, which alleviates the problem of gradient disappearance in training.

After the sigmoid function, the output is mapped to (0, 1), which corresponds to the normalized value of a drug response. The steps of this method are shown as Algorithm 1.

**Algorithm 1:**
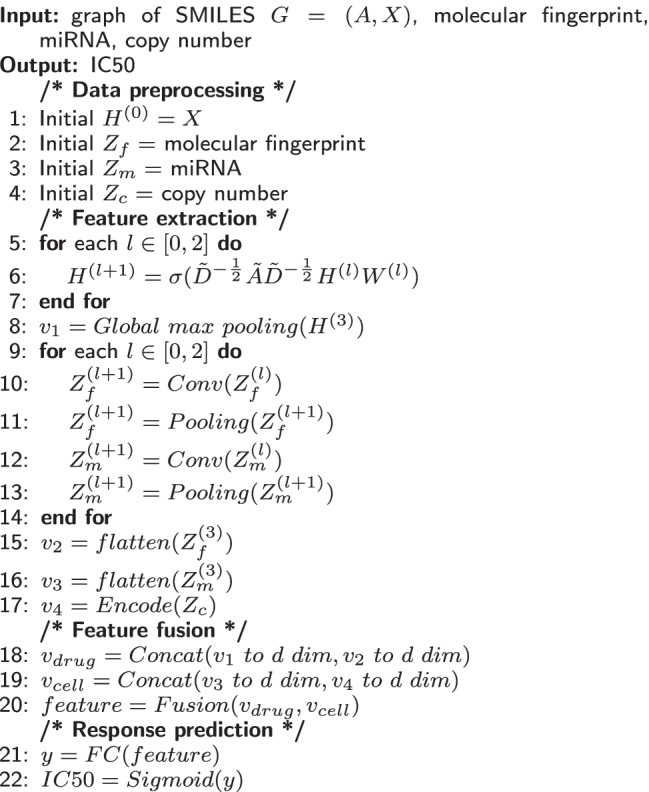
multichannel drug response prediction network

## Results

We divided drug-cell line pairs into training, validation, and test sets in an 8:1:1 ratio. The training set is used to train the models, and the model with the best result on the validation set is saved. We use the test set to test the saved model to obtain the final results. Further, we performed a five-fold cross-validation, that is, taking two pieces of data in turn as the validation set and the test set, and the remaining eight pieces as the training set. To evaluate these models, we use four classic metrics in regression: the Pearson correlation coefficient ($CC_{p}$), R squared ($R^{2}$), root mean square error (*RMSE*), and Spearman correlation coefficient ($CC_{s}$).

For NeRD, we adjusted hyperparameters such as dimensions after feature extraction, number of fusion layers, learning rate, epoch number, batch size, and dropout value according to the results of validation set. For those baseline methods, based on the principle of maintaining the original model, we also fine-tuned some hyperparameters according to the dataset we use to make the prediction results optimal. Details of hyperparameters are in Additional file 1: Table S2-S9.

After that, we designed six sets of experiments to verify the effectiveness of the proposed model from multiple perspectives.

### Performance comparison

Our baseline includes classic machine learning methods—linear regression (LR) and random forest and support vector regression (SVR, SVR-L for linear kernel-based SVR); matrix factorization-based method—SRMF [52]; deep learning methods—MLP and CNN; and advanced dual-channel methods—VAE+MLP [53], tCNNS [33], CDRScan [31], DeepCDR [34], and GraphDRP [24]. We use the same data processing and division method to obtain experimental results through different models.

**Table 1:** Performance comparison. “$\uparrow$” means the larger the value, the better; “$\downarrow$” means the smaller the value, the better. The standard deviation of the cross-validation results is calculated by the STDEVP function

Method	$CC_{p}\uparrow$	$R^{2}\uparrow$	$RMSE\downarrow$	$CC_{s}\uparrow$
LR	0.234±0.0010	0.055±0.0004	0.171±0.0002	0.237±0.0011
SVR-L	0.232±0.0013	0.047±0.0012	0.172±0.0007	0.237±0.0008
SVR	0.469±0.0034	0.213±0.0065	0.153±0.0007	0.494±0.0051
RF	0.653±0.0355	0.419±0.0405	0.130±0.0056	0.606±0.0147
MLP	0.828±0.0029	0.698±0.0048	0.104±0.0007	0.800±0.0014
CNN	0.836±0.0026	0.700±0.0044	0.097±0.0008	0.807±0.0029
SRMF	0.837±0.0022	0.701±0.0037	0.097±0.0006	0.809±0.0018
VAE+MLP	0.830±0.0036	0.688±0.0060	0.098±0.0011	0.795±0.0031
DeepCDR	0.764±0.0147	0.572±0.0223	0.115±0.0032	0.676±0.0471
CDRScan	0.834±0.0039	0.696±0.0066	0.097±0.0008	0.810±0.0038
tCNNS	0.849±0.0039	0.721±0.0067	0.093±0.0010	0.822±0.0015
GraphDRP	0.848±0.0033	0.719±0.0057	0.093±0.0010	0.821±0.0020
NeRD	0.866$\pm$0.0027	0.750$\pm$0.0048	0.088$\pm$0.0007	0.839$\pm$0.0014

It can be seen from the results in Table 1 that our proposed model performs well, with a certain degree of improvement over each baseline. Our model shows an improvement of more than 4% over the best baseline on $R^{2}$, and *RMSE* is reduced by 5% from the best baseline. $CC_{p}$ and $CC_{s}$ are also increased by more than 2%. It can also be seen from the results that the nonlinear regression method has an advantage on this problem, while the performance of the linear regression method is very poor.

### Blind test

In performance comparison experiments, it may happen that the response data of a drug to some cell lines is divided into the training set, and the response data of this drug to other cell lines is divided into the test set. However, it may be necessary to predict the response of a new drug, and we designed a blind drug test for this purpose. We randomly select 10% of the drugs and use all drug-cell line pairs associated with them as the test set. Of the remaining 90% of drugs, 80% are used for training the model, and 10% for validation. It can also be necessary to predict the response of a new cell line, for which we designed a blind cell line test. We randomly select 90% of the cell lines and use all associated drug-cell line pairs for training, and the remaining 10% for testing. The number of data instances corresponding to each data partition can be found in Additional file 1: Table S10.

**Table 2:** Cell-line blind test

Method	$CC_{p}\uparrow$	$R^{2}\uparrow$	$RMSE\downarrow$	$CC_{s}\uparrow$
LR	0.231±0.0040	0.053±0.0020	0.171±0.0004	0.233±0.0039
SVR-L	0.110±0.0663	0.045±0.0206	0.180±0.0018	0.106±0.0634
SVR	0.471±0.0168	0.218±0.0169	0.153±0.0034	0.496±0.0015
RF	0.677±0.0351	0.440±0.0506	0.141±0.0044	0.566±0.0344
MLP	0.804±0.0144	0.658±0.0244	0.110±0.0040	0.767±0.0120
CNN	0.819±0.0124	0.671±0.0208	0.101±0.0034	0.781±0.0133
SRMF	0.836±0.0091	0.699±0.0151	0.096±0.0024	0.808±0.0093
VAE+MLP	0.796±0.0108	0.623±0.0187	0.108±0.0026	0.755±0.0102
DeepCDR	0.714±0.0568	0.506±0.0771	0.123±0.0094	0.586±0.1103
CDRScan	0.815±0.0123	0.663±0.0202	0.102±0.0034	0.788±0.0107
tCNNS	0.826±0.0156	0.682±0.0264	0.099±0.0044	0.791±0.0143
GraphDRP	0.833±0.0140	0.693±0.0228	0.097±0.0039	0.801±0.0125
NeRD	0.838$\pm$0.0132	0.702$\pm$0.0229	0.096$\pm$0.0039	0.808$\pm$0.0114

**Table 3:** Drug blind test

Method	$CC_{p}\uparrow$	$R^{2}\uparrow$	$RMSE\downarrow$	$CC_{s}\uparrow$
LR	0.201±0.0244	−0.029±0.0863	0.180±0.0132	0.196±0.0351
SVR-L	0.109±0.0435	−0.208±0.2698	0.192±0.0237	0.111±0.0444
SVR	0.315±0.1793	−0.274±0.3725	0.206±0.0076	0.254±0.1311
RF	0.112±0.2737	−0.461±0.1934	0.236±0.0515	0.137±0.2424
MLP	0.261±0.0435	−0.074±0.0924	0.184±0.0135	0.202±0.0701
CNN	0.223±0.0527	0.018±0.0468	0.176±0.0113	0.169±0.0689
SRMF	0.093±0.0553	−0.006±0.0316	0.311±0.0620	0.098±0.0437
VAE+MLP	0.283±0.0242	−0.190±0.0854	0.190±0.0087	0.238±0.0347
DeepCDR	0.318±0.1403	0.010±0.1699	0.174±0.0177	0.254±0.0922
CDRScan	0.297±0.0418	0.049±0.0278	0.173±0.0098	0.229±0.0583
tCNNS	0.256±0.0261	−0.029±0.1123	0.180±0.0179	0.230±0.0335
GraphDRP	0.312±0.0926	0.067±0.0799	0.172±0.0120	0.272±0.0653
NeRD	0.370$\pm$0.0131	0.069$\pm$0.0454	0.168$\pm$0.0076	0.291$\pm$0.0426

From the results of the blind test (Tables 2 and 3), it can be seen that the results of the blind cell line test are slightly lower than those of the mixed test, and the gap between different methods is not so obvious. It is worth noting that SRMF [52], a matrix factorization-based method, has almost no performance loss in blind cell line test, compared to mixed test. As Chen et al. [54] stated, some non-deep learning methods may work better in blind testing scenarios. However, the results of the blind drug test are unsatisfactory. This is predictable, because different cell lines still have strong similarities, but different drugs are not so similar, as Liu et al. [33] says. Consequently, when a drug to be predicted does not appear in the training set, it is difficult for the models to effectively extract its features and make correct predictions. This is a common problem in existing research [24, 33, 55], and even then, NeRD still outperforms baseline models. Surprisingly, SRMF did not perform as well as in the literature [54] on drug blind test. Therefore, we compared the data from GDSC in the original study with ours, which can be found in Additional file 1: Table S11.

In addition, random partitioning of dataset may lead to uncertainty in the results on blind test. It is more convincing to use drugs or cell lines with different similarities as test sets. To do this, we grouped drugs and cell lines by their level of similarity across the dataset, respectively, and then used each group as a test set in turn.

The prediction results of blind cell line test were positively correlated with the similarity level of test sets. The higher the similarity of the test set, the more accurate the prediction result. Blind drug test did not reflect this pattern. And no matter the scenario, NeRD still outperforms other methods. Specific results can be found in Additional file 1: Table S12.

### Feature ablation experiment

We use multiple features of drugs and cell lines from different sources. We conducted a feature ablation experiment to verify the validity of the selected features. Specifically, we remove one feature of a drug or cell line, or remove one feature of each. We observe the results under these conditions and analyze the effect of each channel on the model’s performance.

**Fig. 2:**
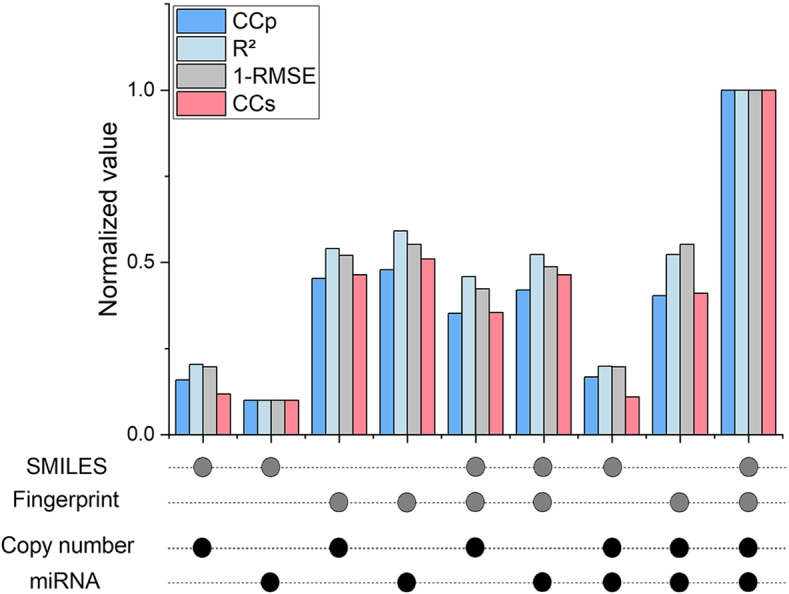
Feature ablation experiment. Due to the difference in the scope of metrics, they are normalized separately here

It can be seen from the results (Fig. 2) that when any feature is lost, each evaluation index will drop slightly. This confirms that every feature we choose is beneficial to the model. It is interesting that when the molecular fingerprint of a drug is not used, the loss of performance is the most obvious, which shows that this is indeed a good feature to represent the drug. An intuitive result is that to only use the molecular fingerprint as the feature of a drug is better than just using SMILES, but this phenomenon does not appear in the two features of the cell line.

To further investigate the influence of each channel on the prediction results, we calculate the Shapley value for the four channels, which is the sum of the marginal contributions of each channel to the outcome divided by the number of possible combinations:

7 $$\begin{aligned} \begin{aligned} \varphi _{i}(v) = \frac{\sum \nolimits _{R} [v(S)-v(S-\{i\})]}{n!}, \end{aligned} \end{aligned}$$

where *R* is the permutation of *n* channels for a total of *n*!. *S* is a permutation in *R*, *v*(*S*) is the prediction result when channel *i* is included, and $v(S-\{i\})$ is the outcome before adding channel *i*. Specifically, we calculate the Shapley values of four channels based on the evaluation indicators $CC_{p}$, $CC_{s}$, *RMSE*, and $R^{2}$ respectively, and present them in the form of percentages.

**Table 4:** Influence of channels

	Drug		Cell-line	
Channel	DSE	MFE	mEE	CNE
$CC_{p}$	22.8%	30.5%	24.4%	22.3%
$R^{2}$	21.4%	33.4%	23.8%	21.4%
*RMSE*	20.0%	32.9%	24.7%	22.4%
$CC_{s}$	22.6%	32.3%	24.3%	20.8%

As can be seen from Table 4, each feature we selected plays an integral role. Among them, the molecular fingerprint of the drug have the greatest impact on the results, exceeding 30%, which means that the molecular fingerprint of the drug may represent itself better than the molecular graph. The difference between the two features of cell lines is small, and the influence of miRNA is slightly larger, which also shows that the influencing factors of cancer are multi-faceted.

### Segment verification

To verify the effectiveness of the feature extraction and feature fusion parts of the model, we use the t-SNE algorithm to visualize the features of each stage. We analyze the effect of the model by observing the distribution of samples at different stages. We randomly select 1000 drug-cell line pairs. Before the feature is input to extraction channels, we concatenate the initial features and use t-SNE to map them to a two-dimensional space to facilitate the visualization of the sample distribution. To analyze the distinguishing ability of the feature representation, we use the value of IC50 as the label of the drug-cell line pairs to color the t-SNE graph. Similarly, the features after the four extraction channels are concatenated and mapped to a two-dimensional space, visualized, and colored. Features that have passed through the fully connected network of fusion layers are also presented.

**Fig. 3:**
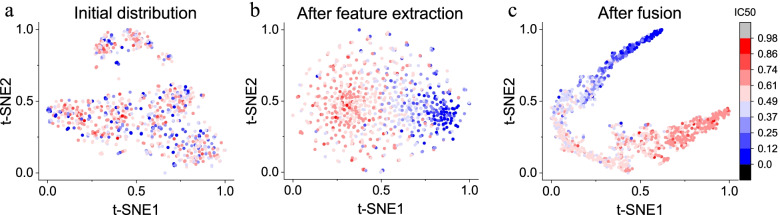
Sample distribution at different stages. **a** Initial distribution, whose features are concatenated from initial input features. **b** Sample distribution after feature extraction, whose features are concatenated from the output of four extraction channels. **c** Sample distribution after fusion layers, whose features are taken from the fully connected neural network of the fusion layers

It can be seen from Fig. 3 that before the feature extraction channels, drug-cell line pairs with different IC50 values are mixed together, with no regularity (Fig. 3a). After feature extraction, the data distribution becomes regular. Samples with high and low IC50 values are divided into the two ends of the picture, but the boundaries between other samples are not obvious (Fig. 3b). After the fusion layers, samples of middle-level IC50 are no longer mixed together, and all drug-cell line pairs are distributed in a segmented band according to the IC50 value (Fig. 3c). Data with different IC50 values are divided into different intervals.

### Data reduction experiment

Due to the scarcity of labels in actual application, the effect of many models is often much less than the experimental effect. Thus, we artificially reduce the amount of training data and observe the attenuation of the effects of each model. We randomly select a portion of each training set in five-fold cross-validation for training, and the proportion of this portion is reduced from $\frac{1}{2}$ to $\frac{1}{16}$. Then, we test NeRD and several baselines with good experimental results with different amounts of training data.

**Fig. 4:**
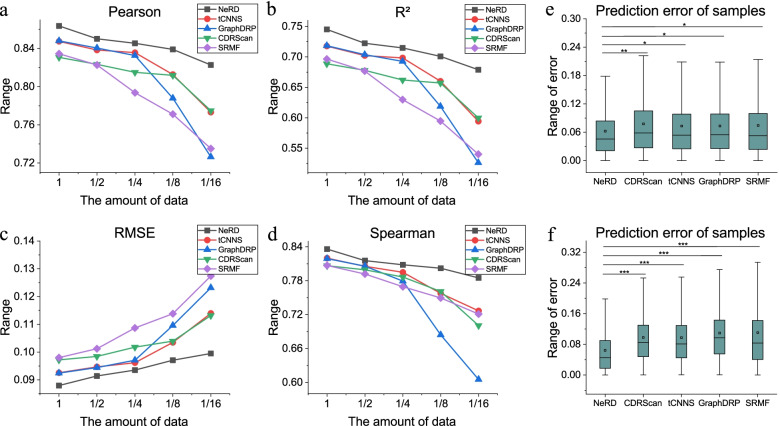
Data reduction experiment. **a** $CC_{p}$ of five methods under different data volumes. **b** $R^{2}$ of five methods under different data volumes. **c** *RMSE* of five methods under different data volumes. **d** $CC_{s}$ of five methods under different data volumes. **e** Prediction error training on 187,056 pieces (all) of data. **f** Prediction error training on 11,691 pieces (1/16) of data. We perform four sets of statistical tests on the errors of the four baselines with NeRD, calculate their *P* values, and mark them in e and f (*$p<1e-5$; **$p<1e-10$; ***$p<1e-20$)

The results of the data reduction experiment are shown in Fig. 4. It can be seen from the line charts (a–d) that the performance of each model is lost as the amount of data decreases. However, the prediction results of our model are relatively stable. Even when the amount of data is reduced to $\frac{1}{16}$ of the total, it maintains a $CC_{p}$ above 0.8 and an *RMSE* below 0.10. The performance degradation of other models is more obvious. In particular, GraphDRP, although it shows excellent performance on the original data, has results that deteriorate significantly as the amount of data continues to decrease, which may be due to the complexity of the model. To more intuitively observe the results, we draw the box plots (e, f) representing the distribution of prediction errors, from which it can be seen that when the data are sufficient (e), the prediction error of NeRD is slightly less than that of other methods. However, when data are scarce, the prediction error of the comparison methods deteriorates severely, while the results of NeRD remain stable (f).

### Pharmacogenomics analysis

**Fig. 5:**
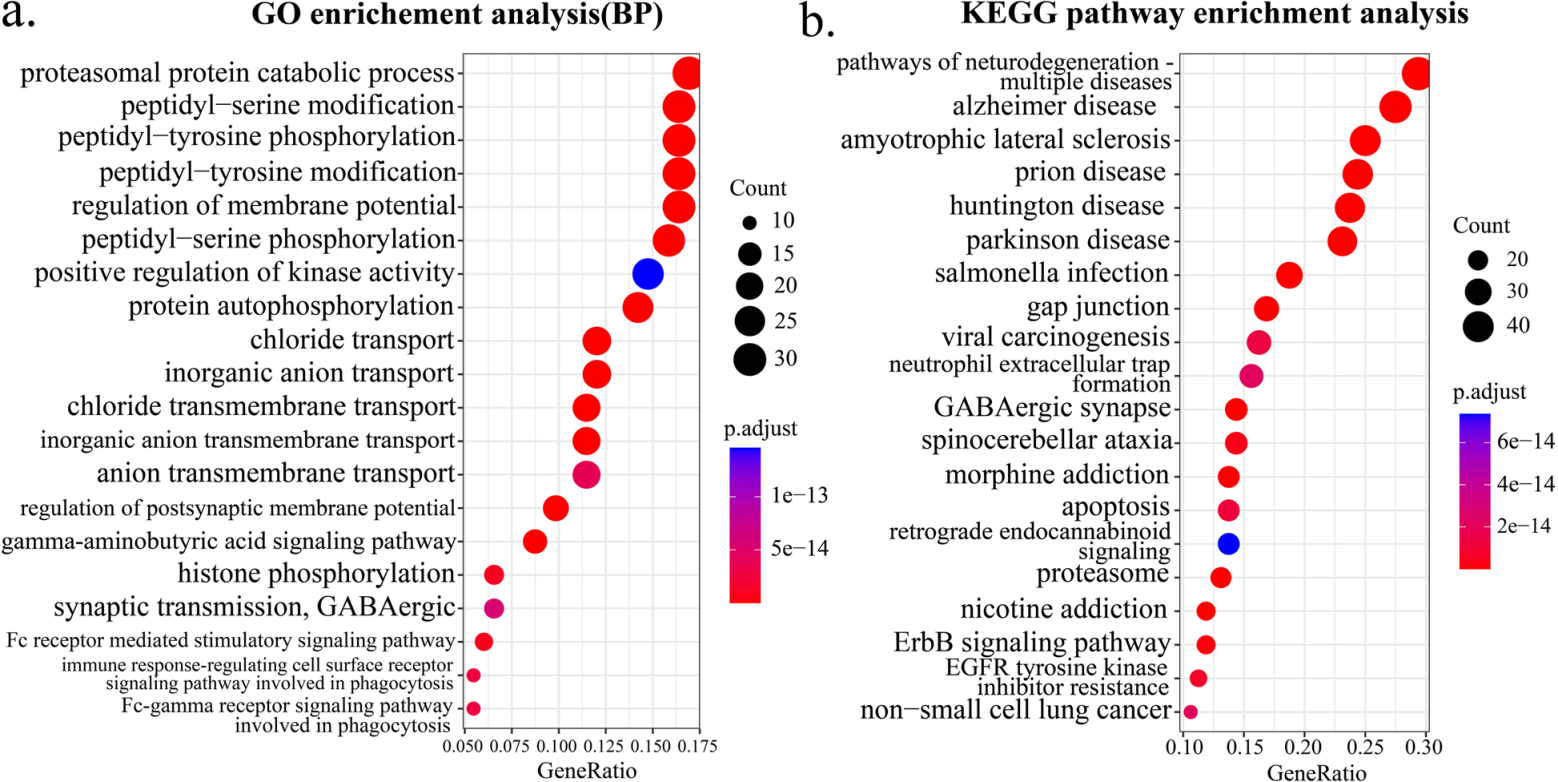
The global enrichment analysis. **a** Gene Ontology (GO) biological process enrichment analysis. **b** KEGG pathway enrichment analysis

We use the trained NeRD model to predict unknows drug-cell line pairs in PRISM database (approximately 19.5% of all pairs across 388 cancer cell lines and 1448 drugs). To verify whether the predicted results have biological and clinical significance, we sorted the newly predicted IC50 values from small to large and selected the top 1% drug-cell line pairs (altogether 2537 pairs across 383 cancer cell lines and 91 drugs) (Additional file 1: Table S13). Based on the value of IC50, we have reason to believe that these drugs have certain anticancer activity against different cancer cell lines. For this reason, we found the target genes of these 91 drugs (Additional file 1: Table S14) according to the target information of the drugs provided in PRISM database. Then, we performed two global enrichment analyses of these genes, including Gene Ontology (GO) biological process and KEGG pathway enrichment. According to the results, these genes are significantly enriched in 364 GO terms and 110 pathways (adjusted *p*-value< 0.001). The top 20 enrichment results are shown in Fig. 5. GO enrichment analysis demonstrates multiple cancer-related processes (Fig. 5a), such as ion channels and transport [56], phosphorylation of the amino acid [57, 58], and phagocytosis [59]; these processes are intimately linked to tumor progression, maintenance, and treatment. KEGG pathway enrichment analysis reveals multiple significant biological pathways (Fig. 5b), which are strongly associated with cancer. These enriched pathways including ErbB signaling pathway [60], EGFR tyrosine kinase inhibitor resistance [61], viral carcinogenesis [62], proteasome [63], and apoptosis [64], and most of them have proven to be effective therapies against cancer.

**Fig. 6:**
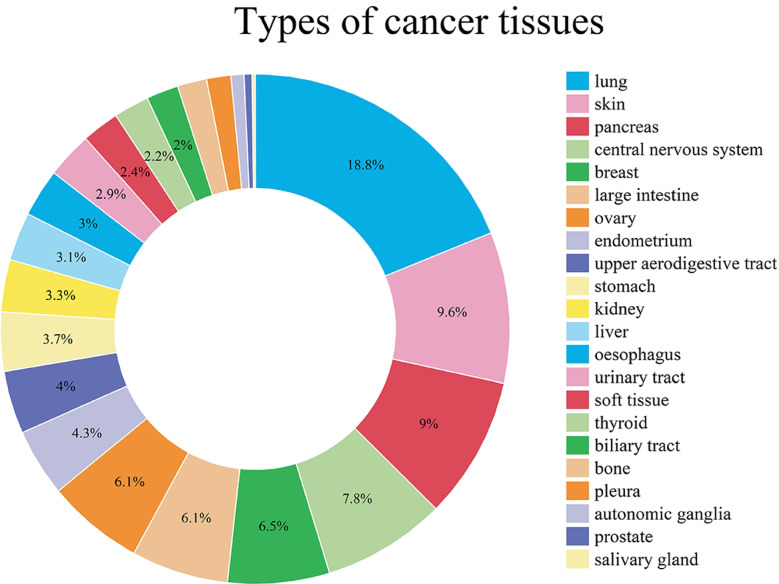
Types of cancer tissues. We classified cancer tissues in the predicted top 1% drug-cell lines pairs and selected the three most numerous tissues for analysis

**Table 5:** Case studies. Three largest cancer tissues (i.e., lung, skin, and pancreas) were screened from the predicted top 1% drug-cell line pairs, and then some drug-cell line pairs were screened from these tissues. We found that the predicted results of these drug-cell lines were consistent with those reported in the existing literature (i.e., Study)

Cell line	Cancer tissue	Drug name	Predicted IC50	Study
NCIH2122	Lung	Dasatinib	0.093358595	[65]
RERFLCAI	Lung	Dasatinib	0.124904478	[66]
NCIH1650	Lung	Bortezomib	0.046070208	[67]
NCIH322	Lung	Bortezomib	0.046246483	[11]
NCIH522	Lung	Ganetespib	0.074710398	[68]
SKMEL5	Skin	NVP-AUY922	0.038356718	[69]
UACC62	Skin	Piperazine	0.107990561	[70]
A2058	Skin	Piperazine	0.125350529	[71]
UACC62	Skin	Trametinib	0.063189984	[72]
WM1799	Skin	Trametinib	0.130639812	[73]
ASPC1	Pancreas	Dasatinib	0.120664515	[74]
PANC1005	Pancreas	Docetaxel	0.038846238	[75]
PATU8902	Pancreas	Docetaxel	0.041285745	[76]
SW1990	Pancreas	Docetaxel	0.049888730	[77]
HPAC	Pancreas	Ganetespib	0.070858083	[78]

We then also categorized the predicted top 1% of drug-cell line pairs according to the tissue that the cell line belonged to Fig. 6, selecting the three most numerous cancer tissues (i.e., lung, skin, and pancreas) for analysis. Importantly, we found that the predictive results for many of these cell line drug pairs in these tissues have been confirmed by the existing literature (Table 5). For example, in the analysis of lung cancer, dasatinib as a Src family kinases (SFKs) inhibitor can inhibit the growth and survival of non-small cell lung cancer NCI-H2122 cells [65]. In skin cancer, NVP-AUY922, a heat shock protein 90 (HSP90) inhibitor can sensitize melanoma SKMEL5 cells to it [69]. In pancreatic cancer studies, pancreatic cancer PANC1005 cells are sensitive to the tubulin polymerization inhibitor docetaxel, which is consistent with our predicted results [75]. Taken together, these case studies support that NeRD is able to effectively predict the drug sensitivity of cell lines, which can help speed up the screening of drugs and find new anti-cancer drugs in actual clinical settings.

## Discussion

We presented a multichannel neural network model, NeRD, to computationally predict cancer drug responses by integrating multi-dimensional data. We designed feature extractors DSE, MFE, mEE, and CNE to extract informative embeddings from multidimensional features of cell lines and drugs. Features extracted from each channel were converted to a uniform format, fused, and predicted. The results of five experiments show that NeRD achieves excellent performance from many aspects. First, it performs better than comparative models. Second, its generalizability was demonstrated by blind test results, and it outperformed other models when predicting new samples. Third, the results of a feature ablation experiment show that each selected feature is beneficial to the model, and that NeRD effectively fuses multiple information sources and features from different data structures and dimensions. Fourth, according to a segment verification experiment, NeRD has a strong feature extraction capability, which indirectly shows that each feature extractor designed in the model has strong utility. Fifth, NeRD has high robustness, as illustrated by a data reduction experiment. Sixth, the result of using trained NeRD for drug sensitivity prediction have biological and clinical significance. Despite NeRD having strong predictive power, the model was built on in vitro data. Challenges remain in its application. Recent studies have shown that using clinical data from some patients can better help achieve precision oncology [27, 79]. These challenges can be addressed in our future studies.

## Conclusion

In summary, we think that NeRD, as a highly extensible framework, can effectively fuse multidimensional features of cell lines and drugs to accurately predict the drug response of cell lines. Furthermore, this model can be widely applicable to integrate other omics data, thus benefiting clinical cancer therapy and future research on drug response prediction. Thus, it will provide a more diverse view of clinical cancer therapy.

## Supplementary information

**Additional file 1: Table S1.** RDKit functions and their descriptions. **Table S2.** Hyperparameters for NeRD. The adjustment of hyperparameters often has an important impact on the specific data set. **Table S3.** Hyperparameters for DeepCDR, CDRScan, tCNNS, and GraphDRP. These models are all dual-channel or quasi-dual-channel, so the same method is used to adjust the hyperparameters. **Table S4.** Hyperparameters for RF. The parameters of the RF framework are few, and the parameter selection is generally to adjust the value of N\_estimators, i.e., the number of decision trees. **Table S5.** Hyperparameters for SVR. Gamma is the coefficient of kernel functions, only valid for `rbf', `poly', and `sigmod'. The parameter Degree only works for `kernel=poly'. C represents the penalty coefficient of the error term. The larger C is, the greater the degree of penalty for wrongly classified samples. **Table S6.** Hyperparameters for CNN. What we use here is the one-dimensional convolution function provided by pytorch. **Table S7.** Hyperparameters for MLP. The number of neurons in each layer is also fine-tuned according to the number of hidden layers. **Table S8.** Hyperparameters for SRMF. SRMF is a method based on matrix factorization, and its hyperparameters mainly include the dimension of the feature space and the regularization parameters. **Table S9.** Hyperparameters for VAE+MLP. The number of neurons in each layer is also fine-tuned according to the number of hidden layers. **Table S10.** Number of data instances corresponds to each data partition in the blind test. **Table S11.** Dataset comparison. **Table S12.** Blind test dividing data by similarity. Set1-Set5 are test sets with increasing similarity. Set1 has the lowest similarity and Set5 has the highest similarity. The values are the Pearson correlation coefficients. **Table S13.** Predicted results for the top 1\% of drug-cell lines. We used the trained NERD model to predict drug cell line pairs without IC50 data in the PRISM database, sorted from small to large according to the predicted IC50 value, and then screened the top 1\% of drug-cell line pairs (altogether 2537 pairs across 383 cancer cell lines and 91 drugs). **Table S14.** The drug target gene list. Based on the list of drugs obtained from the top 1\% of predicted drug-cell line pairs, we found the target genes for these drugs from the PRISM database.

## Data Availability

The data underlying this article are available in the article and in its online supplementary material. The source code and processed data are available at https://github.com/Shaw66/NeRD. The raw data we used were from PRISM Repurposing, CCLE, and PubChem, which are all publicly available. PRISM Repurposing data can be downloaded from Corsello et al. [12]. CCLE data can be downloaded from Barretina et al. [10]. PubChem data can be downloaded from Kim et al. [36]. All relevant data are available from the authors.

## Abbreviations

BN: Batch normalization
CCLE: Cancer cell line encyclopedia
*CC*_*p*_: Pearson correlation coefficient
*CC*_*s*_: Spearman correlation coefficient
CNE: Copy number extractor
CNN: Convolutional neural network
DSE: Drug structure extractor
GCN: Graph convolutional network
GDSC: Genomics of drug sensitivity in cancer
GO: Gene ontology
LR: Linear regression
mEE: miRNA expression extractor
MFE: Molecular fingerprint extractor
MLP: Multilayer perceptron
NCI60: National cancer institute 60
PRISM: Profiling relative inhibition simultaneously in mixture
*R*^*2*^: R squared
RF: Random forest
*RMSE*: Root mean square error
SMILES: Simplified molecular input line entry specification
SVR: Support vector regression
SVR-L: linear kernel-based SVR

## Footnotes

1 https://www.cancerrxgene.org/

2 https://sites.broadinstitute.org/ccle/

3 https://depmap.org/repurposing/
