# Supplementary material for: NeRD: a multichannel neural network to predict cellular response of drugs by integrating multidimensional data
Source: BMC Med. 2022 Oct 17;20:368. doi: 10.1186/s12916-022-02549-0 (PMC9575288; doi:10.1186/s12916-022-02549-0)
Supplement: Supplementary file 1 — Additional file 1: Table S1. RDKit functions and their descriptions. Table S2. Hyperparameters for NeRD. The adjustment of hyperparameters often has an important impact on the specific data set. Table S3. Hyperparameters for DeepCDR, CDRScan, tCNNS, and GraphDRP. These models are all dual-channel or quasi-dual-channel, so the same method is used to adjust the hyperparameters. Table S4. Hyperparameters for RF. The parameters of the RF framework are few, and the parameter selection is generally to adjust the value of N\_estimators, i.e., the number of decision trees. Table S5. Hyperparameters for SVR. Gamma is the coefficient of kernel functions, only valid for `rbf', `poly', and `sigmod'. The parameter Degree only works for `kernel=poly'. C represents the penalty coefficient of the error term. The larger C is, the greater the degree of penalty for wrongly classified samples. Table S6. Hyperparameters for CNN. What we use here is the one-dimensional convolution function provided by pytorch. Table S7. Hyperparameters for MLP. The number of neurons in each layer is also fine-tuned according to the number of hidden layers. Table S8. Hyperparameters for SRMF. SRMF is a method based on matrix factorization, and its hyperparameters mainly include the dimension of the feature space and the regularization parameters. Table S9. Hyperparameters for VAE+MLP. The number of neurons in each layer is also fine-tuned according to the number of hidden layers. Table S10. Number of data instances corresponds to each data partition in the blind test. Table S11. Dataset comparison. Table S12. Blind test dividing data by similarity. Set1-Set5 are test sets with increasing similarity. Set1 has the lowest similarity and Set5 has the highest similarity. The values are the Pearson correlation coefficients. Table S13. Predicted results for the top 1\% of drug-cell lines. We used the trained NERD model to predict drug cell line pairs without IC50 data in the PRISM database, sorted from small to [file 12916_2022_2549_MOESM1_ESM.pdf]

NeRD: a multichannel neural network to predict cellular response of drugs by integrating multidimensional data

Xiaoxiao Cheng[1,†] , Chong Dai [2,†] , Yuqi Wen[3] , Xiaoqi Wang[1] , Xiaochen Bo[3,*] , Song He [3,*] and Shaoliang Peng[1,4,*]

[1]College of Computer Science and Electronic Engineering, Hunan University, Changsha, 410082, China,

[2]College of Life Science and Technology, Beijing University of Chemical Technology, Beijing, 100029, China,

[3]Department of Biotechnology, Beijing Institute of Health Service and Transfusion Medicine, Beijing, 100850, China,

[4]The State Key Laboratory of Chemo/Biosensing and Chemometrics, Hunan University, Changsha, 410082, China.

Table S1. RDKit functions and their descriptions.

Functions	Descriptions
MolFromSmiles()	Get a molecule object from SMILES.
GetBonds()	Get all the bonds in the molecule object.
GetSymbol()	Get the symbol of an atom.
GetDegree()	Get the degree of an atom.
GetTotalNumHs()	Get the total number of hydrogens.
GetImplicitValence()	Get the implicit value of an atom.
GetIsAromatic()	Get whether an atom is aromatic.

Table S2. Hyperparameters for NeRD. The adjustment of hyperparameters often has an important impact on the specific data set.

Hyperparameter	Tuned values
Dimensions after feature extraction	32, 64, **128**, 256
Dropout value	0.2, 0.3, **0.5**, 1
Number of fusion layers	1, 2, **3**, 4
Learning rate	1×10^{-2}, 1×10^{-3}, $\mathbf{1 \times 10^{-4}}$, 1×10^{-5}
Batch size	128, 256, 512, **1024**, 2048
Epoch number	100, 200, **300**, 400, 500

Note: Bold numbers represent optimal parameters.

Table S3. Hyperparameters for DeepCDR, CDRScan, tCNNS, and GraphDRP. These models are all dual-channel or quasi-dual-channel, so the same method is used to adjust the hyperparameters.

Hyperparameter	Tuned values
Dropout value	0.1, 0.2, 0.3, 0.5, 1
Number of fusion layers	1, 2, 3, 4
Learning rate	1×10^{-2}, 1×10^{-3}, 1×10^{-4}, 1×10^{-5}
Batch size	128, 256, 512, 1024, 2048
Epoch number	100, 200, 300, 400, 500

Table S4. Hyperparameters for RF. The parameters of the RF framework are few, and the parameter selection is generally to adjust the value of N_estimators, i.e., the number of decision trees.

Hyperparameter	Tuned values
N_estimators	10, 100, 1000, 10000
Max_features	log_2N, $\sqrt{N}$, auto

Table S5. Hyperparameters for SVR. Gamma is the coefficient of kernel functions, only valid for 'rbf', 'poly', and 'sigmod'. The parameter Degree only works for 'kernel=poly'. C represents the

penalty coefficient of the error term. The larger C is, the greater the degree of penalty for wrongly classified samples.

Hyperparameter	Tuned values
Kernel	'linear', 'poly', 'rbf', 'sigmoid'
C	0.1, 1, 10, 100
Gamma	'auto', 'scale'
Degree	1, 2, 3

Table S6. Hyperparameters for CNN. What we use here is the one-dimensional convolution function provided by pytorch.

Hyperparameter	Tuned values
Kernel size	4, 8, 16, 32
Stride	1, 2, 3, 4
Out_channels(including 3 convolutional layers)	{4, 8, 16}, {8, 16, 32}, {16, 32, 64}
Learning rate	1×10^{-2}, 1×10^{-3}, 1×10^{-4}, 1×10^{-5}
Batch size	128, 256, 512, 1024, 2048
Epoch number	100, 200, 300, 400, 500

Table S7. Hyperparameters for MLP. The number of neurons in each layer is also fine-tuned according to the number of hidden layers.

Hyperparameter	Tuned values
Number of hidden layers	1, 2, 3, 4
Dropout value	0.2, 0.3, 0.5, 1
Learning rate	1×10^{-2}, 1×10^{-3}, 1×10^{-4}, 1×10^{-5}
Batch size	128, 256, 512, 1024, 2048
Epoch number	100, 200, 300, 400, 500

Table S8. Hyperparameters for SRMF. SRMF is a method based on matrix factorization, and its hyperparameters mainly include the dimension of the feature space and the regularization parameters.

Hyperparameter	Tuned values
Dimensionality of the feature space K	10~50
Regularization parameter λ_l	2^-3, 2^-2, 2^-1, 2^0, 2^1, 2^2
Regularization parameter λ_c	2^-5, 2^-4, 2^-3, 2^-2, 2^-1, 2^0, 2^1, 0
Regularization parameter λ_d	2^-5, 2^-4, 2^-3, 2^-2, 2^-1, 2^0, 2^1, 0
maximum number of iterations	20, 50, 100, 200, 300

Table S9. Hyperparameters for VAE+MLP. The number of neurons in each layer is also fine-tuned according to the number of hidden layers.

Hyperparameter	Tuned values
Number of hidden layers in VAE	2, 4, 6, 8
Number of hidden layers in MLP	1, 2, 3, 4
Dropout value	0.2, 0.3, 0.5, 1
Learning rate	1×10^{-2}, 1×10^{-3}, 1×10^{-4}, 1×10^{-5}
Batch size	128, 256, 512, 1024, 2048
Epoch number	100, 200, 300, 400, 500

Table S10. Number of data instances corresponds to each data partition in the blind test.

	Fold	Cell line blind test			Drug blind test		
		Training set	Validation set	Test set	Training set	Validation set	Test set
Random division	1	187188	23345	23275	195152	18973	19683
	2	184025	25841	23942	196460	19181	18167
	3	186909	24450	22449	184921	24430	24457
	4	188686	24632	20490	180081	28311	25416
	5	188949	22398	22461	178622	26498	28688
Division by similarity	1	189591	23093	21124	193714	22750	17344
	2	187149	24559	22100	188684	24911	20213
	3	188542	23104	22162	188396	22788	22624
	4	186832	23628	23348	183790	25825	24193
	5	183865	26038	23905	180966	26695	26147

Table S11. Dataset comparison. The dataset used in this paper contains 1448 drugs and 388 cell lines, compared to 135 drugs and 652 cell lines in the original study. The huge drug quantity gap may be one of the reasons for the performance loss. In addition, for the SRMF model, the similarity matrix is the key factor of the method. The overall similarity of the drugs used in this paper is lower, which may be another important factor in the loss of performance. Besides, the drug response matrix we used was sparser (44.5%) compared to the data in the original study (96.9%). This will increase the difficulty of the prediction of the SRMF model, resulting in a loss of performance.

	PRISM (NeRD)	GDSC (SRMF)
Number of drugs	1448	135
Number of cell lines	388	652
Mean similarity of drugs	0.3710	0.4654
Mean similarity of cell lines	0.8482	0.8529
Density of drug response matrix	44.5%	96.9%

Table S12. Blind test dividing data by similarity. Set1-Set5 are test sets with increasing similarity. Set1 has the lowest similarity and Set5 has the highest similarity. The values are the Pearson correlation coefficients.

	Blind cell line test					Blind drug test				
	**Set1**	**Set2**	**Set3**	**Set4**	**Set5**	**Set1**	**Set2**	**Set3**	**Set4**	**Set5**
LR	0.223	0.230	0.234	0.235	0.236	0.013	0.168	0.130	0.223	0.211
SVR-L	0.053	0.094	0.122	0.148	0.173	0.064	0.179	0.106	0.136	0.092
SVR	0.446	0.460	0.458	0.492	0.497	-0.103	0.236	0.032	0.261	0.158
RF	0.680	0.685	0.729	0.683	0.751	-0.106	0.161	0.082	0.266	0.067
MLP	0.766	0.785	0.823	0.833	0.824	-0.152	0.139	0.138	0.245	0.236
CNN	0.747	0.788	0.816	0.824	0.829	0.004	0.287	0.165	0.299	0.106
SRMF	0.812	**0.831**	0.824	0.844	0.843	0.084	0.092	0.074	0.161	0.120
VAE+MLP	0.753	0.773	0.803	0.806	0.812	0.064	0.164	0.064	0.010	0.093
DeepCDR	0.725	0.750	0.754	0.789	0.772	0.201	0.220	0.049	0.165	0.159
CDRScan	0.784	0.791	0.808	0.823	0.832	0.001	0.303	0.163	0.298	0.118
tCNNS	0.793	0.791	0.817	0.833	0.830	0.002	0.294	0.168	0.216	0.062
GraphDRP	0.801	0.808	0.828	0.838	0.845	0.174	0.195	**0.238**	0.169	0.189
NeRD	**0.814**	0.823	**0.834**	**0.850**	**0.862**	**0.181**	**0.317**	0.217	**0.390**	**0.268**

Table S13. Predicted results for the top 1% of drug-cell lines. We used the trained NERD model to predict drug cell line pairs without IC50 data in the PRISM database, sorted from small to large according to the predicted IC50 value, and then screened the top 1% of drug-cell line pairs (altogether 2537 pairs across 383 cancer cell lines and 91 drugs).

DepMap ID	CCLE Name	Drug name	PubChem ID	Predict IC_{50}
ACH-000946	HEC265_ENDOMETRIUM	colchicine	6167	0.027812649
ACH-000756	GI1_CENTRAL_NERVOUS_SYSTEM	colchicine	6167	0.028502241
ACH-001318	PLCPRF5_LIVER	colchicine	6167	0.028582329
ACH-000885	TOV21G_OVARY	docetaxel	148124	0.030135668
ACH-000750	LOXIMVI_SKIN	docetaxel	148124	0.031132214
ACH-000532	SNU61_LARGE_INTESTINE	colchicine	6167	0.031229235
ACH-000364	U2OS_BONE	colchicine	6167	0.03145944
ACH-000846	FADU_UPPER_AERODIGESTIVE_TRACT	docetaxel	148124	0.031794277
ACH-000221	SNU398_LIVER	docetaxel	148124	0.03207263
ACH-000897	FTC238_THYROID	colchicine	6167	0.032312248
ACH-000756	GI1_CENTRAL_NERVOUS_SYSTEM	filanesib	44224257	0.032343896
ACH-000023	PATU8988T_PANCREAS	JNJ-26481585	11538455	0.032353394
ACH-000885	TOV21G_OVARY	filanesib	44224257	0.032439379
ACH-000123	COV434_OVARY	docetaxel	148124	0.032635068
ACH-000832	CAL27_UPPER_AERODIGESTIVE_TRACT	JNJ-26481585	11538455	0.033168092
ACH-000091	OV56_OVARY	docetaxel	148124	0.033323513
ACH-000048	TOV112D_OVARY	alvespimycin	5288674	0.033880365
ACH-000619	PECAPJ15_UPPER_AERODIGESTIVE_TRACT	filanesib	44224257	0.033883909
ACH-000605	TE6_OESOPHAGUS	alvespimycin	5288674	0.034012375
ACH-000361	SKHEP1_LIVER	filanesib	44224257	0.034098738

ACH-000846	FADU_UPPER_AERODIGESTIVE_TRACT	filanesib	44224257	0.034153807
ACH-000670	SBC5_LUNG	NVP-AUY922	135539077	0.034210904
ACH-000351	MKN1_STOMACH	docetaxel	148124	0.034577822
ACH-000169	RD_SOFT_TISSUE	NSC-319726	5921672	0.034719702
ACH-000280	SNU840_OVARY	docetaxel	148124	0.034728285
ACH-000849	MDAMB468_BREAST	docetaxel	148124	0.034861892
ACH-000221	SNU398_LIVER	filanesib	44224257	0.03493805
ACH-000091	OV56_OVARY	NVP-AUY922	135539077	0.034965993
ACH-000832	CAL27_UPPER_AERODIGESTIVE_TRACT	alvespimycin	5288674	0.035035707
ACH-000228	BICR31_UPPER_AERODIGESTIVE_TRACT	docetaxel	148124	0.035210738
ACH-000845	NCIH1373_LUNG	alvespimycin	5288674	0.035248591
ACH-000670	SBC5_LUNG	ganetespib	135564985	0.035254725
ACH-000756	GI1_CENTRAL_NERVOUS_SYSTEM	alvespimycin	5288674	0.035321247
ACH-000090	PC3_PROSTATE	docetaxel	148124	0.035334522
ACH-000885	TOV21G_OVARY	paclitaxel	36314	0.035348577
ACH-000091	OV56_OVARY	filanesib	44224257	0.035683645
ACH-000749	DMS273_LUNG	docetaxel	148124	0.035688902
ACH-000946	HEC265_ENDOMETRIUM	LY2606368	46700756	0.035747895
ACH-000853	NCIH661_LUNG	JNJ-26481585	11538455	0.035851104
ACH-000890	SW1271_LUNG	filanesib	44224257	0.035932698
ACH-000994	HEC59_ENDOMETRIUM	filanesib	44224257	0.036010954
ACH-000994	HEC59_ENDOMETRIUM	alvespimycin	5288674	0.036046648
ACH-000911	NUGC3_STOMACH	NVP-AUY922	135539077	0.036245701
ACH-000231	KALS1_CENTRAL_NERVOUS_SYSTEM	filanesib	44224257	0.036387281
ACH-000542	HEYA8_OVARY	cabazitaxel	129009963	0.03641196
ACH-000273	SF539_CENTRAL_NERVOUS_SYSTEM	alvespimycin	5288674	0.036424857
ACH-000929	NCIH2110_LUNG	FK-866	6914657	0.036439304
ACH-000351	MKN1_STOMACH	NVP-AUY922	135539077	0.036499681
ACH-000890	SW1271_LUNG	docetaxel	148124	0.036505909
ACH-000994	HEC59_ENDOMETRIUM	docetaxel	148124	0.036587127
ACH-000444	LU99_LUNG	filanesib	44224257	0.036600228
ACH-000811	SKOV3_OVARY	docetaxel	148124	0.036674492
ACH-000885	TOV21G_OVARY	ispinesib	6851740	0.036677704
ACH-000280	SNU840_OVARY	filanesib	44224257	0.036686219
ACH-000954	HEC1A_ENDOMETRIUM	docetaxel	148124	0.036694161
ACH-000996	HEC251_ENDOMETRIUM	docetaxel	148124	0.036772199
ACH-000863	DBTRG05MG_CENTRAL_NERVOUS_SYSTEM	docetaxel	148124	0.036801828
ACH-000943	RKO_LARGE_INTESTINE	filanesib	44224257	0.036873729
ACH-000749	DMS273_LUNG	filanesib	44224257	0.036959474
ACH-000632	HS944T_SKIN	NVP-AUY922	135539077	0.036979065
ACH-000142	CAL29_URINARY_TRACT	NVP-AUY922	135539077	0.037049626
ACH-000211	DAOY_CENTRAL_NERVOUS_SYSTEM	FK-866	6914657	0.037078553
ACH-000311	NCIH2122_LUNG	delanzomib	24800541	0.037126063

ACH-000013	ONCODG1_OVARY	NVP-AUY922	135539077	0.037153566
ACH-000909	JHUEM2_ENDOMETRIUM	filanesib	44224257	0.037209309
ACH-000846	FADU_UPPER_AERODIGESTIVE_TRACT	paclitaxel	36314	0.0372512
ACH-000696	OVCAR8_OVARY	filanesib	44224257	0.037300905
ACH-000288	BT549_BREAST	alvespimycin	5288674	0.037371532
ACH-000955	SNU407_LARGE_INTESTINE	docetaxel	148124	0.037441603
ACH-000901	HCC1359_LUNG	filanesib	44224257	0.037447349
ACH-000311	NCIH2122_LUNG	FK-866	6914657	0.037499889
ACH-000472	HSC2_UPPER_AERODIGESTIVE_TRACT	docetaxel	148124	0.037503385
ACH-000221	SNU398_LIVER	alvespimycin	5288674	0.037505158
ACH-000488	TE11_OESOPHAGUS	NVP-AUY922	135539077	0.037507569
ACH-000945	NCIH650_LUNG	filanesib	44224257	0.037596928
ACH-001075	NCIH292_LUNG	docetaxel	148124	0.037610171
ACH-000231	KALS1_CENTRAL_NERVOUS_SYSTEM	docetaxel	148124	0.03781132
ACH-000845	NCIH1373_LUNG	docetaxel	148124	0.037829613
ACH-000142	CAL29_URINARY_TRACT	filanesib	44224257	0.037905251
ACH-000407	SNU685_ENDOMETRIUM	filanesib	44224257	0.037930569
ACH-000374	HCC1143_BREAST	colchicine	6167	0.037940052
ACH-000674	NUGC4_STOMACH	NVP-AUY922	135539077	0.037996141
ACH-000102	GMS10_CENTRAL_NERVOUS_SYSTEM	colchicine	6167	0.038011207
ACH-000954	HEC1A_ENDOMETRIUM	filanesib	44224257	0.038015196
ACH-000231	KALS1_CENTRAL_NERVOUS_SYSTEM	NVP-AUY922	135539077	0.038044504
ACH-000846	FADU_UPPER_AERODIGESTIVE_TRACT	NVP-AUY922	135539077	0.038051891
ACH-000232	U251MG_CENTRAL_NERVOUS_SYSTEM	docetaxel	148124	0.038114543
ACH-000312	SKNBE2_AUTONOMIC_GANGLIA	docetaxel	148124	0.038189165
ACH-000221	SNU398_LIVER	paclitaxel	36314	0.038202578
ACH-000627	LCLC103H_LUNG	alvespimycin	5288674	0.038207463
ACH-000404	K029AX_SKIN	colchicine	6167	0.038354866
ACH-000730	SKMEL5_SKIN	NVP-AUY922	135539077	0.038356718
ACH-000562	HCC78_LUNG	cabazitaxel	129009963	0.038361511
ACH-000456	BCPAP_THYROID	filanesib	44224257	0.03849857
ACH-000812	COLO783_SKIN	filanesib	44224257	0.038528623
ACH-000288	BT549_BREAST	NVP-AUY922	135539077	0.038587454
ACH-000841	NCIH2087_LUNG	alvespimycin	5288674	0.038627598
ACH-000231	KALS1_CENTRAL_NERVOUS_SYSTEM	alvespimycin	5288674	0.038639792
ACH-000837	NCIH322_LUNG	docetaxel	148124	0.038689299
ACH-000553	SQ1_LUNG	FK-866	6914657	0.038771089
ACH-000945	NCIH650_LUNG	docetaxel	148124	0.03881806
ACH-000060	PANC1005_PANCREAS	docetaxel	148124	0.038846238
ACH-000047	GCIY_STOMACH	alvespimycin	5288674	0.038866496
ACH-000717	COLO680N_OESOPHAGUS	docetaxel	148124	0.03889536
ACH-000776	ONS76_CENTRAL_NERVOUS_SYSTEM	alvespimycin	5288674	0.038901303
ACH-000013	ONCODG1_OVARY	ganetespib	135564985	0.038921855

ACH-000853	NCIH661_LUNG	NVP-AUY922	135539077	0.039075428
ACH-000909	JHUEM2_ENDOMETRIUM	BNC105	24786555	0.039076466
ACH-000270	HPAC_PANCREAS	colchicine	6167	0.039146249
ACH-000488	TE11_OESOPHAGUS	FK-866	6914657	0.039156952
ACH-000845	NCIH1373_LUNG	ganetespib	135564985	0.039281285
ACH-000890	SW1271_LUNG	alvespimycin	5288674	0.039287171
ACH-000880	AGS_STOMACH	docetaxel	148124	0.039302756
ACH-000393	HLF_LIVER	alvespimycin	5288674	0.039327829
ACH-000307	PK1_PANCREAS	filanesib	44224257	0.039351509
ACH-000563	EBC1_LUNG	FK-866	6914657	0.039374785
ACH-000232	U251MG_CENTRAL_NERVOUS_SYSTEM	filanesib	44224257	0.039375418
ACH-000322	HT144_SKIN	docetaxel	148124	0.039475354
ACH-000493	SNU423_LIVER	alvespimycin	5288674	0.039480128
ACH-000450	MELHO_SKIN	docetaxel	148124	0.039603582
ACH-000954	HEC1A_ENDOMETRIUM	alvespimycin	5288674	0.039629642
ACH-000614	RVH421_SKIN	docetaxel	148124	0.039632022
ACH-000332	YAPC_PANCREAS	BI-2536	11364421	0.039742542
ACH-000971	HCT116_LARGE_INTESTINE	alvespimycin	5288674	0.039797107
ACH-000849	MDAMB468_BREAST	alvespimycin	5288674	0.039875596
ACH-000553	SQ1_LUNG	filanesib	44224257	0.039910237
ACH-000503	BICR16_UPPER_AERODIGESTIVE_TRACT	FK-866	6914657	0.040017184
ACH-000201	A204_SOFT_TISSUE	filanesib	44224257	0.040084118
ACH-000685	L33_PANCREAS	alvespimycin	5288674	0.040125644
ACH-000651	SW620_LARGE_INTESTINE	docetaxel	148124	0.040163914
ACH-000849	MDAMB468_BREAST	paclitaxel	36314	0.040202646
ACH-000090	PC3_PROSTATE	paclitaxel	36314	0.040227701
ACH-000778	HSC3_UPPER_AERODIGESTIVE_TRACT	paclitaxel	36314	0.040273136
ACH-000008	A101D_SKIN	docetaxel	148124	0.040289763
ACH-000845	NCIH1373_LUNG	NVP-AUY922	135539077	0.040374277
ACH-000587	NCIH1975_LUNG	docetaxel	148124	0.040378313
ACH-000201	A204_SOFT_TISSUE	paclitaxel	36314	0.040393621
ACH-000632	HS944T_SKIN	ganetespib	135564985	0.040413681
ACH-000853	NCIH661_LUNG	docetaxel	148124	0.040424179
ACH-000974	SNGM_ENDOMETRIUM	alvespimycin	5288674	0.040434438
ACH-000736	SNU601_STOMACH	alvespimycin	5288674	0.04043624
ACH-000396	J82_URINARY_TRACT	filanesib	44224257	0.040461078
ACH-000566	SW1710_URINARY_TRACT	NVP-AUY922	135539077	0.040469299
ACH-000667	HCC44_LUNG	FK-866	6914657	0.040482277
ACH-000723	YD10B_UPPER_AERODIGESTIVE_TRACT	NVP-AUY922	135539077	0.040627074
ACH-000562	HCC78_LUNG	docetaxel	148124	0.040646264
ACH-000720	TCCSUP_URINARY_TRACT	filanesib	44224257	0.04067027
ACH-000232	U251MG_CENTRAL_NERVOUS_SYSTEM	NVP-AUY922	135539077	0.040675268
ACH-000947	OVK18_OVARY	paclitaxel	36314	0.040682912

ACH-000603	BEN_LUNG	LY2606368	46700756	0.040697662
ACH-000994	HEC59_ENDOMETRIUM	NVP-AUY922	135539077	0.040747949
ACH-000749	DMS273_LUNG	alvespimycin	5288674	0.040778894
ACH-000335	MSTO211H_PLEURA	alvespimycin	5288674	0.040788977
ACH-000878	HCC15_LUNG	FK-866	6914657	0.040797334
ACH-000090	PC3_PROSTATE	NVP-AUY922	135539077	0.040835094
ACH-000351	MKN1_STOMACH	ganetespib	135564985	0.040895045
ACH-000863	DBTRG05MG_CENTRAL_NERVOUS_SYSTEM	alvespimycin	5288674	0.040936553
ACH-000019	MCF7_BREAST	filanesib	44224257	0.040962776
ACH-000318	TE10_OESOPHAGUS	alvespimycin	5288674	0.041042659
ACH-000954	HEC1A_ENDOMETRIUM	NVP-AUY922	135539077	0.041057205
ACH-000456	BCPAP_THYROID	docetaxel	148124	0.041073792
ACH-000813	T3M10_LUNG	FK-866	6914657	0.041095904
ACH-000505	RKN_SOFT_TISSUE	bortezomib	387447	0.041131992
ACH-000833	RH30_SOFT_TISSUE	alvespimycin	5288674	0.041168914
ACH-000454	HCC95_LUNG	docetaxel	148124	0.041218841
ACH-000102	GMS10_CENTRAL_NERVOUS_SYSTEM	taltobulin	6918637	0.041279013
ACH-000599	PATU8902_PANCREAS	docetaxel	148124	0.041285745
ACH-000425	UACC62_SKIN	alvespimycin	5288674	0.041294223
ACH-000810	SKMEL30_SKIN	colchicine	6167	0.041348396
ACH-000335	MSTO211H_PLEURA	docetaxel	148124	0.041372962
ACH-000450	MELHO_SKIN	FK-866	6914657	0.04144013
ACH-000047	GCIY_STOMACH	MPI-0479605	46909588	0.041484122
ACH-000840	HCC366_LUNG	docetaxel	148124	0.041486358
ACH-000396	J82_URINARY_TRACT	NVP-AUY922	135539077	0.041544766
ACH-000407	SNU685_ENDOMETRIUM	alvespimycin	5288674	0.041568273
ACH-000012	HCC827_LUNG	docetaxel	148124	0.04158538
ACH-000765	WM983B_SKIN	alvespimycin	5288674	0.041586481
ACH-000805	COLO679_SKIN	docetaxel	148124	0.041593999
ACH-000014	HS294T_SKIN	docetaxel	148124	0.041604334
ACH-000972	HEC151_ENDOMETRIUM	docetaxel	148124	0.041758869
ACH-000749	DMS273_LUNG	ganetespib	135564985	0.041781687
ACH-000803	COLO668_LUNG	JNJ-26481585	11538455	0.041830221
ACH-000481	NCIH2170_LUNG	NVP-AUY922	135539077	0.041901086
ACH-000719	RMGI_OVARY	NSC-319726	5921672	0.041969095
ACH-000054	HT1080_SOFT_TISSUE	alvespimycin	5288674	0.0419764
ACH-000863	DBTRG05MG_CENTRAL_NERVOUS_SYSTEM	pralatrexate	148121	0.04199547
ACH-000833	RH30_SOFT_TISSUE	docetaxel	148124	0.041997303
ACH-000791	RERFLCAD1_LUNG	alvespimycin	5288674	0.042004339
ACH-001075	NCIH292_LUNG	alvespimycin	5288674	0.042079716
ACH-000924	NCIH2172_LUNG	docetaxel	148124	0.042094779
ACH-000966	IGROV1_OVARY	filanesib	44224257	0.04209576
ACH-000368	SNU1105_CENTRAL_NERVOUS_SYSTEM	filanesib	44224257	0.042156505

ACH-000715	SNU1214_UPPER_AERODIGESTIVE_TRACT	alvespimycin	5288674	0.042181815
ACH-000351	MKN1_STOMACH	filanesib	44224257	0.042273352
ACH-000579	UACC257_SKIN	colchicine	6167	0.042344845
ACH-000869	NCIH1568_LUNG	alvespimycin	5288674	0.04235494
ACH-000950	LOVO_LARGE_INTESTINE	alvespimycin	5288674	0.042358813
ACH-000628	NCIH596_LUNG	filanesib	44224257	0.042361939
ACH-000924	NCIH2172_LUNG	filanesib	44224257	0.042362352
ACH-000060	PANC1005_PANCREAS	FK-866	6914657	0.042367016
ACH-000846	FADU_UPPER_AERODIGESTIVE_TRACT	pralatrexate	148121	0.042370765
ACH-000231	KALS1_CENTRAL_NERVOUS_SYSTEM	paclitaxel	36314	0.042394957
ACH-000901	HCC1359_LUNG	FK-866	6914657	0.042396167
ACH-000787	LXF289_LUNG	docetaxel	148124	0.042440943
ACH-000449	MESSA_SOFT_TISSUE	filanesib	44224257	0.042605071
ACH-000911	NUGC3_STOMACH	ganetespib	135564985	0.042624489
ACH-000732	PECAPJ41CLONED2_UPPER_AERODIGESTIVE_TRACT	filanesib	44224257	0.042685535
ACH-000416	NCIH838_LUNG	paclitaxel	36314	0.04269707
ACH-000868	HCC1195_LUNG	docetaxel	148124	0.042715259
ACH-000351	MKN1_STOMACH	alvespimycin	5288674	0.042739359
ACH-000841	NCIH2087_LUNG	filanesib	44224257	0.042787684
ACH-000996	HEC251_ENDOMETRIUM	FK-866	6914657	0.042798991
ACH-000308	EFO21_OVARY	filanesib	44224257	0.042815011
ACH-000549	SNU1076_UPPER_AERODIGESTIVE_TRACT	alvespimycin	5288674	0.042819562
ACH-000305	ECGI10_OESOPHAGUS	alvespimycin	5288674	0.042848621
ACH-000502	TCCPAN2_PANCREAS	alvespimycin	5288674	0.042854951
ACH-000650	IGR37_SKIN	ganetespib	135564985	0.042919245
ACH-000414	NCIH1944_LUNG	FK-866	6914657	0.043011335
ACH-000840	HCC366_LUNG	alvespimycin	5288674	0.043018788
ACH-000449	MESSA_SOFT_TISSUE	ispinesib	6851740	0.04303122
ACH-000153	NCIH2052_PLEURA	AZD8330	16666708	0.043200405
ACH-000573	MDAMB436_BREAST	filanesib	44224257	0.043201736
ACH-000837	NCIH322_LUNG	alvespimycin	5288674	0.043264307
ACH-000312	SKNBE2_AUTONOMIC_GANGLIA	NVP-AUY922	135539077	0.04326598
ACH-000461	SNU1196_BILIARY_TRACT	filanesib	44224257	0.043305253
ACH-000232	U251MG_CENTRAL_NERVOUS_SYSTEM	ganetespib	135564985	0.043345008
ACH-000929	NCIH2110_LUNG	NVP-AUY922	135539077	0.043345199
ACH-000505	RKN_SOFT_TISSUE	alvespimycin	5288674	0.043362499
ACH-000853	NCIH661_LUNG	ganetespib	135564985	0.043419308
ACH-000212	CAL120_BREAST	alvespimycin	5288674	0.043429987
ACH-000813	T3M10_LUNG	NVP-AUY922	135539077	0.043487785
ACH-000318	TE10_OESOPHAGUS	NVP-AUY922	135539077	0.043488346
ACH-000456	BCPAP_THYROID	piperazine	4837	0.043528558
ACH-000860	NCIH358_LUNG	docetaxel	148124	0.043619425
ACH-000396	J82_URINARY_TRACT	FK-866	6914657	0.043623408

ACH-000685	L33_PANCREAS	ganetespib	135564985	0.043720529
ACH-000235	PANC0403_PANCREAS	alvespimycin	5288674	0.04373104
ACH-000796	MCAS_OVARY	pralatrexate	148121	0.04374329
ACH-000090	PC3_PROSTATE	pralatrexate	148121	0.043745881
ACH-000102	GMS10_CENTRAL_NERVOUS_SYSTEM	FK-866	6914657	0.043758925
ACH-000364	U2OS_BONE	filanesib	44224257	0.043765548
ACH-000182	SNU869_BILIARY_TRACT	alvespimycin	5288674	0.04378519
ACH-000308	EFO21_OVARY	docetaxel	148124	0.043821295
ACH-000984	HEC6_ENDOMETRIUM	docetaxel	148124	0.043835674
ACH-000976	HUCCT1_BILIARY_TRACT	NVP-AUY922	135539077	0.043870391
ACH-000308	EFO21_OVARY	alvespimycin	5288674	0.043939943
ACH-000361	SKHEP1_LIVER	pralatrexate	148121	0.043959657
ACH-000791	RERFLCAD1_LUNG	NVP-AUY922	135539077	0.043975564
ACH-000332	YAPC_PANCREAS	vindesine	40839	0.043995045
ACH-000312	SKNBE2_AUTONOMIC_GANGLIA	filanesib	44224257	0.044044649
ACH-000359	MG63_BONE	NVP-AUY922	135539077	0.044049498
ACH-000305	ECGI10_OESOPHAGUS	NVP-AUY922	135539077	0.044121813
ACH-000976	HUCCT1_BILIARY_TRACT	docetaxel	148124	0.04412564
ACH-000778	HSC3_UPPER_AERODIGESTIVE_TRACT	filanesib	44224257	0.044135961
ACH-000628	NCIH596_LUNG	docetaxel	148124	0.044143692
ACH-000955	SNU407_LARGE_INTESTINE	filanesib	44224257	0.044182533
ACH-000762	YD38_UPPER_AERODIGESTIVE_TRACT	FK-866	6914657	0.044202684
ACH-000264	CALU6_LUNG	dinaciclib	46926350	0.044249018
ACH-000228	BICR31_UPPER_AERODIGESTIVE_TRACT	filanesib	44224257	0.044317822
ACH-000182	SNU869_BILIARY_TRACT	ganetespib	135564985	0.044333695
ACH-000461	SNU1196_BILIARY_TRACT	alvespimycin	5288674	0.044359002
ACH-000717	COLO680N_OESOPHAGUS	filanesib	44224257	0.044365618
ACH-000628	NCIH596_LUNG	alvespimycin	5288674	0.044394369
ACH-000359	MG63_BONE	filanesib	44224257	0.044406747
ACH-000582	COLO741_SKIN	alvespimycin	5288674	0.044407064
ACH-000835	GCT_SOFT_TISSUE	filanesib	44224257	0.044449262
ACH-000280	SNU840_OVARY	paclitaxel	36314	0.044450804
ACH-000954	HEC1A_ENDOMETRIUM	ganetespib	135564985	0.04448413
ACH-000869	NCIH1568_LUNG	NVP-AUY922	135539077	0.044535619
ACH-000785	NCIH2126_LUNG	docetaxel	148124	0.044552419
ACH-000678	MKN7_STOMACH	alvespimycin	5288674	0.044562823
ACH-000991	SNU81_LARGE_INTESTINE	docetaxel	148124	0.044659182
ACH-000427	NCIN87_STOMACH	FK-866	6914657	0.044689313
ACH-000141	SNU308_BILIARY_TRACT	NSC-319726	5921672	0.044748029
ACH-000231	KALS1_CENTRAL_NERVOUS_SYSTEM	ganetespib	135564985	0.044754338
ACH-000994	HEC59_ENDOMETRIUM	ganetespib	135564985	0.044817301
ACH-000882	IGR1_SKIN	alvespimycin	5288674	0.044834827
ACH-000684	KMRC1_KIDNEY	colchicine	6167	0.044840898

ACH-000090	PC3_PROSTATE	ganetespib	135564985	0.044850811
ACH-000599	PATU8902_PANCREAS	FK-866	6914657	0.044953447
ACH-000805	COLO679_SKIN	BNC105	24786555	0.045022366
ACH-000833	RH30_SOFT_TISSUE	bortezomib	387447	0.045040052
ACH-000535	BXPC3_PANCREAS	epothilone-b	129010071	0.04504641
ACH-000599	PATU8902_PANCREAS	filanesib	44224257	0.045120422
ACH-000408	TE5_OESOPHAGUS	alvespimycin	5288674	0.045140976
ACH-000147	T47D_BREAST	filanesib	44224257	0.045161877
ACH-000599	PATU8902_PANCREAS	alvespimycin	5288674	0.045167574
ACH-000308	EFO21_OVARY	FK-866	6914657	0.045169038
ACH-000035	NCIH1650_LUNG	docetaxel	148124	0.045174314
ACH-000396	J82_URINARY_TRACT	pralatrexate	148121	0.045188736
ACH-000845	NCIH1373_LUNG	paclitaxel	36314	0.045271166
ACH-000023	PATU8988T_PANCREAS	pralatrexate	148121	0.045287879
ACH-000393	HLF_LIVER	paclitaxel	36314	0.045293139
ACH-000721	HMC18_BREAST	pralatrexate	148121	0.045318283
ACH-000461	SNU1196_BILIARY_TRACT	vincristine	5388993	0.045345743
ACH-000237	JHOM1_OVARY	pralatrexate	148121	0.045358931
ACH-000502	TCCPAN2_PANCREAS	selinexor	71481097	0.045497729
ACH-000035	NCIH1650_LUNG	vindesine	40839	0.045540017
ACH-000650	IGR37_SKIN	NVP-AUY922	135539077	0.045553441
ACH-000102	GMS10_CENTRAL_NERVOUS_SYSTEM	alvespimycin	5288674	0.045608221
ACH-000945	NCIH650_LUNG	pralatrexate	148121	0.045680118
ACH-000886	NCIH2009_LUNG	docetaxel	148124	0.045728338
ACH-000423	SKMEL3_SKIN	alvespimycin	5288674	0.045742137
ACH-000118	HUPT3_PANCREAS	FK-866	6914657	0.045813079
ACH-000097	ZR751_BREAST	docetaxel	148124	0.045853316
ACH-001239	WM2664_SKIN	colchicine	6167	0.045869364
ACH-000500	SNU46_UPPER_AERODIGESTIVE_TRACT	alvespimycin	5288674	0.045869422
ACH-000207	DETROIT562_UPPER_AERODIGESTIVE_TRACT	filanesib	44224257	0.045879353
ACH-000582	COLO741_SKIN	ganetespib	135564985	0.045880896
ACH-000805	COLO679_SKIN	alvespimycin	5288674	0.045938565
ACH-000277	HCC1419_BREAST	alvespimycin	5288674	0.046068693
ACH-000035	NCIH1650_LUNG	bortezomib	387447	0.046070208
ACH-000878	HCC15_LUNG	pralatrexate	148121	0.04607466
ACH-000853	NCIH661_LUNG	alvespimycin	5288674	0.046131602
ACH-000720	TCCSUP_URINARY_TRACT	docetaxel	148124	0.046144302
ACH-000147	T47D_BREAST	NVP-AUY922	135539077	0.046175842
ACH-000869	NCIH1568_LUNG	docetaxel	148124	0.046181565
ACH-000879	MFE296_ENDOMETRIUM	alvespimycin	5288674	0.046192409
ACH-000324	JHOC5_OVARY	alvespimycin	5288674	0.046196546
ACH-000837	NCIH322_LUNG	bortezomib	387447	0.046246483
ACH-000845	NCIH1373_LUNG	pralatrexate	148121	0.046322229

ACH-000856	CAL51_BREAST	JNJ-26481585	11538455	0.046360243
ACH-000014	HS294T_SKIN	alvespimycin	5288674	0.046361376
ACH-000880	AGS_STOMACH	paclitaxel	36314	0.046369348
ACH-000885	TOV21G_OVARY	pralatrexate	148121	0.046376253
ACH-000994	HEC59_ENDOMETRIUM	paclitaxel	36314	0.0464072
ACH-000147	T47D_BREAST	docetaxel	148124	0.046425055
ACH-000954	HEC1A_ENDOMETRIUM	paclitaxel	36314	0.046464785
ACH-000318	TE10_OESOPHAGUS	filanesib	44224257	0.046541724
ACH-000212	CAL120_BREAST	filanesib	44224257	0.04659381
ACH-000142	CAL29_URINARY_TRACT	pralatrexate	148121	0.046608841
ACH-000332	YAPC_PANCREAS	alvespimycin	5288674	0.046653526
ACH-000766	NCIH1648_LUNG	pralatrexate	148121	0.046751007
ACH-000991	SNU81_LARGE_INTESTINE	alvespimycin	5288674	0.046805897
ACH-000457	CAL54_KIDNEY	selinexor	71481097	0.046808356
ACH-000359	MG63_BONE	docetaxel	148124	0.046852152
ACH-000312	SKNBE2_AUTONOMIC_GANGLIA	ganetespib	135564985	0.046877401
ACH-000990	HEC108_ENDOMETRIUM	alvespimycin	5288674	0.046884551
ACH-000749	DMS273_LUNG	pralatrexate	148121	0.046945195
ACH-000221	SNU398_LIVER	ispinesib	6851740	0.04695877
ACH-000837	NCIH322_LUNG	FK-866	6914657	0.046967502
ACH-000768	MDAMB231_BREAST	filanesib	44224257	0.046984904
ACH-000843	HARA_LUNG	alvespimycin	5288674	0.046989445
ACH-000791	RERFLCAD1_LUNG	docetaxel	148124	0.046995138
ACH-000318	TE10_OESOPHAGUS	ganetespib	135564985	0.047007686
ACH-000182	SNU869_BILIARY_TRACT	filanesib	44224257	0.047015267
ACH-000561	TT_OESOPHAGUS	pralatrexate	148121	0.04701949
ACH-000019	MCF7_BREAST	alvespimycin	5288674	0.04702702
ACH-000967	SNUC2A_LARGE_INTESTINE	filanesib	44224257	0.047034933
ACH-000472	HSC2_UPPER_AERODIGESTIVE_TRACT	paclitaxel	36314	0.047068531
ACH-000967	SNUC2A_LARGE_INTESTINE	docetaxel	148124	0.047068594
ACH-000318	TE10_OESOPHAGUS	docetaxel	148124	0.04707679
ACH-000955	SNU407_LARGE_INTESTINE	alvespimycin	5288674	0.047087526
ACH-000565	RCM1_LARGE_INTESTINE	ganetespib	135564985	0.047105359
ACH-000776	ONS76_CENTRAL_NERVOUS_SYSTEM	docetaxel	148124	0.047133128
ACH-000420	SNU449_LIVER	bortezomib	387447	0.047158974
ACH-000688	OV7_OVARY	NSC-319726	5921672	0.047172285
ACH-000138	CFPAC1_PANCREAS	alvespimycin	5288674	0.047183987
ACH-000719	RMGI_OVARY	docetaxel	148124	0.04721528
ACH-000221	SNU398_LIVER	pralatrexate	148121	0.047215585
ACH-000723	YD10B_UPPER_AERODIGESTIVE_TRACT	ganetespib	135564985	0.047240997
ACH-000417	PANC0813_PANCREAS	docetaxel	148124	0.047276837
ACH-000091	OV56_OVARY	ispinesib	6851740	0.047307233
ACH-000903	FTC133_THYROID	alvespimycin	5288674	0.047333395

ACH-000685	L33_PANCREAS	docetaxel	148124	0.04739509
ACH-000849	MDAMB468_BREAST	NVP-AUY922	135539077	0.047396822
ACH-000950	LOVO_LARGE_INTESTINE	NVP-AUY922	135539077	0.047454321
ACH-000141	SNU308_BILIARY_TRACT	bortezomib	387447	0.04746766
ACH-000853	NCIH661_LUNG	bortezomib	387447	0.047474835
ACH-000991	SNU81_LARGE_INTESTINE	filanesib	44224257	0.047504953
ACH-000605	TE6_OESOPHAGUS	pralatrexate	148121	0.047512794
ACH-000624	HCC1806_BREAST	filanesib	44224257	0.047541803
ACH-000450	MELHO_SKIN	filanesib	44224257	0.04754436
ACH-000312	SKNBE2_AUTONOMIC_GANGLIA	alvespimycin	5288674	0.0476179
ACH-000841	NCIH2087_LUNG	ganetespib	135564985	0.047622438
ACH-000532	SNU61_LARGE_INTESTINE	ganetespib	135564985	0.047639043
ACH-000542	HEYA8_OVARY	alvespimycin	5288674	0.047644337
ACH-000417	PANC0813_PANCREAS	filanesib	44224257	0.047711024
ACH-000008	A101D_SKIN	filanesib	44224257	0.047729232
ACH-000123	COV434_OVARY	ispinesib	6851740	0.0477496
ACH-000808	HUH28_BILIARY_TRACT	alvespimycin	5288674	0.047821438
ACH-000562	HCC78_LUNG	filanesib	44224257	0.047855033
ACH-000444	LU99_LUNG	pralatrexate	148121	0.047861916
ACH-000890	SW1271_LUNG	paclitaxel	36314	0.047915419
ACH-000427	NCIN87_STOMACH	paclitaxel	36314	0.047921958
ACH-000587	NCIH1975_LUNG	paclitaxel	36314	0.047930556
ACH-000359	MG63_BONE	alvespimycin	5288674	0.047964877
ACH-000012	HCC827_LUNG	filanesib	44224257	0.047977508
ACH-000791	RERFLCAD1_LUNG	ganetespib	135564985	0.047989419
ACH-000805	COLO679_SKIN	FK-866	6914657	0.047992486
ACH-000582	COLO741_SKIN	NVP-AUY922	135539077	0.047999234
ACH-000320	PSN1_PANCREAS	filanesib	44224257	0.048016712
ACH-000842	SW480_LARGE_INTESTINE	docetaxel	148124	0.048059429
ACH-000027	GOS3_CENTRAL_NERVOUS_SYSTEM	FK-866	6914657	0.048083731
ACH-000805	COLO679_SKIN	paclitaxel	36314	0.048206216
ACH-000735	PECAPJ49_UPPER_AERODIGESTIVE_TRACT	pralatrexate	148121	0.048248036
ACH-000764	SH10TC_STOMACH	pralatrexate	148121	0.04825444
ACH-000945	NCIH650_LUNG	alvespimycin	5288674	0.048280079
ACH-000888	NCIH1793_LUNG	FK-866	6914657	0.048319291
ACH-000359	MG63_BONE	vindesine	40839	0.048328764
ACH-000593	BC3C_URINARY_TRACT	NVP-AUY922	135539077	0.048389039
ACH-000189	RCC10RGB_KIDNEY	FK-866	6914657	0.048430482
ACH-000837	NCIH322_LUNG	NVP-AUY922	135539077	0.048435085
ACH-000046	ACHN_KIDNEY	alvespimycin	5288674	0.0484499
ACH-000423	SKMEL3_SKIN	FK-866	6914657	0.048460383
ACH-000880	AGS_STOMACH	vindesine	40839	0.048489712
ACH-000813	T3M10_LUNG	ispinesib	6851740	0.048509098

ACH-000384	SW780_URINARY_TRACT	selinexor	71481097	0.048514027
ACH-000133	HS729_SOFT_TISSUE	NVP-AUY922	135539077	0.048522475
ACH-000990	HEC108_ENDOMETRIUM	FK-866	6914657	0.04857765
ACH-000097	ZR751_BREAST	filanesib	44224257	0.048591274
ACH-000418	SW1353_BONE	NVP-AUY922	135539077	0.048600259
ACH-000833	RH30_SOFT_TISSUE	filanesib	44224257	0.048621974
ACH-000322	HT144_SKIN	combretastatin-A-4	5351344	0.048642328
ACH-000717	COLO680N_OESOPHAGUS	paclitaxel	36314	0.048643583
ACH-000138	CFPAC1_PANCREAS	NVP-AUY922	135539077	0.048677834
ACH-000613	HOS_BONE	pralatrexate	148121	0.048750507
ACH-000985	LS411N_LARGE_INTESTINE	MPI-0479605	46909588	0.04878469
ACH-000994	HEC59_ENDOMETRIUM	pralatrexate	148121	0.048803755
ACH-000572	G361_SKIN	alvespimycin	5288674	0.048833255
ACH-000678	MKN7_STOMACH	docetaxel	148124	0.04885078
ACH-000416	NCIH838_LUNG	filanesib	44224257	0.048871862
ACH-000843	HARA_LUNG	JNJ-26481585	11538455	0.048884938
ACH-000836	YD15_SALIVARY_GLAND	docetaxel	148124	0.048889056
ACH-000954	HEC1A_ENDOMETRIUM	pralatrexate	148121	0.048903787
ACH-000802	BFTC905_URINARY_TRACT	alvespimycin	5288674	0.048908176
ACH-000976	HUCCT1_BILIARY_TRACT	ganetespib	135564985	0.048927935
ACH-000895	CL34_LARGE_INTESTINE	filanesib	44224257	0.048960415
ACH-000117	EFM192A_BREAST	alvespimycin	5288674	0.048980418
ACH-000450	MELHO_SKIN	paclitaxel	36314	0.048987547
ACH-000946	HEC265_ENDOMETRIUM	pralatrexate	148121	0.048993602
ACH-000139	PANC0327_PANCREAS	NVP-AUY922	135539077	0.049016211
ACH-000008	A101D_SKIN	paclitaxel	36314	0.049040768
ACH-000232	U251MG_CENTRAL_NERVOUS_SYSTEM	dinaciclib	46926350	0.049063859
ACH-000454	HCC95_LUNG	paclitaxel	36314	0.049110719
ACH-000288	BT549_BREAST	pralatrexate	148121	0.049116259
ACH-000833	RH30_SOFT_TISSUE	paclitaxel	36314	0.049162739
ACH-000054	HT1080_SOFT_TISSUE	MPI-0479605	46909588	0.049177661
ACH-000161	CORL105_LUNG	LY2606368	46700756	0.0492125
ACH-000878	HCC15_LUNG	dinaciclib	46926350	0.049248644
ACH-000965	RL952_ENDOMETRIUM	pralatrexate	148121	0.04925456
ACH-000945	NCIH650_LUNG	paclitaxel	36314	0.049263523
ACH-000805	COLO679_SKIN	filanesib	44224257	0.049313435
ACH-000868	HCC1195_LUNG	filanesib	44224257	0.049319533
ACH-000868	HCC1195_LUNG	vindesine	40839	0.049331899
ACH-000950	LOVO_LARGE_INTESTINE	filanesib	44224257	0.049350935
ACH-000502	TCCPAN2_PANCREAS	vindesine	40839	0.049354325
ACH-000717	COLO680N_OESOPHAGUS	NVP-AUY922	135539077	0.049355418
ACH-000035	NCIH1650_LUNG	paclitaxel	36314	0.049390552
ACH-000223	HCC1937_BREAST	FK-866	6914657	0.049401857

ACH-000322	HT144_SKIN	paclitaxel	36314	0.049444808
ACH-000322	HT144_SKIN	pralatrexate	148121	0.049456984
ACH-000138	CFPAC1_PANCREAS	docetaxel	148124	0.049474062
ACH-000897	FTC238_THYROID	alvespimycin	5288674	0.049544426
ACH-000312	SKNBE2_AUTONOMIC_GANGLIA	paclitaxel	36314	0.049646068
ACH-000138	CFPAC1_PANCREAS	filanesib	44224257	0.04965417
ACH-000035	NCIH1650_LUNG	filanesib	44224257	0.049679215
ACH-000493	SNU423_LIVER	temsirolimus	129009966	0.049692681
ACH-000715	SNU1214_UPPER_AERODIGESTIVE_TRACT	docetaxel	148124	0.049716109
ACH-000662	CORL23_LUNG	pralatrexate	148121	0.04971807
ACH-000427	NCIN87_STOMACH	filanesib	44224257	0.049777717
ACH-000771	BICR56_UPPER_AERODIGESTIVE_TRACT	LY2606368	46700756	0.049783041
ACH-000967	SNUC2A_LARGE_INTESTINE	alvespimycin	5288674	0.049831488
ACH-000985	LS411N_LARGE_INTESTINE	docetaxel	148124	0.049865672
ACH-000155	SW1990_PANCREAS	docetaxel	148124	0.04988873
ACH-000609	SF126_CENTRAL_NERVOUS_SYSTEM	filanesib	44224257	0.049917377
ACH-000565	RCM1_LARGE_INTESTINE	NVP-AUY922	135539077	0.049935532
ACH-000587	NCIH1975_LUNG	filanesib	44224257	0.049947123
ACH-000019	MCF7_BREAST	docetaxel	148124	0.04999547
ACH-000667	HCC44_LUNG	pralatrexate	148121	0.050005403
ACH-000232	U251MG_CENTRAL_NERVOUS_SYSTEM	paclitaxel	36314	0.050039708
ACH-000549	SNU1076_UPPER_AERODIGESTIVE_TRACT	FK-866	6914657	0.050042822
ACH-000738	GB1_CENTRAL_NERVOUS_SYSTEM	filanesib	44224257	0.050065844
ACH-000814	HS939T_SKIN	docetaxel	148124	0.050087582
ACH-000955	SNU407_LARGE_INTESTINE	paclitaxel	36314	0.050128286
ACH-000235	PANC0403_PANCREAS	ganetespib	135564985	0.050129631
ACH-000542	HEYA8_OVARY	paclitaxel	36314	0.050149686
ACH-000647	TE1_OESOPHAGUS	FK-866	6914657	0.050167512
ACH-000685	L33_PANCREAS	NVP-AUY922	135539077	0.050174999
ACH-000749	DMS273_LUNG	ispinesib	6851740	0.050191847
ACH-000493	SNU423_LIVER	pralatrexate	148121	0.05020135
ACH-000155	SW1990_PANCREAS	filanesib	44224257	0.050211217
ACH-000308	EFO21_OVARY	NVP-AUY922	135539077	0.050247242
ACH-000368	SNU1105_CENTRAL_NERVOUS_SYSTEM	alvespimycin	5288674	0.050316278
ACH-000332	YAPC_PANCREAS	docetaxel	148124	0.050374913
ACH-000014	HS294T_SKIN	paclitaxel	36314	0.050375903
ACH-000235	PANC0403_PANCREAS	NVP-AUY922	135539077	0.050409839
ACH-000318	TE10_OESOPHAGUS	vindesine	40839	0.050410812
ACH-000210	CADOES1_BONE	colchicine	6167	0.050412642
ACH-000587	NCIH1975_LUNG	FK-866	6914657	0.050464785
ACH-001239	WM2664_SKIN	epothilone-b	129010071	0.050478977
ACH-000164	PANC1_PANCREAS	alvespimycin	5288674	0.050486755
ACH-000827	WM793_SKIN	docetaxel	148124	0.050585684

ACH-000161	CORL105_LUNG	alvespimycin	5288674	0.050680348
ACH-000408	TE5_OESOPHAGUS	docetaxel	148124	0.05073435
ACH-000201	A204_SOFT_TISSUE	ispinesib	6851740	0.050800639
ACH-000570	YKG1_CENTRAL_NERVOUS_SYSTEM	filanesib	44224257	0.050813673
ACH-000813	T3M10_LUNG	alvespimycin	5288674	0.050835224
ACH-000505	RKN_SOFT_TISSUE	FK-866	6914657	0.050861104
ACH-000808	HUH28_BILIARY_TRACT	FK-866	6914657	0.050865809
ACH-000542	HEYA8_OVARY	filanesib	44224257	0.050872846
ACH-000628	NCIH596_LUNG	paclitaxel	36314	0.050881932
ACH-000458	CJM_SKIN	alvespimycin	5288674	0.050886576
ACH-000035	NCIH1650_LUNG	vinblastine	13342	0.050887074
ACH-000014	HS294T_SKIN	filanesib	44224257	0.050912056
ACH-001318	PLCPRF5_LIVER	pralatrexate	148121	0.050973689
ACH-000976	HUCCT1_BILIARY_TRACT	filanesib	44224257	0.050997788
ACH-000593	BC3C_URINARY_TRACT	ganetespib	135564985	0.051009734
ACH-000941	HEC1B_ENDOMETRIUM	FK-866	6914657	0.051040901
ACH-000232	U251MG_CENTRAL_NERVOUS_SYSTEM	pralatrexate	148121	0.051059148
ACH-000035	NCIH1650_LUNG	NVP-AUY922	135539077	0.051085594
ACH-000614	RVH421_SKIN	paclitaxel	36314	0.051086334
ACH-000808	HUH28_BILIARY_TRACT	docetaxel	148124	0.051144861
ACH-000890	SW1271_LUNG	pralatrexate	148121	0.051150899
ACH-000359	MG63_BONE	ganetespib	135564985	0.051266007
ACH-000856	CAL51_BREAST	docetaxel	148124	0.051277614
ACH-000527	OVISE_OVARY	filanesib	44224257	0.051321301
ACH-000813	T3M10_LUNG	ganetespib	135564985	0.051329506
ACH-000750	LOXIMVI_SKIN	pralatrexate	148121	0.051342437
ACH-000603	BEN_LUNG	NVP-AUY922	135539077	0.051363371
ACH-000835	GCT_SOFT_TISSUE	alvespimycin	5288674	0.051384892
ACH-000856	CAL51_BREAST	alvespimycin	5288674	0.051399122
ACH-000846	FADU_UPPER_AERODIGESTIVE_TRACT	panobinostat	6918837	0.051404781
ACH-000785	NCIH2126_LUNG	paclitaxel	36314	0.051423802
ACH-000573	MDAMB436_BREAST	docetaxel	148124	0.05143585
ACH-000943	RKO_LARGE_INTESTINE	pralatrexate	148121	0.051437988
ACH-000147	T47D_BREAST	alvespimycin	5288674	0.0514639
ACH-000614	RVH421_SKIN	filanesib	44224257	0.051467249
ACH-000318	TE10_OESOPHAGUS	BNC105	24786555	0.051472408
ACH-000047	GCIY_STOMACH	pralatrexate	148121	0.051476786
ACH-000603	BEN_LUNG	alvespimycin	5288674	0.051477668
ACH-000827	WM793_SKIN	filanesib	44224257	0.051497004
ACH-000880	AGS_STOMACH	filanesib	44224257	0.051512625
ACH-000651	SW620_LARGE_INTESTINE	pralatrexate	148121	0.051513865
ACH-000837	NCIH322_LUNG	paclitaxel	36314	0.051576204
ACH-000732	PECAPJ41CLONED2_UPPER_AERODIGESTIVE_TRACT	pralatrexate	148121	0.051602058

ACH-000814	HS939T_SKIN	FK-866	6914657	0.051693282
ACH-000553	SQ1_LUNG	pralatrexate	148121	0.051712471
ACH-000421	SW837_LARGE_INTESTINE	bortezomib	387447	0.051753555
ACH-000678	MKN7_STOMACH	NVP-AUY922	135539077	0.051776033
ACH-000924	NCIH2172_LUNG	paclitaxel	36314	0.051795212
ACH-000060	PANC1005_PANCREAS	BNC105	24786555	0.051818757
ACH-000776	ONS76_CENTRAL_NERVOUS_SYSTEM	pralatrexate	148121	0.05184299
ACH-000985	LS411N_LARGE_INTESTINE	alvespimycin	5288674	0.051867422
ACH-000776	ONS76_CENTRAL_NERVOUS_SYSTEM	filanesib	44224257	0.051897319
ACH-000504	SNB75_CENTRAL_NERVOUS_SYSTEM	FK-866	6914657	0.05190776
ACH-000888	NCIH1793_LUNG	alvespimycin	5288674	0.051912378
ACH-000189	RCC10RGB_KIDNEY	pralatrexate	148121	0.051948995
ACH-000685	L33_PANCREAS	filanesib	44224257	0.051984757
ACH-000417	PANC0813_PANCREAS	FK-866	6914657	0.052033521
ACH-000107	CAPAN2_PANCREAS	selinexor	71481097	0.052048071
ACH-000528	ABC1_LUNG	pralatrexate	148121	0.052064765
ACH-000280	SNU840_OVARY	pralatrexate	148121	0.052069589
ACH-000648	NCIH28_PLEURA	colchicine	6167	0.052085402
ACH-000970	SNUC5_LARGE_INTESTINE	bortezomib	387447	0.052093844
ACH-000841	NCIH2087_LUNG	NVP-AUY922	135539077	0.052123734
ACH-000787	LXF289_LUNG	FK-866	6914657	0.052166054
ACH-000573	MDAMB436_BREAST	NVP-AUY922	135539077	0.052195327
ACH-000800	NCIH446_LUNG	alvespimycin	5288674	0.052212614
ACH-000842	SW480_LARGE_INTESTINE	vindesine	40839	0.052251057
ACH-000911	NUGC3_STOMACH	tanespimycin	6505803	0.052256668
ACH-000573	MDAMB436_BREAST	ganetespib	135564985	0.052288681
ACH-000445	KNS60_CENTRAL_NERVOUS_SYSTEM	pralatrexate	148121	0.052292675
ACH-000984	HEC6_ENDOMETRIUM	filanesib	44224257	0.052331297
ACH-001321	TT_THYROID	AZD8330	16666708	0.052379005
ACH-000860	NCIH358_LUNG	BNC105	24786555	0.052490551
ACH-000619	PECAPJ15_UPPER_AERODIGESTIVE_TRACT	pralatrexate	148121	0.052508109
ACH-000813	T3M10_LUNG	pralatrexate	148121	0.052529272
ACH-000504	SNB75_CENTRAL_NERVOUS_SYSTEM	filanesib	44224257	0.052589151
ACH-000191	BHT101_THYROID	filanesib	44224257	0.05260396
ACH-000593	BC3C_URINARY_TRACT	FK-866	6914657	0.052633103
ACH-000012	HCC827_LUNG	alvespimycin	5288674	0.052684464
ACH-000082	G292CLONEA141B1_BONE	NSC-319726	5921672	0.052723465
ACH-000496	NCIH1792_LUNG	pralatrexate	148121	0.052747127
ACH-000544	OE21_OESOPHAGUS	MPI-0479605	46909588	0.052749283
ACH-000808	HUH28_BILIARY_TRACT	filanesib	44224257	0.05279142
ACH-000013	ONCODG1_OVARY	pralatrexate	148121	0.052806894
ACH-000671	HUH6_LIVER	filanesib	44224257	0.052818672
ACH-000420	SNU449_LIVER	filanesib	44224257	0.05284604

ACH-000132	JHOS2_OVARY	BI-2536	11364421	0.052858468
ACH-000404	K029AX_SKIN	alvespimycin	5288674	0.052894823
ACH-000991	SNU81_LARGE_INTESTINE	NVP-AUY922	135539077	0.052902784
ACH-000768	MDAMB231_BREAST	alvespimycin	5288674	0.052908689
ACH-000397	TEN_ENDOMETRIUM	NVP-AUY922	135539077	0.05291655
ACH-000228	BICR31_UPPER_AERODIGESTIVE_TRACT	pralatrexate	148121	0.05293285
ACH-000811	SKOV3_OVARY	pralatrexate	148121	0.052952261
ACH-000849	MDAMB468_BREAST	pralatrexate	148121	0.052962148
ACH-000482	RERFLCKJ_LUNG	pralatrexate	148121	0.052976842
ACH-000066	HCC4006_LUNG	alvespimycin	5288674	0.05304375
ACH-000277	HCC1419_BREAST	FK-866	6914657	0.05307621
ACH-000352	HCC1428_BREAST	bortezomib	387447	0.053082836
ACH-000717	COLO680N_OESOPHAGUS	ganetespib	135564985	0.053135954
ACH-000599	PATU8902_PANCREAS	paclitaxel	36314	0.053149366
ACH-000450	MELHO_SKIN	pralatrexate	148121	0.053159353
ACH-000545	VMCUB1_URINARY_TRACT	filanesib	44224257	0.053175276
ACH-000393	HLF_LIVER	elesclomol	300471	0.0531794
ACH-000027	GOS3_CENTRAL_NERVOUS_SYSTEM	docetaxel	148124	0.053200984
ACH-000632	HS944T_SKIN	crystal-violet	3468	0.053201565
ACH-000138	CFPAC1_PANCREAS	ganetespib	135564985	0.053219694
ACH-000826	CAL12T_LUNG	alvespimycin	5288674	0.053248935
ACH-000132	JHOS2_OVARY	filanesib	44224257	0.053255963
ACH-000572	G361_SKIN	docetaxel	148124	0.053273841
ACH-000313	KMRC3_KIDNEY	alvespimycin	5288674	0.053281551
ACH-000997	HCT15_LARGE_INTESTINE	JNJ-26481585	11538455	0.053323932
ACH-000777	KYSE30_OESOPHAGUS	pralatrexate	148121	0.053364568
ACH-000046	ACHN_KIDNEY	docetaxel	148124	0.053366442
ACH-000133	HS729_SOFT_TISSUE	BI-2536	11364421	0.053383193
ACH-000209	SNU1079_BILIARY_TRACT	filanesib	44224257	0.053396162
ACH-000882	IGR1_SKIN	docetaxel	148124	0.053396389
ACH-000376	SF295_CENTRAL_NERVOUS_SYSTEM	LY2606368	46700756	0.053423074
ACH-000971	HCT116_LARGE_INTESTINE	pralatrexate	148121	0.053436135
ACH-000332	YAPC_PANCREAS	filanesib	44224257	0.053453573
ACH-000791	RERFLCAD1_LUNG	filanesib	44224257	0.053478487
ACH-000826	CAL12T_LUNG	FK-866	6914657	0.053543143
ACH-000647	TE1_OESOPHAGUS	bortezomib	387447	0.053562236
ACH-000532	SNU61_LARGE_INTESTINE	NVP-AUY922	135539077	0.053587319
ACH-000147	T47D_BREAST	ganetespib	135564985	0.053611205
ACH-000191	BHT101_THYROID	docetaxel	148124	0.053670938
ACH-000141	SNU308_BILIARY_TRACT	alvespimycin	5288674	0.053692908
ACH-000189	RCC10RGB_KIDNEY	filanesib	44224257	0.053712934
ACH-000562	HCC78_LUNG	paclitaxel	36314	0.053717858
ACH-000924	NCIH2172_LUNG	pralatrexate	148121	0.053729049

ACH-000603	BEN_LUNG	ganetespib	135564985	0.053732507
ACH-000573	MDAMB436_BREAST	alvespimycin	5288674	0.053750941
ACH-000408	TE5_OESOPHAGUS	NVP-AUY922	135539077	0.053836999
ACH-000968	COLO792_SKIN	docetaxel	148124	0.053842431
ACH-000447	NCIH2228_LUNG	FK-866	6914657	0.053869737
ACH-000097	ZR751_BREAST	paclitaxel	36314	0.053956898
ACH-000444	LU99_LUNG	ispinesib	6851740	0.053966047
ACH-000397	TEN_ENDOMETRIUM	docetaxel	148124	0.053978264
ACH-000280	SNU840_OVARY	ispinesib	6851740	0.054122315
ACH-000209	SNU1079_BILIARY_TRACT	FK-866	6914657	0.054148843
ACH-000447	NCIH2228_LUNG	ganetespib	135564985	0.054166675
ACH-000042	PANC0203_PANCREAS	docetaxel	148124	0.054176409
ACH-000264	CALU6_LUNG	pralatrexate	148121	0.054188115
ACH-000332	YAPC_PANCREAS	ganetespib	135564985	0.054213078
ACH-000661	WM1799_SKIN	docetaxel	148124	0.054260011
ACH-000423	SKMEL3_SKIN	bortezomib	387447	0.054285425
ACH-000097	ZR751_BREAST	NVP-AUY922	135539077	0.054323601
ACH-000527	OVISE_OVARY	docetaxel	148124	0.054324437
ACH-000609	SF126_CENTRAL_NERVOUS_SYSTEM	alvespimycin	5288674	0.054363945
ACH-000895	CL34_LARGE_INTESTINE	docetaxel	148124	0.0544078
ACH-000643	HDQP1_BREAST	FK-866	6914657	0.054410408
ACH-000027	GOS3_CENTRAL_NERVOUS_SYSTEM	alvespimycin	5288674	0.054470138
ACH-000950	LOVO_LARGE_INTESTINE	docetaxel	148124	0.054478134
ACH-000139	PANC0327_PANCREAS	vindesine	40839	0.05449593
ACH-000913	ESS1_ENDOMETRIUM	NVP-AUY922	135539077	0.054539463
ACH-000685	L33_PANCREAS	FK-866	6914657	0.054544041
ACH-000603	BEN_LUNG	FK-866	6914657	0.054551877
ACH-000587	NCIH1975_LUNG	pralatrexate	148121	0.054556639
ACH-000186	NCIH2444_LUNG	BNC105	24786555	0.054565888
ACH-000308	EFO21_OVARY	ganetespib	135564985	0.054699137
ACH-000955	SNU407_LARGE_INTESTINE	NVP-AUY922	135539077	0.054721478
ACH-000562	HCC78_LUNG	epothilone-b	129010071	0.054727776
ACH-000500	SNU46_UPPER_AERODIGESTIVE_TRACT	pralatrexate	148121	0.054737704
ACH-000397	TEN_ENDOMETRIUM	alvespimycin	5288674	0.054828192
ACH-000376	SF295_CENTRAL_NERVOUS_SYSTEM	alvespimycin	5288674	0.05483955
ACH-000565	RCM1_LARGE_INTESTINE	alvespimycin	5288674	0.054841774
ACH-000060	PANC1005_PANCREAS	pralatrexate	148121	0.054845368
ACH-000783	CAMA1_BREAST	paclitaxel	36314	0.05485401
ACH-000783	CAMA1_BREAST	alvespimycin	5288674	0.054869996
ACH-000895	CL34_LARGE_INTESTINE	ganetespib	135564985	0.054910619
ACH-000318	TE10_OESOPHAGUS	paclitaxel	36314	0.054922741
ACH-000677	SW1573_LUNG	filanesib	44224257	0.054940491
ACH-000132	JHOS2_OVARY	docetaxel	148124	0.054972505

ACH-000161	CORL105_LUNG	FK-866	6914657	0.055103222
ACH-000097	ZR751_BREAST	alvespimycin	5288674	0.055127407
ACH-000860	NCIH358_LUNG	paclitaxel	36314	0.055181627
ACH-000911	NUGC3_STOMACH	FK-866	6914657	0.055187712
ACH-000222	ASPC1_PANCREAS	alvespimycin	5288674	0.055209203
ACH-000237	JHOM1_OVARY	ispinesib	6851740	0.055231494
ACH-000657	A2780_OVARY	rigosertib	6918736	0.055253553
ACH-000118	HUPT3_PANCREAS	filanesib	44224257	0.055263569
ACH-000164	PANC1_PANCREAS	vindesine	40839	0.055322013
ACH-000479	KNS81_CENTRAL_NERVOUS_SYSTEM	NVP-AUY922	135539077	0.055341942
ACH-000959	SNUC4_LARGE_INTESTINE	alvespimycin	5288674	0.055396961
ACH-000447	NCIH2228_LUNG	NVP-AUY922	135539077	0.055434923
ACH-000332	YAPC_PANCREAS	NVP-AUY922	135539077	0.055466458
ACH-000222	ASPC1_PANCREAS	NVP-AUY922	135539077	0.055496678
ACH-000351	MKN1_STOMACH	pralatrexate	148121	0.055513342
ACH-000808	HUH28_BILIARY_TRACT	paclitaxel	36314	0.055538103
ACH-001075	NCIH292_LUNG	filanesib	44224257	0.055585293
ACH-000374	HCC1143_BREAST	docetaxel	148124	0.055592902
ACH-000822	SKMEL24_SKIN	alvespimycin	5288674	0.055605585
ACH-000147	T47D_BREAST	paclitaxel	36314	0.055646443
ACH-000768	MDAMB231_BREAST	docetaxel	148124	0.055682694
ACH-000647	TE1_OESOPHAGUS	filanesib	44224257	0.055784247
ACH-000880	AGS_STOMACH	pralatrexate	148121	0.055796281
ACH-000685	L33_PANCREAS	tanespimycin	6505803	0.055810511
ACH-000609	SF126_CENTRAL_NERVOUS_SYSTEM	docetaxel	148124	0.05588968
ACH-000418	SW1353_BONE	docetaxel	148124	0.055940913
ACH-000875	NCIH2347_LUNG	docetaxel	148124	0.055991335
ACH-000719	RMGI_OVARY	filanesib	44224257	0.05602597
ACH-000117	EFM192A_BREAST	paclitaxel	36314	0.056026276
ACH-000521	NCIH2030_LUNG	pralatrexate	148121	0.056026587
ACH-000628	NCIH596_LUNG	LY2606368	46700756	0.056127915
ACH-000941	HEC1B_ENDOMETRIUM	NVP-AUY922	135539077	0.056165674
ACH-000853	NCIH661_LUNG	pralatrexate	148121	0.056186342
ACH-000776	ONS76_CENTRAL_NERVOUS_SYSTEM	paclitaxel	36314	0.056255836
ACH-000898	SNU719_STOMACH	docetaxel	148124	0.056278254
ACH-000950	LOVO_LARGE_INTESTINE	bortezomib	387447	0.056299328
ACH-000359	MG63_BONE	paclitaxel	36314	0.056311456
ACH-000504	SNB75_CENTRAL_NERVOUS_SYSTEM	alvespimycin	5288674	0.056352141
ACH-000868	HCC1195_LUNG	combretastatin-A-4	5351344	0.056365425
ACH-000359	MG63_BONE	dinaciclib	46926350	0.056378396
ACH-000997	HCT15_LARGE_INTESTINE	NVP-AUY922	135539077	0.056388907
ACH-000421	SW837_LARGE_INTESTINE	vindesine	40839	0.056408916
ACH-000456	BCPAP_THYROID	alvespimycin	5288674	0.05641752

ACH-000244	DKMG_CENTRAL_NERVOUS_SYSTEM	FK-866	6914657	0.056496201
ACH-000329	CCFSTTG1_CENTRAL_NERVOUS_SYSTEM	docetaxel	148124	0.056580369
ACH-000882	IGR1_SKIN	filanesib	44224257	0.056687763
ACH-000628	NCIH596_LUNG	pralatrexate	148121	0.056697493
ACH-000118	HUPT3_PANCREAS	paclitaxel	36314	0.05673333
ACH-000836	YD15_SALIVARY_GLAND	filanesib	44224257	0.056736919
ACH-000619	PECAPJ15_UPPER_AERODIGESTIVE_TRACT	ispinesib	6851740	0.056789096
ACH-000066	HCC4006_LUNG	filanesib	44224257	0.056796596
ACH-000632	HS944T_SKIN	tanespimycin	6505803	0.056801436
ACH-000899	WM88_SKIN	alvespimycin	5288674	0.05689335
ACH-000266	SNU213_PANCREAS	taltobulin	6918637	0.056999469
ACH-000880	AGS_STOMACH	vinblastine	13342	0.057081274
ACH-000219	A375_SKIN	filanesib	44224257	0.057104816
ACH-000484	VMRCRCW_KIDNEY	colchicine	6167	0.057130308
ACH-000679	OE19_OESOPHAGUS	filanesib	44224257	0.057134232
ACH-000841	NCIH2087_LUNG	BAY-87-2243	67377767	0.057137045
ACH-000846	FADU_UPPER_AERODIGESTIVE_TRACT	litronesib	25167017	0.057189583
ACH-000307	PK1_PANCREAS	pralatrexate	148121	0.057196814
ACH-000565	RCM1_LARGE_INTESTINE	filanesib	44224257	0.057245134
ACH-000868	HCC1195_LUNG	paclitaxel	36314	0.057252575
ACH-000471	LI7_LIVER	alvespimycin	5288674	0.057254836
ACH-000278	COV362_OVARY	filanesib	44224257	0.05726036
ACH-000885	TOV21G_OVARY	rigosertib	6918736	0.057261266
ACH-000570	YKG1_CENTRAL_NERVOUS_SYSTEM	alvespimycin	5288674	0.057266654
ACH-000441	SH4_SKIN	cabazitaxel	129009963	0.057310195
ACH-000669	SW900_LUNG	filanesib	44224257	0.057323185
ACH-000416	NCIH838_LUNG	pralatrexate	148121	0.057376904
ACH-000976	HUCCT1_BILIARY_TRACT	pralatrexate	148121	0.057388322
ACH-000603	BEN_LUNG	pralatrexate	148121	0.057413931
ACH-000066	HCC4006_LUNG	NVP-AUY922	135539077	0.05751086
ACH-000669	SW900_LUNG	FK-866	6914657	0.05751578
ACH-000756	GI1_CENTRAL_NERVOUS_SYSTEM	tanespimycin	6505803	0.057534806
ACH-000893	NCIH1651_LUNG	selinexor	71481097	0.057559934
ACH-000397	TEN_ENDOMETRIUM	ganetespib	135564985	0.057567042
ACH-000054	HT1080_SOFT_TISSUE	pralatrexate	148121	0.057584004
ACH-000849	MDAMB468_BREAST	ganetespib	135564985	0.057643521
ACH-000678	MKN7_STOMACH	filanesib	44224257	0.057647285
ACH-000308	EFO21_OVARY	paclitaxel	36314	0.057711684
ACH-000685	L33_PANCREAS	paclitaxel	36314	0.057743792
ACH-000347	QGP1_PANCREAS	alvespimycin	5288674	0.057754955
ACH-000972	HEC151_ENDOMETRIUM	pralatrexate	148121	0.057765878
ACH-000765	WM983B_SKIN	tanespimycin	6505803	0.057783382
ACH-000305	ECGI10_OESOPHAGUS	ganetespib	135564985	0.057849401

ACH-000985	LS411N_LARGE_INTESTINE	filanesib	44224257	0.057875375
ACH-000738	GB1_CENTRAL_NERVOUS_SYSTEM	NVP-AUY922	135539077	0.057903709
ACH-000222	ASPC1_PANCREAS	filanesib	44224257	0.057915468
ACH-000966	IGROV1_OVARY	tanespimycin	6505803	0.057922884
ACH-000719	RMGI_OVARY	alvespimycin	5288674	0.057946783
ACH-000481	NCIH2170_LUNG	pralatrexate	148121	0.05800549
ACH-000505	RKN_SOFT_TISSUE	MPI-0479605	46909588	0.058024031
ACH-000846	FADU_UPPER_AERODIGESTIVE_TRACT	ispinesib	6851740	0.058028235
ACH-000967	SNUC2A_LARGE_INTESTINE	paclitaxel	36314	0.058036302
ACH-000423	SKMEL3_SKIN	ganetespib	135564985	0.058040259
ACH-000288	BT549_BREAST	temsirolimus	129009966	0.058088042
ACH-000985	LS411N_LARGE_INTESTINE	paclitaxel	36314	0.058158357
ACH-000913	ESS1_ENDOMETRIUM	JNJ-26481585	11538455	0.058189544
ACH-000787	LXF289_LUNG	vincristine	5388993	0.058248936
ACH-001318	PLCPRF5_LIVER	elesclomol	300471	0.058278755
ACH-000704	OAW42_OVARY	NVP-AUY922	135539077	0.058289844
ACH-000936	EFO27_OVARY	bortezomib	387447	0.058306423
ACH-000787	LXF289_LUNG	combretastatin-A-4	5351344	0.058310748
ACH-000132	JHOS2_OVARY	alvespimycin	5288674	0.058312489
ACH-000565	RCM1_LARGE_INTESTINE	docetaxel	148124	0.058337849
ACH-000468	PK45H_PANCREAS	filanesib	44224257	0.058344643
ACH-000447	NCIH2228_LUNG	alvespimycin	5288674	0.058348415
ACH-000178	HS766T_PANCREAS	alvespimycin	5288674	0.058365466
ACH-000437	SW1088_CENTRAL_NERVOUS_SYSTEM	pralatrexate	148121	0.058411778
ACH-000189	RCC10RGB_KIDNEY	alvespimycin	5288674	0.058426599
ACH-000842	SW480_LARGE_INTESTINE	NVP-AUY922	135539077	0.058428763
ACH-000814	HS939T_SKIN	BNC105	24786555	0.058440074
ACH-000897	FTC238_THYROID	pralatrexate	148121	0.058452074
ACH-000573	MDAMB436_BREAST	paclitaxel	36314	0.05847063
ACH-000880	AGS_STOMACH	LY2606368	46700756	0.058473444
ACH-000805	COLO679_SKIN	pralatrexate	148121	0.058507709
ACH-000423	SKMEL3_SKIN	docetaxel	148124	0.058556828
ACH-000476	JHH4_LIVER	filanesib	44224257	0.058564754
ACH-000661	WM1799_SKIN	filanesib	44224257	0.058580409
ACH-000046	ACHN_KIDNEY	filanesib	44224257	0.058684038
ACH-000153	NCIH2052_PLEURA	filanesib	44224257	0.058686955
ACH-000868	HCC1195_LUNG	BNC105	24786555	0.058716948
ACH-000456	BCPAP_THYROID	ispinesib	6851740	0.05872973
ACH-000715	SNU1214_UPPER_AERODIGESTIVE_TRACT	paclitaxel	36314	0.058800483
ACH-000990	HEC108_ENDOMETRIUM	ganetespib	135564985	0.058810337
ACH-000832	CAL27_UPPER_AERODIGESTIVE_TRACT	pralatrexate	148121	0.058817785
ACH-000159	OSRC2_KIDNEY	bortezomib	387447	0.058882785
ACH-000868	HCC1195_LUNG	vinblastine	13342	0.058887105

ACH-000627	LCLC103H_LUNG	elesclomol	300471	0.058916439
ACH-000488	TE11_OESOPHAGUS	pralatrexate	148121	0.058939184
ACH-000421	SW837_LARGE_INTESTINE	filanesib	44224257	0.058951813
ACH-000019	MCF7_BREAST	NVP-AUY922	135539077	0.058991525
ACH-000231	KALS1_CENTRAL_NERVOUS_SYSTEM	pralatrexate	148121	0.05900318
ACH-000903	FTC133_THYROID	docetaxel	148124	0.059013869
ACH-000517	SNU410_PANCREAS	alvespimycin	5288674	0.059122604
ACH-000418	SW1353_BONE	ganetespib	135564985	0.059202792
ACH-000329	CCFSTTG1_CENTRAL_NERVOUS_SYSTEM	FK-866	6914657	0.059211278
ACH-000679	OE19_OESOPHAGUS	docetaxel	148124	0.059216653
ACH-000924	NCIH2172_LUNG	dinaciclib	46926350	0.059240315
ACH-000147	T47D_BREAST	pralatrexate	148121	0.059260603
ACH-000954	HEC1A_ENDOMETRIUM	ispinesib	6851740	0.059262397
ACH-000895	CL34_LARGE_INTESTINE	NVP-AUY922	135539077	0.05932031
ACH-000191	BHT101_THYROID	alvespimycin	5288674	0.059332401
ACH-000579	UACC257_SKIN	FK-866	6914657	0.059342034
ACH-000717	COLO680N_OESOPHAGUS	pralatrexate	148121	0.059383449
ACH-000562	HCC78_LUNG	pralatrexate	148121	0.059388503
ACH-000161	CORL105_LUNG	NVP-AUY922	135539077	0.059406837
ACH-000347	QGP1_PANCREAS	cabazitaxel	129009963	0.059417444
ACH-000505	RKN_SOFT_TISSUE	ganetespib	135564985	0.05947078
ACH-000309	SKLU1_LUNG	FK-866	6914657	0.059514838
ACH-000652	SUIT2_PANCREAS	ganetespib	135564985	0.059521587
ACH-000019	MCF7_BREAST	paclitaxel	36314	0.059528425
ACH-000814	HS939T_SKIN	paclitaxel	36314	0.059635752
ACH-000331	ISTMES2_PLEURA	colchicine	6167	0.059717935
ACH-000472	HSC2_UPPER_AERODIGESTIVE_TRACT	pralatrexate	148121	0.059740042
ACH-000781	NCIH2023_LUNG	NVP-AUY922	135539077	0.059805542
ACH-000302	SNU1077_ENDOMETRIUM	NSC-319726	5921672	0.059870846
ACH-000359	MG63_BONE	BNC105	24786555	0.059906069
ACH-000479	KNS81_CENTRAL_NERVOUS_SYSTEM	alvespimycin	5288674	0.059910334
ACH-000694	TE9_OESOPHAGUS	pralatrexate	148121	0.059914351
ACH-000663	OVTOKO_OVARY	filanesib	44224257	0.059980878
ACH-000678	MKN7_STOMACH	paclitaxel	36314	0.059990023
ACH-000566	SW1710_URINARY_TRACT	pralatrexate	148121	0.06005374
ACH-000832	CAL27_UPPER_AERODIGESTIVE_TRACT	tanespimycin	6505803	0.060074153
ACH-000841	NCIH2087_LUNG	tanespimycin	6505803	0.060137728
ACH-000493	SNU423_LIVER	everolimus	6442177	0.060146362
ACH-000812	COLO783_SKIN	pralatrexate	148121	0.060219375
ACH-000888	NCIH1793_LUNG	NVP-AUY922	135539077	0.06023672
ACH-000788	A2058_SKIN	pralatrexate	148121	0.060295646
ACH-000791	RERFLCAD1_LUNG	FK-866	6914657	0.060343615
ACH-000549	SNU1076_UPPER_AERODIGESTIVE_TRACT	LY2606368	46700756	0.060395569

ACH-000117	EFM192A_BREAST	filanesib	44224257	0.060438209
ACH-000650	IGR37_SKIN	pralatrexate	148121	0.060469164
ACH-000582	COLO741_SKIN	pralatrexate	148121	0.060506077
ACH-000133	HS729_SOFT_TISSUE	pralatrexate	148121	0.060581433
ACH-000316	SNU886_LIVER	pralatrexate	148121	0.060627166
ACH-000502	TCCPAN2_PANCREAS	FK-866	6914657	0.060659619
ACH-000997	HCT15_LARGE_INTESTINE	alvespimycin	5288674	0.060701748
ACH-000244	DKMG_CENTRAL_NERVOUS_SYSTEM	docetaxel	148124	0.060718586
ACH-000456	BCPAP_THYROID	pralatrexate	148121	0.060823608
ACH-000019	MCF7_BREAST	elesclomol	300471	0.060853127
ACH-000976	HUCCT1_BILIARY_TRACT	paclitaxel	36314	0.060882288
ACH-000344	SNU668_STOMACH	FK-866	6914657	0.060923771
ACH-000091	OV56_OVARY	litronesib	25167017	0.060950814
ACH-000647	TE1_OESOPHAGUS	alvespimycin	5288674	0.060957352
ACH-000374	HCC1143_BREAST	NVP-AUY922	135539077	0.061022188
ACH-000681	A549_LUNG	docetaxel	148124	0.061027031
ACH-000880	AGS_STOMACH	BNC105	24786555	0.061077228
ACH-000255	LMSU_STOMACH	pralatrexate	148121	0.061120175
ACH-000219	A375_SKIN	alvespimycin	5288674	0.061137742
ACH-000792	BFTC909_KIDNEY	paclitaxel	36314	0.061141247
ACH-000384	SW780_URINARY_TRACT	FK-866	6914657	0.061156205
ACH-000102	GMS10_CENTRAL_NERVOUS_SYSTEM	filanesib	44224257	0.061159889
ACH-000397	TEN_ENDOMETRIUM	filanesib	44224257	0.061274153
ACH-000335	MSTO211H_PLEURA	tanespimycin	6505803	0.061313622
ACH-000957	LS180_LARGE_INTESTINE	alvespimycin	5288674	0.061328197
ACH-000479	KNS81_CENTRAL_NERVOUS_SYSTEM	docetaxel	148124	0.061348229
ACH-000974	SNGM_ENDOMETRIUM	pralatrexate	148121	0.06135373
ACH-000351	MKN1_STOMACH	ispinesib	6851740	0.061354217
ACH-000408	TE5_OESOPHAGUS	vindesine	40839	0.061357042
ACH-000008	A101D_SKIN	ganetespib	135564985	0.06137723
ACH-000417	PANC0813_PANCREAS	ganetespib	135564985	0.061384051
ACH-000420	SNU449_LIVER	NVP-AUY922	135539077	0.061388436
ACH-000159	OSRC2_KIDNEY	FK-866	6914657	0.061471061
ACH-000704	OAW42_OVARY	alvespimycin	5288674	0.061513531
ACH-000991	SNU81_LARGE_INTESTINE	ganetespib	135564985	0.061517753
ACH-000542	HEYA8_OVARY	pralatrexate	148121	0.061545206
ACH-000270	HPAC_PANCREAS	NVP-AUY922	135539077	0.061548349
ACH-000758	MKN74_STOMACH	taltobulin	6918637	0.061618287
ACH-000189	RCC10RGB_KIDNEY	dinaciclib	46926350	0.061626882
ACH-000343	NCIH522_LUNG	NVP-AUY922	135539077	0.061647894
ACH-000527	OVISE_OVARY	alvespimycin	5288674	0.061661111
ACH-000305	ECGI10_OESOPHAGUS	filanesib	44224257	0.061744212
ACH-000814	HS939T_SKIN	BI-2536	11364421	0.061809911

ACH-000813	T3M10_LUNG	piperazine	4837	0.061834802
ACH-000827	WM793_SKIN	paclitaxel	36314	0.061878468
ACH-000826	CAL12T_LUNG	docetaxel	148124	0.061885729
ACH-000191	BHT101_THYROID	pralatrexate	148121	0.061892049
ACH-000495	TUHR4TKB_KIDNEY	FK-866	6914657	0.061968186
ACH-000643	HDQP1_BREAST	filanesib	44224257	0.062047647
ACH-000833	RH30_SOFT_TISSUE	pralatrexate	148121	0.062051702
ACH-000648	NCIH28_PLEURA	alvespimycin	5288674	0.062059889
ACH-000332	YAPC_PANCREAS	paclitaxel	36314	0.062177716
ACH-000776	ONS76_CENTRAL_NERVOUS_SYSTEM	tanespimycin	6505803	0.062229256
ACH-000936	EFO27_OVARY	alvespimycin	5288674	0.06228192
ACH-000047	GCIY_STOMACH	tanespimycin	6505803	0.062285027
ACH-000393	HLF_LIVER	tanespimycin	6505803	0.062341844
ACH-000425	UACC62_SKIN	ispinesib	6851740	0.062454685
ACH-000847	HGC27_STOMACH	ispinesib	6851740	0.062489815
ACH-000985	LS411N_LARGE_INTESTINE	FK-866	6914657	0.062595407
ACH-000132	JHOS2_OVARY	bortezomib	387447	0.062609663
ACH-000132	JHOS2_OVARY	NVP-AUY922	135539077	0.062620429
ACH-000495	TUHR4TKB_KIDNEY	filanesib	44224257	0.062633001
ACH-000364	U2OS_BONE	floxuridine	5702211	0.062657228
ACH-000374	HCC1143_BREAST	filanesib	44224257	0.062660044
ACH-000042	PANC0203_PANCREAS	paclitaxel	36314	0.062704084
ACH-000082	G292CLONEA141B1_BONE	docetaxel	148124	0.062850863
ACH-000117	EFM192A_BREAST	docetaxel	148124	0.06294448
ACH-000738	GB1_CENTRAL_NERVOUS_SYSTEM	pralatrexate	148121	0.062947052
ACH-000685	L33_PANCREAS	dinaciclib	46926350	0.062966411
ACH-000417	PANC0813_PANCREAS	paclitaxel	36314	0.06298559
ACH-000423	SKMEL3_SKIN	NVP-AUY922	135539077	0.063040623
ACH-000277	HCC1419_BREAST	bortezomib	387447	0.06305123
ACH-000868	HCC1195_LUNG	alvespimycin	5288674	0.063092501
ACH-000572	G361_SKIN	NVP-AUY922	135539077	0.063095979
ACH-000368	SNU1105_CENTRAL_NERVOUS_SYSTEM	pralatrexate	148121	0.063106439
ACH-000768	MDAMB231_BREAST	BI-2536	11364421	0.063144878
ACH-000219	A375_SKIN	pralatrexate	148121	0.063166496
ACH-000842	SW480_LARGE_INTESTINE	vinblastine	13342	0.063171033
ACH-000792	BFTC909_KIDNEY	docetaxel	148124	0.06317124
ACH-000417	PANC0813_PANCREAS	pralatrexate	148121	0.063182248
ACH-000425	UACC62_SKIN	trametinib	11707110	0.063189984
ACH-000421	SW837_LARGE_INTESTINE	NVP-AUY922	135539077	0.063202032
ACH-000479	KNS81_CENTRAL_NERVOUS_SYSTEM	filanesib	44224257	0.063294128
ACH-000308	EFO21_OVARY	pralatrexate	148121	0.063341372
ACH-000207	DETROIT562_UPPER_AERODIGESTIVE_TRACT	pralatrexate	148121	0.063353314
ACH-000941	HEC1B_ENDOMETRIUM	ganetespib	135564985	0.063373001

ACH-000572	G361_SKIN	paclitaxel	36314	0.063406274
ACH-000847	HGC27_STOMACH	temoporfin	60751	0.063421552
ACH-000856	CAL51_BREAST	filanesib	44224257	0.063548913
ACH-000296	OUMS23_LARGE_INTESTINE	docetaxel	148124	0.063554334
ACH-001321	TT_THYROID	LY2606368	46700756	0.063646528
ACH-000736	SNU601_STOMACH	tanespimycin	6505803	0.063678118
ACH-000549	SNU1076_UPPER_AERODIGESTIVE_TRACT	tanespimycin	6505803	0.06369653
ACH-000155	SW1990_PANCREAS	pralatrexate	148121	0.063721188
ACH-000097	ZR751_BREAST	bortezomib	387447	0.063736934
ACH-000188	SCC25_UPPER_AERODIGESTIVE_TRACT	pralatrexate	148121	0.063823633
ACH-000384	SW780_URINARY_TRACT	alvespimycin	5288674	0.063827869
ACH-000648	NCIH28_PLEURA	ganetespib	135564985	0.06384156
ACH-000270	HPAC_PANCREAS	FK-866	6914657	0.063877481
ACH-000404	K029AX_SKIN	docetaxel	148124	0.063877716
ACH-000352	HCC1428_BREAST	epothilone-b	129010071	0.063906367
ACH-000744	NCIH1623_LUNG	ingenol-mebutate	6918670	0.063952135
ACH-000603	BEN_LUNG	docetaxel	148124	0.063957292
ACH-000783	CAMA1_BREAST	docetaxel	148124	0.063997204
ACH-000768	MDAMB231_BREAST	paclitaxel	36314	0.064072792
ACH-000955	SNU407_LARGE_INTESTINE	ganetespib	135564985	0.064092313
ACH-000885	TOV21G_OVARY	litronesib	25167017	0.064095496
ACH-000771	BICR56_UPPER_AERODIGESTIVE_TRACT	docetaxel	148124	0.064110135
ACH-000572	G361_SKIN	filanesib	44224257	0.064129805
ACH-000990	HEC108_ENDOMETRIUM	pralatrexate	148121	0.064164229
ACH-000278	COV362_OVARY	pralatrexate	148121	0.064196814
ACH-000270	HPAC_PANCREAS	cabazitaxel	129009963	0.064236649
ACH-000678	MKN7_STOMACH	ganetespib	135564985	0.064255435
ACH-000990	HEC108_ENDOMETRIUM	docetaxel	148124	0.06426663
ACH-000994	HEC59_ENDOMETRIUM	tanespimycin	6505803	0.064291719
ACH-000837	NCIH322_LUNG	pralatrexate	148121	0.064300723
ACH-000401	COLO800_SKIN	paclitaxel	36314	0.064356328
ACH-000504	SNB75_CENTRAL_NERVOUS_SYSTEM	NVP-AUY922	135539077	0.064387918
ACH-000222	ASPC1_PANCREAS	docetaxel	148124	0.064403631
ACH-000117	EFM192A_BREAST	NVP-AUY922	135539077	0.064457213
ACH-000814	HS939T_SKIN	pralatrexate	148121	0.064476448
ACH-000087	SKES1_BONE	alvespimycin	5288674	0.064480552
ACH-000936	EFO27_OVARY	docetaxel	148124	0.064518591
ACH-000138	CFPAC1_PANCREAS	pralatrexate	148121	0.064532692
ACH-000062	RERFLCMS_LUNG	tanespimycin	6505803	0.064533316
ACH-000882	IGR1_SKIN	NVP-AUY922	135539077	0.064573157
ACH-000882	IGR1_SKIN	ganetespib	135564985	0.064612078
ACH-000270	HPAC_PANCREAS	epothilone-b	129010071	0.064615201
ACH-000457	CAL54_KIDNEY	taltobulin	6918637	0.064636053

ACH-000869	NCIH1568_LUNG	tanespimycin	6505803	0.064641858
ACH-000842	SW480_LARGE_INTESTINE	FK-866	6914657	0.064649717
ACH-000791	RERFLCAD1_LUNG	tanespimycin	6505803	0.06466745
ACH-000833	RH30_SOFT_TISSUE	tanespimycin	6505803	0.064671114
ACH-000433	CAKI1_KIDNEY	docetaxel	148124	0.064687048
ACH-000967	SNUC2A_LARGE_INTESTINE	NVP-AUY922	135539077	0.064706051
ACH-000092	NCIH2452_PLEURA	FK-866	6914657	0.064730193
ACH-000479	KNS81_CENTRAL_NERVOUS_SYSTEM	ganetespib	135564985	0.06474342
ACH-000454	HCC95_LUNG	filanesib	44224257	0.064880024
ACH-000996	HEC251_ENDOMETRIUM	pralatrexate	148121	0.064895403
ACH-000774	RERFLCAD2_LUNG	alvespimycin	5288674	0.064928415
ACH-000421	SW837_LARGE_INTESTINE	pralatrexate	148121	0.064987612
ACH-000945	NCIH650_LUNG	ispinesib	6851740	0.06499345
ACH-000212	CAL120_BREAST	pralatrexate	148121	0.065038072
ACH-000842	SW480_LARGE_INTESTINE	ganetespib	135564985	0.065043868
ACH-000161	CORL105_LUNG	ganetespib	135564985	0.065053084
ACH-000277	HCC1419_BREAST	docetaxel	148124	0.065066862
ACH-000669	SW900_LUNG	paclitaxel	36314	0.065091691
ACH-000138	CFPAC1_PANCREAS	paclitaxel	36314	0.065122961
ACH-000376	SF295_CENTRAL_NERVOUS_SYSTEM	AZD8330	16666708	0.06514345
ACH-000781	NCIH2023_LUNG	ganetespib	135564985	0.065147663
ACH-000826	CAL12T_LUNG	paclitaxel	36314	0.065181547
ACH-000421	SW837_LARGE_INTESTINE	FK-866	6914657	0.065246701
ACH-000765	WM983B_SKIN	BNC105	24786555	0.065246845
ACH-000389	H4_CENTRAL_NERVOUS_SYSTEM	raltitrexed	135400182	0.065277077
ACH-000841	NCIH2087_LUNG	pralatrexate	148121	0.065312332
ACH-000107	CAPAN2_PANCREAS	vindesine	40839	0.065392615
ACH-000917	TE4_OESOPHAGUS	BNC105	24786555	0.065403952
ACH-000280	SNU840_OVARY	rigosertib	6918736	0.065472014
ACH-000186	NCIH2444_LUNG	pralatrexate	148121	0.065518752
ACH-000895	CL34_LARGE_INTESTINE	pralatrexate	148121	0.06555033
ACH-000352	HCC1428_BREAST	LY2606368	46700756	0.065584975
ACH-000141	SNU308_BILIARY_TRACT	carfilzomib	11556711	0.065593593
ACH-000625	HEP3B217_LIVER	colchicine	6167	0.06560283
ACH-000803	COLO668_LUNG	docetaxel	148124	0.065644358
ACH-000667	HCC44_LUNG	ispinesib	6851740	0.065663736
ACH-000719	RMGI_OVARY	paclitaxel	36314	0.065682051
ACH-000401	COLO800_SKIN	filanesib	44224257	0.065685236
ACH-000418	SW1353_BONE	paclitaxel	36314	0.06570541
ACH-000587	NCIH1975_LUNG	panobinostat	6918837	0.065849799
ACH-000582	COLO741_SKIN	tanespimycin	6505803	0.065855781
ACH-000582	COLO741_SKIN	elesclomol	300471	0.065876463
ACH-000648	NCIH28_PLEURA	AZD8330	16666708	0.065888619

ACH-000768	MDAMB231_BREAST	pralatrexate	148121	0.065916051
ACH-000352	HCC1428_BREAST	FK-866	6914657	0.066004296
ACH-000762	YD38_UPPER_AERODIGESTIVE_TRACT	tanespimycin	6505803	0.066074099
ACH-000280	SNU840_OVARY	litronesib	25167017	0.066106704
ACH-000244	DKMG_CENTRAL_NERVOUS_SYSTEM	paclitaxel	36314	0.066125371
ACH-000748	SJSA1_BONE	alvespimycin	5288674	0.066204589
ACH-000066	HCC4006_LUNG	ganetespib	135564985	0.066219298
ACH-000364	U2OS_BONE	ispinesib	6851740	0.066232771
ACH-000561	TT_OESOPHAGUS	tanespimycin	6505803	0.066243192
ACH-000625	HEP3B217_LIVER	pralatrexate	148121	0.066271099
ACH-000671	HUH6_LIVER	selinexor	71481097	0.066294329
ACH-000097	ZR751_BREAST	pralatrexate	148121	0.066354245
ACH-000950	LOVO_LARGE_INTESTINE	tanespimycin	6505803	0.066374458
ACH-000570	YKG1_CENTRAL_NERVOUS_SYSTEM	ispinesib	6851740	0.066383122
ACH-000092	NCIH2452_PLEURA	bortezomib	387447	0.066410984
ACH-000669	SW900_LUNG	NVP-AUY922	135539077	0.066505306
ACH-000018	T24_URINARY_TRACT	alvespimycin	5288674	0.066579416
ACH-000332	YAPC_PANCREAS	BNC105	24786555	0.066587992
ACH-000781	NCIH2023_LUNG	FK-866	6914657	0.066589075
ACH-000679	OE19_OESOPHAGUS	NVP-AUY922	135539077	0.066619835
ACH-001318	PLCPRF5_LIVER	rigosertib	6918736	0.066633494
ACH-000441	SH4_SKIN	docetaxel	148124	0.066803672
ACH-000092	NCIH2452_PLEURA	docetaxel	148124	0.066861584
ACH-000996	HEC251_ENDOMETRIUM	tanespimycin	6505803	0.066884159
ACH-000502	TCCPAN2_PANCREAS	vinblastine	13342	0.066932741
ACH-000765	WM983B_SKIN	vinblastine	13342	0.066940094
ACH-000027	GOS3_CENTRAL_NERVOUS_SYSTEM	paclitaxel	36314	0.066959066
ACH-000266	SNU213_PANCREAS	colchicine	6167	0.066962323
ACH-000219	A375_SKIN	docetaxel	148124	0.067001162
ACH-000107	CAPAN2_PANCREAS	docetaxel	148124	0.067011967
ACH-000950	LOVO_LARGE_INTESTINE	pralatrexate	148121	0.067022263
ACH-000176	LOUNH91_LUNG	ganetespib	135564985	0.067164045
ACH-000012	HCC827_LUNG	pralatrexate	148121	0.067186384
ACH-000941	HEC1B_ENDOMETRIUM	selinexor	71481097	0.067219702
ACH-000792	BFTC909_KIDNEY	filanesib	44224257	0.067267163
ACH-000018	T24_URINARY_TRACT	filanesib	44224257	0.067270806
ACH-000955	SNU407_LARGE_INTESTINE	pralatrexate	148121	0.067277699
ACH-001239	WM2664_SKIN	vindesine	40839	0.067288681
ACH-000860	NCIH358_LUNG	pralatrexate	148121	0.067376762
ACH-000719	RMGI_OVARY	BNC105	24786555	0.067410634
ACH-000221	SNU398_LIVER	tanespimycin	6505803	0.067424244
ACH-000421	SW837_LARGE_INTESTINE	alvespimycin	5288674	0.067468399
ACH-000650	IGR37_SKIN	tosedostat	15547703	0.06748271

ACH-000808	HUH28_BILIARY_TRACT	NVP-AUY922	135539077	0.067496435
ACH-000232	U251MG_CENTRAL_NERVOUS_SYSTEM	ispinesib	6851740	0.067506227
ACH-000329	CCFSTTG1_CENTRAL_NERVOUS_SYSTEM	filanesib	44224257	0.067523533
ACH-000504	SNB75_CENTRAL_NERVOUS_SYSTEM	elesclomol	300471	0.067535701
ACH-000875	NCIH2347_LUNG	filanesib	44224257	0.067586785
ACH-000777	KYSE30_OESOPHAGUS	tanespimycin	6505803	0.067592405
ACH-000943	RKO_LARGE_INTESTINE	litronesib	25167017	0.067615838
ACH-000648	NCIH28_PLEURA	FK-866	6914657	0.067715712
ACH-000159	OSRC2_KIDNEY	pralatrexate	148121	0.0677194
ACH-000681	A549_LUNG	filanesib	44224257	0.067724131
ACH-000270	HPAC_PANCREAS	alvespimycin	5288674	0.067740003
ACH-000783	CAMA1_BREAST	colchicine	6167	0.067760351
ACH-000783	CAMA1_BREAST	vindesine	40839	0.067809276
ACH-001075	NCIH292_LUNG	pralatrexate	148121	0.067820861
ACH-000504	SNB75_CENTRAL_NERVOUS_SYSTEM	paclitaxel	36314	0.06788828
ACH-000189	RCC10RGB_KIDNEY	docetaxel	148124	0.067914719
ACH-000280	SNU840_OVARY	elesclomol	300471	0.067918375
ACH-000324	JHOC5_OVARY	vincristine	5388993	0.067999462
ACH-000176	LOUNH91_LUNG	alvespimycin	5288674	0.068006329
ACH-000174	CAL62_THYROID	tanespimycin	6505803	0.068025811
ACH-000423	SKMEL3_SKIN	filanesib	44224257	0.068038572
ACH-000570	YKG1_CENTRAL_NERVOUS_SYSTEM	pralatrexate	148121	0.068064294
ACH-000996	HEC251_ENDOMETRIUM	elesclomol	300471	0.068081684
ACH-000898	SNU719_STOMACH	filanesib	44224257	0.068094954
ACH-000719	RMGI_OVARY	BI-2536	11364421	0.068117153
ACH-000599	PATU8902_PANCREAS	pralatrexate	148121	0.068127843
ACH-000882	IGR1_SKIN	paclitaxel	36314	0.068174462
ACH-000681	A549_LUNG	pralatrexate	148121	0.068225965
ACH-000893	NCIH1651_LUNG	docetaxel	148124	0.06829903
ACH-000423	SKMEL3_SKIN	paclitaxel	36314	0.068316948
ACH-000182	SNU869_BILIARY_TRACT	tanespimycin	6505803	0.06833066
ACH-000840	HCC366_LUNG	pralatrexate	148121	0.068497905
ACH-000753	JMSU1_URINARY_TRACT	filanesib	44224257	0.068508031
ACH-000211	DAOY_CENTRAL_NERVOUS_SYSTEM	temsirolimus	129009966	0.068526612
ACH-001239	WM2664_SKIN	cabazitaxel	129009963	0.068593078
ACH-000318	TE10_OESOPHAGUS	pralatrexate	148121	0.068663137
ACH-000352	HCC1428_BREAST	paclitaxel	36314	0.068703179
ACH-000408	TE5_OESOPHAGUS	filanesib	44224257	0.068710684
ACH-000903	FTC133_THYROID	NVP-AUY922	135539077	0.068722353
ACH-000661	WM1799_SKIN	paclitaxel	36314	0.068792955
ACH-000423	SKMEL3_SKIN	tanespimycin	6505803	0.068849421
ACH-000579	UACC257_SKIN	vindesine	40839	0.068864447
ACH-000785	NCIH2126_LUNG	pralatrexate	148121	0.068893269

ACH-000484	VMRCRCW_KIDNEY	FK-866	6914657	0.068899625
ACH-000572	G361_SKIN	danusertib	11442891	0.068950115
ACH-000374	HCC1143_BREAST	ganetespib	135564985	0.069000431
ACH-000352	HCC1428_BREAST	vindesine	40839	0.069015104
ACH-000842	SW480_LARGE_INTESTINE	BNC105	24786555	0.069080166
ACH-000936	EFO27_OVARY	NVP-AUY922	135539077	0.069170402
ACH-000123	COV434_OVARY	litronesib	25167017	0.06917063
ACH-000939	SKUT1_SOFT_TISSUE	temsirolimus	129009966	0.069269704
ACH-000846	FADU_UPPER_AERODIGESTIVE_TRACT	piperazine	4837	0.069285219
ACH-000968	COLO792_SKIN	pralatrexate	148121	0.069297171
ACH-000997	HCT15_LARGE_INTESTINE	pralatrexate	148121	0.069342759
ACH-000890	SW1271_LUNG	temsirolimus	129009966	0.069349026
ACH-000102	GMS10_CENTRAL_NERVOUS_SYSTEM	docetaxel	148124	0.069364652
ACH-000484	VMRCRCW_KIDNEY	AZD8330	16666708	0.069394103
ACH-000191	BHT101_THYROID	paclitaxel	36314	0.069478994
ACH-000408	TE5_OESOPHAGUS	paclitaxel	36314	0.069536334
ACH-000331	ISTMES2_PLEURA	LY2606368	46700756	0.069608889
ACH-000787	LXF289_LUNG	ispinesib	6851740	0.069625754
ACH-000719	RMGI_OVARY	vindesine	40839	0.069648081
ACH-000970	SNUC5_LARGE_INTESTINE	NSC-319726	5921672	0.06967053
ACH-000901	HCC1359_LUNG	pralatrexate	148121	0.069685121
ACH-000985	LS411N_LARGE_INTESTINE	pralatrexate	148121	0.069736231
ACH-000176	LOUNH91_LUNG	NVP-AUY922	135539077	0.069841746
ACH-000223	HCC1937_BREAST	docetaxel	148124	0.069862807
ACH-000495	TUHR4TKB_KIDNEY	alvespimycin	5288674	0.069867704
ACH-000517	SNU410_PANCREAS	NVP-AUY922	135539077	0.069873689
ACH-000880	AGS_STOMACH	ispinesib	6851740	0.069921228
ACH-000791	RERFLCAD1_LUNG	paclitaxel	36314	0.070214203
ACH-000421	SW837_LARGE_INTESTINE	carfilzomib	11556711	0.070286841
ACH-000882	IGR1_SKIN	pralatrexate	148121	0.070347789
ACH-000756	GI1_CENTRAL_NERVOUS_SYSTEM	temsirolimus	129009966	0.070457977
ACH-000404	K029AX_SKIN	NVP-AUY922	135539077	0.070467927
ACH-000810	SKMEL30_SKIN	BAY-87-2243	67377767	0.070497071
ACH-000804	NB1_AUTONOMIC_GANGLIA	elesclomol	300471	0.07068868
ACH-000527	OVISE_OVARY	floxuridine	5702211	0.070714643
ACH-000572	G361_SKIN	ganetespib	135564985	0.07076723
ACH-000420	SNU449_LIVER	alvespimycin	5288674	0.070771974
ACH-000893	NCIH1651_LUNG	alvespimycin	5288674	0.07078752
ACH-000270	HPAC_PANCREAS	ganetespib	135564985	0.070858083
ACH-000250	KMRC20_KIDNEY	NVP-AUY922	135539077	0.070937758
ACH-000890	SW1271_LUNG	tanespimycin	6505803	0.071049009
ACH-000661	WM1799_SKIN	pralatrexate	148121	0.071054621
ACH-000862	KMBC2_URINARY_TRACT	temoporfin	60751	0.071082349

ACH-000804	NB1_AUTONOMIC_GANGLIA	temoporfin	60751	0.071090929
ACH-000822	SKMEL24_SKIN	FK-866	6914657	0.07116737
ACH-000161	CORL105_LUNG	docetaxel	148124	0.071184384
ACH-000415	BICR6_UPPER_AERODIGESTIVE_TRACT	pralatrexate	148121	0.071222678
ACH-000749	DMS273_LUNG	AZD8330	16666708	0.071257572
ACH-000420	SNU449_LIVER	carfilzomib	11556711	0.071266307
ACH-000810	SKMEL30_SKIN	vindesine	40839	0.071295261
ACH-000738	GB1_CENTRAL_NERVOUS_SYSTEM	alvespimycin	5288674	0.071312681
ACH-000384	SW780_URINARY_TRACT	filanesib	44224257	0.07133908
ACH-000027	GOS3_CENTRAL_NERVOUS_SYSTEM	NVP-AUY922	135539077	0.071354896
ACH-000669	SW900_LUNG	ganetespib	135564985	0.071426053
ACH-000800	NCIH446_LUNG	pralatrexate	148121	0.071437111
ACH-000042	PANC0203_PANCREAS	FK-866	6914657	0.071520562
ACH-000231	KALS1_CENTRAL_NERVOUS_SYSTEM	ispinesib	6851740	0.071591158
ACH-000447	NCIH2228_LUNG	dinaciclib	46926350	0.071594871
ACH-000123	COV434_OVARY	tanespimycin	6505803	0.071603001
ACH-000503	BICR16_UPPER_AERODIGESTIVE_TRACT	pralatrexate	148121	0.071650258
ACH-000359	MG63_BONE	tosedostat	15547703	0.071725999
ACH-000014	HS294T_SKIN	ispinesib	6851740	0.071811559
ACH-000404	K029AX_SKIN	filanesib	44224257	0.071832859
ACH-000903	FTC133_THYROID	temsirolimus	129009966	0.071878794
ACH-001321	TT_THYROID	alvespimycin	5288674	0.071885798
ACH-000843	HARA_LUNG	tanespimycin	6505803	0.071886956
ACH-000822	SKMEL24_SKIN	tanespimycin	6505803	0.071895967
ACH-000716	TT2609C02_THYROID	ispinesib	6851740	0.07193466
ACH-000766	NCIH1648_LUNG	temoporfin	60751	0.071955302
ACH-000678	MKN7_STOMACH	tanespimycin	6505803	0.072015834
ACH-000774	RERFLCAD2_LUNG	ingenol-mebutate	6918670	0.072085753
ACH-000720	TCCSUP_URINARY_TRACT	pralatrexate	148121	0.072100896
ACH-000086	ACCMESO1_PLEURA	ingenol-mebutate	6918670	0.072291575
ACH-000376	SF295_CENTRAL_NERVOUS_SYSTEM	filanesib	44224257	0.072323605
ACH-000888	NCIH1793_LUNG	docetaxel	148124	0.072433011
ACH-000562	HCC78_LUNG	ispinesib	6851740	0.072496658
ACH-000579	UACC257_SKIN	docetaxel	148124	0.072545411
ACH-000102	GMS10_CENTRAL_NERVOUS_SYSTEM	pralatrexate	148121	0.072547908
ACH-000352	HCC1428_BREAST	docetaxel	148124	0.07257108
ACH-000471	LI7_LIVER	filanesib	44224257	0.07257311
ACH-000397	TEN_ENDOMETRIUM	FK-866	6914657	0.072613692
ACH-000701	RMUGS_OVARY	FK-866	6914657	0.072629397
ACH-000164	PANC1_PANCREAS	docetaxel	148124	0.072630442
ACH-000324	JHOC5_OVARY	pralatrexate	148121	0.072632576
ACH-000573	MDAMB436_BREAST	pralatrexate	148121	0.072632656
ACH-000903	FTC133_THYROID	paclitaxel	36314	0.072811964

ACH-000771	BICR56_UPPER_AERODIGESTIVE_TRACT	filanesib	44224257	0.072861679
ACH-000924	NCIH2172_LUNG	temsirolimus	129009966	0.0728622
ACH-000027	GOS3_CENTRAL_NERVOUS_SYSTEM	pralatrexate	148121	0.072873378
ACH-000788	A2058_SKIN	temsirolimus	129009966	0.072956947
ACH-000678	MKN7_STOMACH	vindesine	40839	0.073074604
ACH-000359	MG63_BONE	pralatrexate	148121	0.07309216
ACH-000803	COLO668_LUNG	ingenol-mebutate	6918670	0.073147533
ACH-000868	HCC1195_LUNG	NVP-AUY922	135539077	0.073222405
ACH-000868	HCC1195_LUNG	MPI-0479605	46909588	0.073278142
ACH-000019	MCF7_BREAST	bortezomib	387447	0.073304934
ACH-000613	HOS_BONE	elesclomol	300471	0.073316496
ACH-000329	CCFSTTG1_CENTRAL_NERVOUS_SYSTEM	BI-2536	11364421	0.073347124
ACH-000164	PANC1_PANCREAS	vinblastine	13342	0.073430809
ACH-000527	OVISE_OVARY	pralatrexate	148121	0.073449057
ACH-000495	TUHR4TKB_KIDNEY	docetaxel	148124	0.073500276
ACH-000209	SNU1079_BILIARY_TRACT	docetaxel	148124	0.07355723
ACH-000019	MCF7_BREAST	pralatrexate	148121	0.073578117
ACH-000936	EFO27_OVARY	filanesib	44224257	0.073583009
ACH-000648	NCIH28_PLEURA	NVP-AUY922	135539077	0.073659334
ACH-000086	ACCMESO1_PLEURA	pralatrexate	148121	0.07366925
ACH-000347	QGP1_PANCREAS	docetaxel	148124	0.073692751
ACH-000054	HT1080_SOFT_TISSUE	piperazine	4837	0.07372937
ACH-000804	NB1_AUTONOMIC_GANGLIA	piperazine	4837	0.073774744
ACH-000097	ZR751_BREAST	ganetespib	135564985	0.073800349
ACH-000235	PANC0403_PANCREAS	filanesib	44224257	0.073836024
ACH-000296	OUMS23_LARGE_INTESTINE	filanesib	44224257	0.073852144
ACH-000679	OE19_OESOPHAGUS	paclitaxel	36314	0.073901245
ACH-000479	KNS81_CENTRAL_NERVOUS_SYSTEM	dinaciclib	46926350	0.073936179
ACH-000277	HCC1419_BREAST	filanesib	44224257	0.073945957
ACH-000441	SH4_SKIN	paclitaxel	36314	0.073963642
ACH-000652	SUIT2_PANCREAS	FK-866	6914657	0.073983534
ACH-000207	DETROIT562_UPPER_AERODIGESTIVE_TRACT	ingenol-mebutate	6918670	0.074003018
ACH-000684	KMRC1_KIDNEY	alvespimycin	5288674	0.074037505
ACH-000476	JHH4_LIVER	NVP-AUY922	135539077	0.074085769
ACH-000911	NUGC3_STOMACH	poziotinib	25127713	0.074115944
ACH-000228	BICR31_UPPER_AERODIGESTIVE_TRACT	tanespimycin	6505803	0.074122281
ACH-000211	DAOY_CENTRAL_NERVOUS_SYSTEM	tanespimycin	6505803	0.074148986
ACH-000433	CAKI1_KIDNEY	BI-2536	11364421	0.074161398
ACH-000118	HUPT3_PANCREAS	tanespimycin	6505803	0.074196145
ACH-000035	NCIH1650_LUNG	vinflunine	11967282	0.074206267
ACH-000482	RERFLCKJ_LUNG	GSK461364	15983966	0.074219406
ACH-000244	DKMG_CENTRAL_NERVOUS_SYSTEM	filanesib	44224257	0.074228861
ACH-000232	U251MG_CENTRAL_NERVOUS_SYSTEM	rigosertib	6918736	0.07423365

ACH-000945	NCIH650_LUNG	elesclomol	300471	0.074235371
ACH-000609	SF126_CENTRAL_NERVOUS_SYSTEM	paclitaxel	36314	0.074291394
ACH-000270	HPAC_PANCREAS	BI-2536	11364421	0.074295977
ACH-000155	SW1990_PANCREAS	paclitaxel	36314	0.074330921
ACH-000457	CAL54_KIDNEY	NVP-AUY922	135539077	0.074349019
ACH-000517	SNU410_PANCREAS	ganetespib	135564985	0.074373532
ACH-000046	ACHN_KIDNEY	tanespimycin	6505803	0.07440725
ACH-000153	NCIH2052_PLEURA	Ro-4987655	11548630	0.074434432
ACH-000163	SW579_THYROID	7-aminocephalosporanic-acid	441328	0.074475868
ACH-000141	SNU308_BILIARY_TRACT	ganetespib	135564985	0.074484518
ACH-000408	TE5_OESOPHAGUS	tanespimycin	6505803	0.074504415
ACH-000433	CAKI1_KIDNEY	filanesib	44224257	0.074554736
ACH-000532	SNU61_LARGE_INTESTINE	pralatrexate	148121	0.074564503
ACH-000846	FADU_UPPER_AERODIGESTIVE_TRACT	ingenol-mebutate	6918670	0.074672869
ACH-000343	NCIH522_LUNG	ganetespib	135564985	0.074710398
ACH-001239	WM2664_SKIN	docetaxel	148124	0.074729304
ACH-000164	PANC1_PANCREAS	paclitaxel	36314	0.074751205
ACH-000302	SNU1077_ENDOMETRIUM	ganetespib	135564985	0.074752621
ACH-000715	SNU1214_UPPER_AERODIGESTIVE_TRACT	tanespimycin	6505803	0.074756204
ACH-000579	UACC257_SKIN	NVP-AUY922	135539077	0.07479183
ACH-000967	SNUC2A_LARGE_INTESTINE	ganetespib	135564985	0.074827778
ACH-000417	PANC0813_PANCREAS	temsirolimus	129009966	0.074830494
ACH-000974	SNGM_ENDOMETRIUM	tanespimycin	6505803	0.074859819
ACH-000266	SNU213_PANCREAS	FK-866	6914657	0.074871591
ACH-000893	NCIH1651_LUNG	NVP-AUY922	135539077	0.074891469
ACH-000803	COLO668_LUNG	paclitaxel	36314	0.074902633
ACH-000141	SNU308_BILIARY_TRACT	pralatrexate	148121	0.075054073
ACH-000307	PK1_PANCREAS	litronesib	25167017	0.075096833
ACH-000102	GMS10_CENTRAL_NERVOUS_SYSTEM	tanespimycin	6505803	0.075102569
ACH-000082	G292CLONEA141B1_BONE	tosedostat	15547703	0.075206753
ACH-000881	MELJUSO_SKIN	pralatrexate	148121	0.075258985
ACH-000856	CAL51_BREAST	paclitaxel	36314	0.075311667
ACH-000099	SIMA_AUTONOMIC_GANGLIA	NVP-AUY922	135539077	0.075315762
ACH-000893	NCIH1651_LUNG	ganetespib	135564985	0.075358387
ACH-000663	OVTOKO_OVARY	alvespimycin	5288674	0.075373886
ACH-000223	HCC1937_BREAST	filanesib	44224257	0.075445019
ACH-000681	A549_LUNG	paclitaxel	36314	0.075465369
ACH-000558	A172_CENTRAL_NERVOUS_SYSTEM	dinaciclib	46926350	0.075475398
ACH-000322	HT144_SKIN	piperazine	4837	0.07553866
ACH-000888	NCIH1793_LUNG	dinaciclib	46926350	0.075563085
ACH-000500	SNU46_UPPER_AERODIGESTIVE_TRACT	elesclomol	300471	0.075592501
ACH-000885	TOV21G_OVARY	piperazine	4837	0.075601239
ACH-000488	TE11_OESOPHAGUS	dasatinib	3062316	0.075604435

ACH-000652	SUIT2_PANCREAS	NVP-AUY922	135539077	0.075611208
ACH-000250	KMRC20_KIDNEY	ganetespib	135564985	0.07561352
ACH-000565	RCM1_LARGE_INTESTINE	litronesib	25167017	0.075867072
ACH-000209	SNU1079_BILIARY_TRACT	alvespimycin	5288674	0.07587611
ACH-000352	HCC1428_BREAST	NSC-319726	5921672	0.075926426
ACH-000270	HPAC_PANCREAS	raltitrexed	135400182	0.075927291
ACH-000102	GMS10_CENTRAL_NERVOUS_SYSTEM	paclitaxel	36314	0.075930364
ACH-000875	NCIH2347_LUNG	pralatrexate	148121	0.075965707
ACH-000047	GCIY_STOMACH	rigosertib	6918736	0.075972501
ACH-000878	HCC15_LUNG	litronesib	25167017	0.075977564
ACH-000573	MDAMB436_BREAST	ispinesib	6851740	0.076086226
ACH-000527	OVISE_OVARY	paclitaxel	36314	0.076087009
ACH-001239	WM2664_SKIN	BI-2536	11364421	0.076133486
ACH-000288	BT549_BREAST	tanespimycin	6505803	0.076169636
ACH-000007	LS513_LARGE_INTESTINE	alvespimycin	5288674	0.076217422
ACH-000805	COLO679_SKIN	piperazine	4837	0.076268899
ACH-000802	BFTC905_URINARY_TRACT	tanespimycin	6505803	0.076283128
ACH-000332	YAPC_PANCREAS	tanespimycin	6505803	0.076291755
ACH-000161	CORL105_LUNG	filanesib	44224257	0.076411271
ACH-000292	NCIH841_LUNG	tanespimycin	6505803	0.076420813
ACH-000141	SNU308_BILIARY_TRACT	elesclomol	300471	0.076513915
ACH-000420	SNU449_LIVER	docetaxel	148124	0.076637315
ACH-000420	SNU449_LIVER	pralatrexate	148121	0.076641085
ACH-000082	G292CLONEA141B1_BONE	ganetespib	135564985	0.076739002
ACH-000099	SIMA_AUTONOMIC_GANGLIA	filanesib	44224257	0.076740347
ACH-000812	COLO783_SKIN	temsirolimus	129009966	0.076786028
ACH-000329	CCFSTTG1_CENTRAL_NERVOUS_SYSTEM	paclitaxel	36314	0.076848294
ACH-000950	LOVO_LARGE_INTESTINE	paclitaxel	36314	0.076869173
ACH-000359	MG63_BONE	panobinostat	6918837	0.076884947
ACH-000965	RL952_ENDOMETRIUM	altretamine	2123	0.076900729
ACH-000347	QGP1_PANCREAS	paclitaxel	36314	0.076907347
ACH-000022	PATU8988S_PANCREAS	vindesine	40839	0.077090095
ACH-000210	CADOES1_BONE	filanesib	44224257	0.077101418
ACH-000792	BFTC909_KIDNEY	NSC-319726	5921672	0.077120299
ACH-000397	TEN_ENDOMETRIUM	paclitaxel	36314	0.077122427
ACH-000042	PANC0203_PANCREAS	filanesib	44224257	0.077179434
ACH-000361	SKHEP1_LIVER	temsirolimus	129009966	0.077195441
ACH-000209	SNU1079_BILIARY_TRACT	NVP-AUY922	135539077	0.077257601
ACH-000796	MCAS_OVARY	rigosertib	6918736	0.077262683
ACH-000684	KMRC1_KIDNEY	filanesib	44224257	0.077274486
ACH-000292	NCIH841_LUNG	everolimus	6442177	0.077299897
ACH-000027	GOS3_CENTRAL_NERVOUS_SYSTEM	BI-2536	11364421	0.077304441
ACH-000678	MKN7_STOMACH	pralatrexate	148121	0.07732483

ACH-000954	HEC1A_ENDOMETRIUM	elesclomol	300471	0.077422185
ACH-000479	KNS81_CENTRAL_NERVOUS_SYSTEM	temsirolimus	129009966	0.077431713
ACH-000762	YD38_UPPER_AERODIGESTIVE_TRACT	pralatrexate	148121	0.077473941
ACH-000018	T24_URINARY_TRACT	ganetespib	135564985	0.077503924
ACH-000401	COLO800_SKIN	MPI-0479605	46909588	0.077611488
ACH-000209	SNU1079_BILIARY_TRACT	ganetespib	135564985	0.077613232
ACH-000434	NCIH1915_LUNG	MPI-0479605	46909588	0.07767109
ACH-000468	PK45H_PANCREAS	pralatrexate	148121	0.077826457
ACH-000505	RKN_SOFT_TISSUE	ispinesib	6851740	0.077875937
ACH-000244	DKMG_CENTRAL_NERVOUS_SYSTEM	NVP-AUY922	135539077	0.077885307
ACH-000288	BT549_BREAST	everolimus	6442177	0.077925681
ACH-000332	YAPC_PANCREAS	GSK461364	15983966	0.077941616
ACH-000579	UACC257_SKIN	paclitaxel	36314	0.078007663
ACH-000344	SNU668_STOMACH	docetaxel	148124	0.078014578
ACH-000943	RKO_LARGE_INTESTINE	ispinesib	6851740	0.078043807
ACH-000643	HDQP1_BREAST	pralatrexate	148121	0.078058092
ACH-000805	COLO679_SKIN	ispinesib	6851740	0.078115533
ACH-000810	SKMEL30_SKIN	tanespimycin	6505803	0.078163059
ACH-000771	BICR56_UPPER_AERODIGESTIVE_TRACT	paclitaxel	36314	0.078253979
ACH-001075	NCIH292_LUNG	trametinib	11707110	0.078318272
ACH-000479	KNS81_CENTRAL_NERVOUS_SYSTEM	pralatrexate	148121	0.078350583
ACH-000778	HSC3_UPPER_AERODIGESTIVE_TRACT	rigosertib	6918736	0.078372628
ACH-000231	KALS1_CENTRAL_NERVOUS_SYSTEM	tanespimycin	6505803	0.078418307
ACH-000090	PC3_PROSTATE	ispinesib	6851740	0.078434996
ACH-000888	NCIH1793_LUNG	paclitaxel	36314	0.078441314
ACH-000856	CAL51_BREAST	pralatrexate	148121	0.078453539
ACH-000814	HS939T_SKIN	vinblastine	13342	0.078479411
ACH-000476	JHH4_LIVER	pralatrexate	148121	0.078525313
ACH-000018	T24_URINARY_TRACT	NVP-AUY922	135539077	0.07853823
ACH-000991	SNU81_LARGE_INTESTINE	tanespimycin	6505803	0.078693984
ACH-000719	RMGI_OVARY	pralatrexate	148121	0.078739411
ACH-000582	COLO741_SKIN	paclitaxel	36314	0.078747152
ACH-000765	WM983B_SKIN	docetaxel	148124	0.078757431
ACH-000482	RERFLCKJ_LUNG	temsirolimus	129009966	0.078883959
ACH-000826	CAL12T_LUNG	bortezomib	387447	0.078943292
ACH-000042	PANC0203_PANCREAS	pralatrexate	148121	0.079017806
ACH-000449	MESSA_SOFT_TISSUE	temoporfin	60751	0.079076146
ACH-000117	EFM192A_BREAST	elesclomol	300471	0.079093888
ACH-000447	NCIH2228_LUNG	pralatrexate	148121	0.079136755
ACH-000133	HS729_SOFT_TISSUE	temsirolimus	129009966	0.079186576
ACH-000903	FTC133_THYROID	vindesine	40839	0.079199595
ACH-000846	FADU_UPPER_AERODIGESTIVE_TRACT	NVP-BEZ235	11977753	0.079222663
ACH-000176	LOUNH91_LUNG	dinaciclib	46926350	0.079237985

ACH-000022	PATU8988S_PANCREAS	docetaxel	148124	0.079283478
ACH-000842	SW480_LARGE_INTESTINE	filanesib	44224257	0.079295789
ACH-000679	OE19_OESOPHAGUS	ganetespib	135564985	0.079424437
ACH-000946	HEC265_ENDOMETRIUM	everolimus	6442177	0.079457098
ACH-000787	LXF289_LUNG	pralatrexate	148121	0.079470773
ACH-000086	ACCMESO1_PLEURA	paclitaxel	36314	0.079510351
ACH-000868	HCC1195_LUNG	pralatrexate	148121	0.079548729
ACH-000535	BXPC3_PANCREAS	NSC-319726	5921672	0.079553705
ACH-000376	SF295_CENTRAL_NERVOUS_SYSTEM	dinaciclib	46926350	0.079595758
ACH-000535	BXPC3_PANCREAS	ingenol-mebutate	6918670	0.079688632
ACH-000863	DBTRG05MG_CENTRAL_NERVOUS_SYSTEM	NVP-BEZ235	11977753	0.079776544
ACH-000822	SKMEL24_SKIN	dinaciclib	46926350	0.079790343
ACH-000974	SNGM_ENDOMETRIUM	temsirolimus	129009966	0.079807349
ACH-000476	JHH4_LIVER	ganetespib	135564985	0.079832853
ACH-000090	PC3_PROSTATE	tanespimycin	6505803	0.079870069
ACH-000191	BHT101_THYROID	ispinesib	6851740	0.079878247
ACH-000853	NCIH661_LUNG	floxuridine	5702211	0.079895228
ACH-000008	A101D_SKIN	temsirolimus	129009966	0.079906084
ACH-000176	LOUNH91_LUNG	docetaxel	148124	0.079914907
ACH-000493	SNU423_LIVER	tanespimycin	6505803	0.079968209
ACH-000480	HUH7_LIVER	FK-866	6914657	0.080101856
ACH-000480	HUH7_LIVER	JNJ-26481585	11538455	0.08015476
ACH-000447	NCIH2228_LUNG	elesclomol	300471	0.080170477
ACH-000332	YAPC_PANCREAS	pralatrexate	148121	0.080171831
ACH-000102	GMS10_CENTRAL_NERVOUS_SYSTEM	KX2-391	23635314	0.080224296
ACH-000164	PANC1_PANCREAS	tanespimycin	6505803	0.080318719
ACH-000736	SNU601_STOMACH	temsirolimus	129009966	0.080378562
ACH-000781	NCIH2023_LUNG	bortezomib	387447	0.080380072
ACH-000783	CAMA1_BREAST	vinblastine	13342	0.080490501
ACH-000376	SF295_CENTRAL_NERVOUS_SYSTEM	pralatrexate	148121	0.080524885
ACH-000827	WM793_SKIN	MPI-0479605	46909588	0.08056136
ACH-000762	YD38_UPPER_AERODIGESTIVE_TRACT	BAY-87-2243	67377767	0.080574732
ACH-000783	CAMA1_BREAST	bortezomib	387447	0.0806095
ACH-000502	TCCPAN2_PANCREAS	ingenol-mebutate	6918670	0.080610349
ACH-000954	HEC1A_ENDOMETRIUM	litronesib	25167017	0.080629443
ACH-000592	TM31_CENTRAL_NERVOUS_SYSTEM	BNC105	24786555	0.080700482
ACH-000680	SW948_LARGE_INTESTINE	ganetespib	135564985	0.080763085
ACH-000433	CAKI1_KIDNEY	alvespimycin	5288674	0.080769381
ACH-000457	CAL54_KIDNEY	FK-866	6914657	0.080799706
ACH-000235	PANC0403_PANCREAS	paclitaxel	36314	0.080806691
ACH-000624	HCC1806_BREAST	altretamine	2123	0.080905886
ACH-000163	SW579_THYROID	mubritinib	6444692	0.080916886
ACH-000502	TCCPAN2_PANCREAS	BAY-87-2243	67377767	0.080917808

ACH-001075	NCIH292_LUNG	tanespimycin	6505803	0.080934463
ACH-000404	K029AX_SKIN	paclitaxel	36314	0.080942909
ACH-000628	NCIH596_LUNG	elesclomol	300471	0.080977283
ACH-000847	HGC27_STOMACH	panobinostat	6918837	0.081141738
ACH-000421	SW837_LARGE_INTESTINE	docetaxel	148124	0.0811927
ACH-000046	ACHN_KIDNEY	ispinesib	6851740	0.081222329
ACH-000685	L33_PANCREAS	pralatrexate	148121	0.081314904
ACH-000018	T24_URINARY_TRACT	docetaxel	148124	0.081498346
ACH-000322	HT144_SKIN	ispinesib	6851740	0.081501407
ACH-000441	SH4_SKIN	panobinostat	6918837	0.081522013
ACH-000808	HUH28_BILIARY_TRACT	pralatrexate	148121	0.081522654
ACH-000420	SNU449_LIVER	dinaciclib	46926350	0.081646034
ACH-000669	SW900_LUNG	tanespimycin	6505803	0.081658866
ACH-000132	JHOS2_OVARY	pralatrexate	148121	0.08166024
ACH-000542	HEYA8_OVARY	litronesib	25167017	0.081682458
ACH-000182	SNU869_BILIARY_TRACT	trametinib	11707110	0.081702115
ACH-000648	NCIH28_PLEURA	tanespimycin	6505803	0.081775223
ACH-000503	BICR16_UPPER_AERODIGESTIVE_TRACT	poziotinib	25127713	0.08178127
ACH-000433	CAKI1_KIDNEY	cephalomannine	6436208	0.081808041
ACH-000624	HCC1806_BREAST	BAY-87-2243	67377767	0.081859087
ACH-000278	COV362_OVARY	tanespimycin	6505803	0.081886585
ACH-000457	CAL54_KIDNEY	alvespimycin	5288674	0.081887215
ACH-000270	HPAC_PANCREAS	filanesib	44224257	0.081895356
ACH-000835	GCT_SOFT_TISSUE	pralatrexate	148121	0.082031469
ACH-000393	HLF_LIVER	ingenol-mebutate	6918670	0.082054859
ACH-000389	H4_CENTRAL_NERVOUS_SYSTEM	AZD8330	16666708	0.082074322
ACH-000469	YH13_CENTRAL_NERVOUS_SYSTEM	pralatrexate	148121	0.082134266
ACH-000868	HCC1195_LUNG	elesclomol	300471	0.082263653
ACH-000846	FADU_UPPER_AERODIGESTIVE_TRACT	rigosertib	6918736	0.082436803
ACH-000222	ASPC1_PANCREAS	pralatrexate	148121	0.082539485
ACH-000086	ACCMESO1_PLEURA	tanespimycin	6505803	0.082565537
ACH-000900	NCIH23_LUNG	temsirolimus	129009966	0.082594414
ACH-000163	SW579_THYROID	elesclomol	300471	0.082697982
ACH-000826	CAL12T_LUNG	tanespimycin	6505803	0.082783514
ACH-000808	HUH28_BILIARY_TRACT	temsirolimus	129009966	0.082883026
ACH-000441	SH4_SKIN	tanespimycin	6505803	0.082934222
ACH-000957	LS180_LARGE_INTESTINE	filanesib	44224257	0.082943046
ACH-000885	TOV21G_OVARY	temoporfin	60751	0.083075737
ACH-000840	HCC366_LUNG	tanespimycin	6505803	0.083107817
ACH-000662	CORL23_LUNG	rigosertib	6918736	0.083115491
ACH-000476	JHH4_LIVER	alvespimycin	5288674	0.08323531
ACH-000153	NCIH2052_PLEURA	pralatrexate	148121	0.083240412
ACH-000593	BC3C_URINARY_TRACT	pralatrexate	148121	0.083241576

ACH-000777	KYSE30_OESOPHAGUS	litronesib	25167017	0.083285525
ACH-000376	SF295_CENTRAL_NERVOUS_SYSTEM	temsirolimus	129009966	0.083340178
ACH-000221	SNU398_LIVER	irinotecan	60838	0.083398223
ACH-000990	HEC108_ENDOMETRIUM	tanespimycin	6505803	0.083401362
ACH-000890	SW1271_LUNG	ispinesib	6851740	0.08359581
ACH-000897	FTC238_THYROID	elesclomol	300471	0.083606722
ACH-000311	NCIH2122_LUNG	everolimus	6442177	0.083665182
ACH-000927	BT474_BREAST	FK-866	6914657	0.083771409
ACH-000117	EFM192A_BREAST	tosedostat	15547703	0.083803981
ACH-000775	NCIH727_LUNG	epothilone-b	129010071	0.083874685
ACH-000242	RT4_URINARY_TRACT	SNS-314	24995524	0.083922635
ACH-000545	VMCUB1_URINARY_TRACT	ingenol-mebutate	6918670	0.083969465
ACH-000161	CORL105_LUNG	tanespimycin	6505803	0.084014049
ACH-000842	SW480_LARGE_INTESTINE	tanespimycin	6505803	0.084112826
ACH-000384	SW780_URINARY_TRACT	elesclomol	300471	0.084198181
ACH-000232	U251MG_CENTRAL_NERVOUS_SYSTEM	temsirolimus	129009966	0.084297148
ACH-000738	GB1_CENTRAL_NERVOUS_SYSTEM	docetaxel	148124	0.084302434
ACH-000677	SW1573_LUNG	litronesib	25167017	0.084396092
ACH-000133	HS729_SOFT_TISSUE	elesclomol	300471	0.084440651
ACH-000677	SW1573_LUNG	tanespimycin	6505803	0.084458535
ACH-000019	MCF7_BREAST	tanespimycin	6505803	0.084594449
ACH-000587	NCIH1975_LUNG	idasanutlin	53358942	0.084610743
ACH-000791	RERFLCAD1_LUNG	rigosertib	6918736	0.084618922
ACH-000901	HCC1359_LUNG	tanespimycin	6505803	0.08461976
ACH-000273	SF539_CENTRAL_NERVOUS_SYSTEM	elesclomol	300471	0.084727744
ACH-000277	HCC1419_BREAST	BNC105	24786555	0.084757524
ACH-000967	SNUC2A_LARGE_INTESTINE	pralatrexate	148121	0.084805492
ACH-000444	LU99_LUNG	litronesib	25167017	0.084850967
ACH-000404	K029AX_SKIN	ganetespib	135564985	0.084967058
ACH-000774	RERFLCAD2_LUNG	plinabulin	9949641	0.084985268
ACH-000502	TCCPAN2_PANCREAS	filanesib	44224257	0.085023777
ACH-000027	GOS3_CENTRAL_NERVOUS_SYSTEM	dinaciclib	46926350	0.085049644
ACH-000968	COLO792_SKIN	LY2606368	46700756	0.085076629
ACH-000163	SW579_THYROID	alvocidib	5287969	0.085147518
ACH-000331	ISTMES2_PLEURA	bortezomib	387447	0.085191656
ACH-000549	SNU1076_UPPER_AERODIGESTIVE_TRACT	pralatrexate	148121	0.085324813
ACH-000903	FTC133_THYROID	dinaciclib	46926350	0.085398043
ACH-000332	YAPC_PANCREAS	litronesib	25167017	0.085479281
ACH-000457	CAL54_KIDNEY	ganetespib	135564985	0.085608219
ACH-000368	SNU1105_CENTRAL_NERVOUS_SYSTEM	piperazine	4837	0.085613998
ACH-000836	YD15_SALIVARY_GLAND	pralatrexate	148121	0.085683745
ACH-000027	GOS3_CENTRAL_NERVOUS_SYSTEM	ganetespib	135564985	0.08570982
ACH-000842	SW480_LARGE_INTESTINE	pralatrexate	148121	0.085726424

ACH-000189	RCC10RGB_KIDNEY	elesclomol	300471	0.085897527
ACH-000389	H4_CENTRAL_NERVOUS_SYSTEM	GSK461364	15983966	0.085979774
ACH-000558	A172_CENTRAL_NERVOUS_SYSTEM	filanesib	44224257	0.085983909
ACH-000361	SKHEP1_LIVER	piperazine	4837	0.086036553
ACH-000950	LOVO_LARGE_INTESTINE	trametinib	11707110	0.086105461
ACH-000277	HCC1419_BREAST	paclitaxel	36314	0.08619552
ACH-000407	SNU685_ENDOMETRIUM	tanespimycin	6505803	0.086250971
ACH-000625	HEP3B217_LIVER	filanesib	44224257	0.086329459
ACH-000161	CORL105_LUNG	ingenol-mebutate	6918670	0.086330653
ACH-000903	FTC133_THYROID	everolimus	6442177	0.086368054
ACH-000092	NCIH2452_PLEURA	pralatrexate	148121	0.086382607
ACH-000868	HCC1195_LUNG	ganetespib	135564985	0.086436059
ACH-000086	ACCMESO1_PLEURA	temsirolimus	129009966	0.086440712
ACH-001239	WM2664_SKIN	filanesib	44224257	0.086453279
ACH-000296	OUMS23_LARGE_INTESTINE	tanespimycin	6505803	0.086475456
ACH-000505	RKN_SOFT_TISSUE	tanespimycin	6505803	0.086491203
ACH-000756	GI1_CENTRAL_NERVOUS_SYSTEM	everolimus	6442177	0.086542532
ACH-000441	SH4_SKIN	filanesib	44224257	0.086588463
ACH-000270	HPAC_PANCREAS	vindesine	40839	0.086617699
ACH-000979	DU145_PROSTATE	temsirolimus	129009966	0.086779137
ACH-000191	BHT101_THYROID	idasanutlin	53358942	0.086842545
ACH-000376	SF295_CENTRAL_NERVOUS_SYSTEM	BI-2536	11364421	0.086893093
ACH-000495	TUHR4TKB_KIDNEY	pralatrexate	148121	0.086960616
ACH-000090	PC3_PROSTATE	temsirolimus	129009966	0.086973307
ACH-000477	MALME3M_SKIN	alvespimycin	5288674	0.087061332
ACH-000810	SKMEL30_SKIN	BNC105	24786555	0.087076076
ACH-000834	UMUC1_URINARY_TRACT	ingenol-mebutate	6918670	0.087097694
ACH-000810	SKMEL30_SKIN	docetaxel	148124	0.087100925
ACH-000425	UACC62_SKIN	tanespimycin	6505803	0.087113725
ACH-000054	HT1080_SOFT_TISSUE	tanespimycin	6505803	0.08714031
ACH-000082	G292CLONEA141B1_BONE	BI-2536	11364421	0.087189805
ACH-000680	SW948_LARGE_INTESTINE	NVP-AUY922	135539077	0.08720155
ACH-000632	HS944T_SKIN	idasanutlin	53358942	0.087212562
ACH-000625	HEP3B217_LIVER	KX2-391	23635314	0.08732483
ACH-000903	FTC133_THYROID	ganetespib	135564985	0.087383074
ACH-000309	SKLU1_LUNG	docetaxel	148124	0.087385663
ACH-000579	UACC257_SKIN	vinblastine	13342	0.08752271
ACH-000407	SNU685_ENDOMETRIUM	piperazine	4837	0.087558378
ACH-000766	NCIH1648_LUNG	idasanutlin	53358942	0.08757044
ACH-000496	NCIH1792_LUNG	elesclomol	300471	0.087619877
ACH-000351	MKN1_STOMACH	tanespimycin	6505803	0.087699604
ACH-000266	SNU213_PANCREAS	crystal-violet	3468	0.08772674
ACH-000627	LCLC103H_LUNG	tanespimycin	6505803	0.087738922

ACH-000502	TCCPAN2_PANCREAS	paclitaxel	36314	0.087752567
ACH-000479	KNS81_CENTRAL_NERVOUS_SYSTEM	ispinesib	6851740	0.087790461
ACH-000505	RKN_SOFT_TISSUE	altretamine	2123	0.087846048
ACH-000159	OSRC2_KIDNEY	filanesib	44224257	0.087874859
ACH-000679	OE19_OESOPHAGUS	pralatrexate	148121	0.08790111
ACH-000957	LS180_LARGE_INTESTINE	docetaxel	148124	0.087919423
ACH-000652	SUIT2_PANCREAS	bortezomib	387447	0.087956594
ACH-000822	SKMEL24_SKIN	BNC105	24786555	0.088026064
ACH-000098	GAMG_CENTRAL_NERVOUS_SYSTEM	elesclomol	300471	0.088185588
ACH-000232	U251MG_CENTRAL_NERVOUS_SYSTEM	litronesib	25167017	0.088213062
ACH-000423	SKMEL3_SKIN	pralatrexate	148121	0.088309073
ACH-000749	DMS273_LUNG	tanespimycin	6505803	0.088373439
ACH-000974	SNGM_ENDOMETRIUM	ingenol-mebutate	6918670	0.088502054
ACH-000307	PK1_PANCREAS	temoporfin	60751	0.088504043
ACH-000609	SF126_CENTRAL_NERVOUS_SYSTEM	temsirolimus	129009966	0.088529203
ACH-000461	SNU1196_BILIARY_TRACT	pralatrexate	148121	0.088605948
ACH-000416	NCIH838_LUNG	temsirolimus	129009966	0.088656484
ACH-000950	LOVO_LARGE_INTESTINE	ingenol-mebutate	6918670	0.088721933
ACH-000572	G361_SKIN	tanespimycin	6505803	0.088743723
ACH-000164	PANC1_PANCREAS	filanesib	44224257	0.088755342
ACH-000223	HCC1937_BREAST	paclitaxel	36314	0.088776561
ACH-000495	TUHR4TKB_KIDNEY	MLN0128	45375953	0.088787005
ACH-000545	VMCUB1_URINARY_TRACT	BAY-87-2243	67377767	0.08882843
ACH-000563	EBC1_LUNG	piperazine	4837	0.088890395
ACH-000374	HCC1143_BREAST	pralatrexate	148121	0.088904967
ACH-000035	NCIH1650_LUNG	pralatrexate	148121	0.088908146
ACH-000237	JHOM1_OVARY	temoporfin	60751	0.08893892
ACH-000950	LOVO_LARGE_INTESTINE	panobinostat	6918837	0.088969921
ACH-000153	NCIH2052_PLEURA	trametinib	11707110	0.089107128
ACH-000428	UO31_KIDNEY	alvespimycin	5288674	0.089198617
ACH-000997	HCT15_LARGE_INTESTINE	elesclomol	300471	0.089275921
ACH-000968	COLO792_SKIN	tanespimycin	6505803	0.089306753
ACH-000570	YKG1_CENTRAL_NERVOUS_SYSTEM	temsirolimus	129009966	0.089377724
ACH-000505	RKN_SOFT_TISSUE	piperazine	4837	0.089378265
ACH-000778	HSC3_UPPER_AERODIGESTIVE_TRACT	ispinesib	6851740	0.089388239
ACH-001318	PLCPRF5_LIVER	altretamine	2123	0.089393669
ACH-000665	SKMES1_LUNG	elesclomol	300471	0.089480276
ACH-000853	NCIH661_LUNG	temsirolimus	129009966	0.089629313
ACH-000726	TE14_OESOPHAGUS	ingenol-mebutate	6918670	0.089645838
ACH-000624	HCC1806_BREAST	pralatrexate	148121	0.089646522
ACH-000542	HEYA8_OVARY	ispinesib	6851740	0.089660908
ACH-000846	FADU_UPPER_AERODIGESTIVE_TRACT	elesclomol	300471	0.08967772
ACH-000895	CL34_LARGE_INTESTINE	piperazine	4837	0.089687756

ACH-000549	SNU1076_UPPER_AERODIGESTIVE_TRACT	BNC105	24786555	0.089732521
ACH-000447	NCIH2228_LUNG	dasatinib	3062316	0.089780221
ACH-000535	BXPC3_PANCREAS	vindesine	40839	0.089858482
ACH-000885	TOV21G_OVARY	everolimus	6442177	0.089878517
ACH-000677	SW1573_LUNG	ispinesib	6851740	0.089894148
ACH-000903	FTC133_THYROID	vinblastine	13342	0.089897779
ACH-000895	CL34_LARGE_INTESTINE	tosedostat	15547703	0.089959055
ACH-000244	DKMG_CENTRAL_NERVOUS_SYSTEM	alvespimycin	5288674	0.089973003
ACH-000783	CAMA1_BREAST	BAY-87-2243	67377767	0.09005318
ACH-000628	NCIH596_LUNG	tanespimycin	6505803	0.09007076
ACH-000955	SNU407_LARGE_INTESTINE	tanespimycin	6505803	0.090102892
ACH-000990	HEC108_ENDOMETRIUM	temsirolimus	129009966	0.090126161
ACH-000309	SKLU1_LUNG	ingenol-mebutate	6918670	0.090169317
ACH-000913	ESS1_ENDOMETRIUM	pralatrexate	148121	0.090210054
ACH-000888	NCIH1793_LUNG	bortezomib	387447	0.090236882
ACH-000895	CL34_LARGE_INTESTINE	litronesib	25167017	0.090240789
ACH-000480	HUH7_LIVER	alvespimycin	5288674	0.090471799
ACH-000879	MFE296_ENDOMETRIUM	tanespimycin	6505803	0.09047787
ACH-000781	NCIH2023_LUNG	vindesine	40839	0.090507986
ACH-000895	CL34_LARGE_INTESTINE	idasanutlin	53358942	0.090543832
ACH-000463	NCIH460_LUNG	temoporfin	60751	0.090587721
ACH-000007	LS513_LARGE_INTESTINE	pralatrexate	148121	0.090689758
ACH-000517	SNU410_PANCREAS	tanespimycin	6505803	0.090736399
ACH-000153	NCIH2052_PLEURA	PD-0325901	9826528	0.090750882
ACH-000223	HCC1937_BREAST	vindesine	40839	0.090798674
ACH-000347	QGP1_PANCREAS	NSC-319726	5921672	0.09082981
ACH-000484	VMRCRCW_KIDNEY	NVP-AUY922	135539077	0.090884322
ACH-000302	SNU1077_ENDOMETRIUM	NVP-AUY922	135539077	0.090900142
ACH-000704	OAW42_OVARY	paclitaxel	36314	0.090933735
ACH-000155	SW1990_PANCREAS	ispinesib	6851740	0.091014746
ACH-000331	ISTMES2_PLEURA	filanesib	44224257	0.09112739
ACH-000209	SNU1079_BILIARY_TRACT	elesclomol	300471	0.091171648
ACH-001321	TT_THYROID	dinaciclib	46926350	0.091245987
ACH-000759	MDAMB175VII_BREAST	LY2606368	46700756	0.091305168
ACH-000221	SNU398_LIVER	temoporfin	60751	0.091349463
ACH-000677	SW1573_LUNG	pralatrexate	148121	0.091365422
ACH-000018	T24_URINARY_TRACT	BI-2536	11364421	0.091398425
ACH-000393	HLF_LIVER	ispinesib	6851740	0.091454392
ACH-001321	TT_THYROID	FK-866	6914657	0.091473597
ACH-000142	CAL29_URINARY_TRACT	rigosertib	6918736	0.091473618
ACH-000107	CAPAN2_PANCREAS	pralatrexate	148121	0.091478715
ACH-000066	HCC4006_LUNG	elesclomol	300471	0.091512593
ACH-000804	NB1_AUTONOMIC_GANGLIA	altretamine	2123	0.091572019

ACH-000404	K029AX_SKIN	temsirolimus	129009966	0.091605076
ACH-000117	EFM192A_BREAST	JNJ-26481585	11538455	0.09161221
ACH-000416	NCIH838_LUNG	PF-477736	135565545	0.091619848
ACH-000785	NCIH2126_LUNG	elesclomol	300471	0.091666442
ACH-000178	HS766T_PANCREAS	pralatrexate	148121	0.091667119
ACH-000899	WM88_SKIN	ganetespib	135564985	0.091677398
ACH-000748	SJSA1_BONE	paclitaxel	36314	0.091721066
ACH-000882	IGR1_SKIN	trametinib	11707110	0.091756347
ACH-000035	NCIH1650_LUNG	AZD8330	16666708	0.091758986
ACH-000991	SNU81_LARGE_INTESTINE	temsirolimus	129009966	0.091788821
ACH-000715	SNU1214_UPPER_AERODIGESTIVE_TRACT	pralatrexate	148121	0.091805975
ACH-000939	SKUT1_SOFT_TISSUE	everolimus	6442177	0.091884117
ACH-000972	HEC151_ENDOMETRIUM	tanespimycin	6505803	0.091884391
ACH-000425	UACC62_SKIN	AS-703026	44187362	0.091887351
ACH-000212	CAL120_BREAST	temsirolimus	129009966	0.091953415
ACH-000833	RH30_SOFT_TISSUE	KX2-391	23635314	0.091993339
ACH-000138	CFPAC1_PANCREAS	tanespimycin	6505803	0.091998638
ACH-000593	BC3C_URINARY_TRACT	BNC105	24786555	0.092060933
ACH-000164	PANC1_PANCREAS	AZD8330	16666708	0.092077827
ACH-000155	SW1990_PANCREAS	temsirolimus	129009966	0.092117914
ACH-000099	SIMA_AUTONOMIC_GANGLIA	pralatrexate	148121	0.092160647
ACH-000211	DAOY_CENTRAL_NERVOUS_SYSTEM	MLN0128	45375953	0.092190489
ACH-000579	UACC257_SKIN	filanesib	44224257	0.092196763
ACH-000176	LOUNH91_LUNG	trametinib	11707110	0.092217399
ACH-000847	HGC27_STOMACH	idasanutlin	53358942	0.092247738
ACH-000060	PANC1005_PANCREAS	elesclomol	300471	0.0922631
ACH-000324	JHOC5_OVARY	tanespimycin	6505803	0.092282395
ACH-000066	HCC4006_LUNG	pralatrexate	148121	0.092283483
ACH-000517	SNU410_PANCREAS	bortezomib	387447	0.092302825
ACH-000955	SNU407_LARGE_INTESTINE	temsirolimus	129009966	0.092307373
ACH-000496	NCIH1792_LUNG	ingenol-mebutate	6918670	0.092325886
ACH-000305	ECGI10_OESOPHAGUS	tanespimycin	6505803	0.092496423
ACH-000846	FADU_UPPER_AERODIGESTIVE_TRACT	temsirolimus	129009966	0.092517722
ACH-000107	CAPAN2_PANCREAS	BI-2536	11364421	0.092528403
ACH-000721	HMC18_BREAST	idasanutlin	53358942	0.092530722
ACH-000311	NCIH2122_LUNG	talazoparib	135565082	0.092544909
ACH-000890	SW1271_LUNG	everolimus	6442177	0.092608389
ACH-000396	J82_URINARY_TRACT	ispinesib	6851740	0.092701367
ACH-000329	CCFSTTG1_CENTRAL_NERVOUS_SYSTEM	pralatrexate	148121	0.092781751
ACH-000421	SW837_LARGE_INTESTINE	paclitaxel	36314	0.092786184
ACH-000764	SH10TC_STOMACH	temoporfin	60751	0.092820044
ACH-000954	HEC1A_ENDOMETRIUM	temsirolimus	129009966	0.092903882
ACH-000161	CORL105_LUNG	pralatrexate	148121	0.09295581

ACH-000890	SW1271_LUNG	litronesib	25167017	0.092974139
ACH-000324	JHOC5_OVARY	ispinesib	6851740	0.093014865
ACH-000117	EFM192A_BREAST	tanespimycin	6505803	0.093091161
ACH-000403	NCIH747_LARGE_INTESTINE	BAY-87-2243	67377767	0.093101132
ACH-000781	NCIH2023_LUNG	filanesib	44224257	0.093111983
ACH-000648	NCIH28_PLEURA	filanesib	44224257	0.093134968
ACH-000888	NCIH1793_LUNG	pralatrexate	148121	0.093177777
ACH-000936	EFO27_OVARY	paclitaxel	36314	0.093279712
ACH-000311	NCIH2122_LUNG	temoporfin	60751	0.093328844
ACH-000573	MDAMB436_BREAST	tanespimycin	6505803	0.093351098
ACH-000311	NCIH2122_LUNG	dasatinib	3062316	0.093358595
ACH-000777	KYSE30_OESOPHAGUS	ispinesib	6851740	0.09338953
ACH-000856	CAL51_BREAST	tanespimycin	6505803	0.093473111
ACH-000415	BICR6_UPPER_AERODIGESTIVE_TRACT	ispinesib	6851740	0.093480993
ACH-000893	NCIH1651_LUNG	pralatrexate	148121	0.093491527
ACH-000221	SNU398_LIVER	panobinostat	6918837	0.093536419
ACH-000389	H4_CENTRAL_NERVOUS_SYSTEM	pralatrexate	148121	0.09354241
ACH-000098	GAMG_CENTRAL_NERVOUS_SYSTEM	pralatrexate	148121	0.093576494
ACH-000652	SUIT2_PANCREAS	elesclomol	300471	0.093579979
ACH-000164	PANC1_PANCREAS	NVP-AUY922	135539077	0.093599299
ACH-000374	HCC1143_BREAST	tanespimycin	6505803	0.093660397
ACH-000176	LOUNH91_LUNG	tanespimycin	6505803	0.093793464
ACH-000813	T3M10_LUNG	temsirolimus	129009966	0.093848368
ACH-000630	YD8_UPPER_AERODIGESTIVE_TRACT	alvespimycin	5288674	0.093866637
ACH-000434	NCIH1915_LUNG	pralatrexate	148121	0.093886178
ACH-000735	PECAPJ49_UPPER_AERODIGESTIVE_TRACT	temoporfin	60751	0.093909768
ACH-000810	SKMEL30_SKIN	filanesib	44224257	0.093945196
ACH-000384	SW780_URINARY_TRACT	paclitaxel	36314	0.093962111
ACH-000404	K029AX_SKIN	pralatrexate	148121	0.094051104
ACH-000990	HEC108_ENDOMETRIUM	paclitaxel	36314	0.094060646
ACH-000096	G401_SOFT_TISSUE	temoporfin	60751	0.094077005
ACH-000572	G361_SKIN	pralatrexate	148121	0.094239918
ACH-000909	JHUEM2_ENDOMETRIUM	piperazine	4837	0.09424762
ACH-000535	BXPC3_PANCREAS	JNJ-26481585	11538455	0.094302516
ACH-000261	RERFLCAI_LUNG	NVP-BEZ235	11977753	0.094339916
ACH-000288	BT549_BREAST	panobinostat	6918837	0.094360572
ACH-000361	SKHEP1_LIVER	tanespimycin	6505803	0.094407751
ACH-000558	A172_CENTRAL_NERVOUS_SYSTEM	docetaxel	148124	0.094430759
ACH-000502	TCCPAN2_PANCREAS	docetaxel	148124	0.094439664
ACH-000313	KMRC3_KIDNEY	tanespimycin	6505803	0.094480564
ACH-000758	MKN74_STOMACH	alvespimycin	5288674	0.094486927
ACH-000955	SNU407_LARGE_INTESTINE	panobinostat	6918837	0.09448859
ACH-000324	JHOC5_OVARY	litronesib	25167017	0.094491278

ACH-000393	HLF_LIVER	tosedostat	15547703	0.094521793
ACH-000141	SNU308_BILIARY_TRACT	BI-2536	11364421	0.094532935
ACH-000671	HUH6_LIVER	piperazine	4837	0.094584756
ACH-000351	MKN1_STOMACH	litronesib	25167017	0.094588725
ACH-000669	SW900_LUNG	ingenol-mebutate	6918670	0.094589335
ACH-001239	WM2664_SKIN	paclitaxel	36314	0.094638762
ACH-000480	HUH7_LIVER	NVP-AUY922	135539077	0.094679339
ACH-000759	MDAMB175VII_BREAST	colchicine	6167	0.094682256
ACH-000456	BCPAP_THYROID	BAY-87-2243	67377767	0.094686087
ACH-000008	A101D_SKIN	tanespimycin	6505803	0.094709446
ACH-000408	TE5_OESOPHAGUS	ingenol-mebutate	6918670	0.094710362
ACH-000311	NCIH2122_LUNG	piperazine	4837	0.094794551
ACH-000808	HUH28_BILIARY_TRACT	ganetespib	135564985	0.094803289
ACH-000444	LU99_LUNG	temsirolimus	129009966	0.094803751
ACH-000719	RMGI_OVARY	tosedostat	15547703	0.094816737
ACH-000500	SNU46_UPPER_AERODIGESTIVE_TRACT	temsirolimus	129009966	0.094823562
ACH-000359	MG63_BONE	mubritinib	6444692	0.09488953
ACH-000117	EFM192A_BREAST	ganetespib	135564985	0.094922665
ACH-000008	A101D_SKIN	ispinesib	6851740	0.094941621
ACH-000133	HS729_SOFT_TISSUE	piperazine	4837	0.094965006
ACH-000428	UO31_KIDNEY	pralatrexate	148121	0.094968737
ACH-000496	NCIH1792_LUNG	SNS-314	24995524	0.094977902
ACH-000484	VMRCRCW_KIDNEY	alvespimycin	5288674	0.094979984
ACH-000788	A2058_SKIN	everolimus	6442177	0.09503317
ACH-000652	SUIT2_PANCREAS	filanesib	44224257	0.095125458
ACH-000652	SUIT2_PANCREAS	dinaciclib	46926350	0.09512871
ACH-000364	U2OS_BONE	elesclomol	300471	0.095164523
ACH-000758	MKN74_STOMACH	epothilone-b	129010071	0.095245172
ACH-000504	SNB75_CENTRAL_NERVOUS_SYSTEM	ganetespib	135564985	0.095252208
ACH-000849	MDAMB468_BREAST	tanespimycin	6505803	0.095302191
ACH-000235	PANC0403_PANCREAS	KX2-391	23635314	0.095535932
ACH-000811	SKOV3_OVARY	ispinesib	6851740	0.09556873
ACH-000023	PATU8988T_PANCREAS	temoporfin	60751	0.095625462
ACH-000587	NCIH1975_LUNG	temoporfin	60751	0.095701139
ACH-000417	PANC0813_PANCREAS	everolimus	6442177	0.095710089
ACH-000344	SNU668_STOMACH	filanesib	44224257	0.095772902
ACH-000563	EBC1_LUNG	altretamine	2123	0.095855347
ACH-000841	NCIH2087_LUNG	ispinesib	6851740	0.095860824
ACH-000822	SKMEL24_SKIN	ingenol-mebutate	6918670	0.095942349
ACH-000950	LOVO_LARGE_INTESTINE	altretamine	2123	0.095965249
ACH-000316	SNU886_LIVER	tanespimycin	6505803	0.095988818
ACH-000178	HS766T_PANCREAS	tanespimycin	6505803	0.096024827
ACH-000579	UACC257_SKIN	pralatrexate	148121	0.096111159

ACH-000012	HCC827_LUNG	litronesib	25167017	0.09611338
ACH-000332	YAPC_PANCREAS	ingenol-mebutate	6918670	0.096114822
ACH-000941	HEC1B_ENDOMETRIUM	docetaxel	148124	0.096134537
ACH-000244	DKMG_CENTRAL_NERVOUS_SYSTEM	pralatrexate	148121	0.096176541
ACH-000243	DANG_PANCREAS	everolimus	6442177	0.09624854
ACH-000273	SF539_CENTRAL_NERVOUS_SYSTEM	temsirolimus	129009966	0.096348413
ACH-000496	NCIH1792_LUNG	temsirolimus	129009966	0.096369668
ACH-000102	GMS10_CENTRAL_NERVOUS_SYSTEM	vincristine	5388993	0.096432498
ACH-000022	PATU8988S_PANCREAS	paclitaxel	36314	0.096437075
ACH-000219	A375_SKIN	paclitaxel	36314	0.09648072
ACH-000736	SNU601_STOMACH	everolimus	6442177	0.096692129
ACH-000288	BT549_BREAST	piperazine	4837	0.096702205
ACH-000768	MDAMB231_BREAST	temsirolimus	129009966	0.096703725
ACH-000375	G402_SOFT_TISSUE	rigosertib	6918736	0.096864199
ACH-000783	CAMA1_BREAST	filanesib	44224257	0.096908342
ACH-000994	HEC59_ENDOMETRIUM	everolimus	6442177	0.096925356
ACH-000232	U251MG_CENTRAL_NERVOUS_SYSTEM	volasertib	10461508	0.096949443
ACH-000979	DU145_PROSTATE	pralatrexate	148121	0.096958471
ACH-000235	PANC0403_PANCREAS	pralatrexate	148121	0.097031696
ACH-000479	KNS81_CENTRAL_NERVOUS_SYSTEM	everolimus	6442177	0.09704244
ACH-000244	DKMG_CENTRAL_NERVOUS_SYSTEM	ganetespib	135564985	0.097333454
ACH-000856	CAL51_BREAST	AZD8330	16666708	0.09741154
ACH-000678	MKN7_STOMACH	temsirolimus	129009966	0.097432332
ACH-000118	HUPT3_PANCREAS	litronesib	25167017	0.097441268
ACH-000452	TE8_OESOPHAGUS	BI-2536	11364421	0.097465207
ACH-000008	A101D_SKIN	elesclomol	300471	0.097481583
ACH-000749	DMS273_LUNG	temoporfin	60751	0.097534208
ACH-000397	TEN_ENDOMETRIUM	tanespimycin	6505803	0.097685589
ACH-000811	SKOV3_OVARY	everolimus	6442177	0.097752411
ACH-000159	OSRC2_KIDNEY	temsirolimus	129009966	0.097772332
ACH-000384	SW780_URINARY_TRACT	docetaxel	148124	0.097796659
ACH-000810	SKMEL30_SKIN	paclitaxel	36314	0.097808064
ACH-000558	A172_CENTRAL_NERVOUS_SYSTEM	paclitaxel	36314	0.097827158
ACH-000309	SKLU1_LUNG	paclitaxel	36314	0.097862357
ACH-000599	PATU8902_PANCREAS	tanespimycin	6505803	0.097915449
ACH-000250	KMRC20_KIDNEY	alvespimycin	5288674	0.097920035
ACH-000652	SUIT2_PANCREAS	alvespimycin	5288674	0.097932917
ACH-000097	ZR751_BREAST	ispinesib	6851740	0.097949357
ACH-000880	AGS_STOMACH	temoporfin	60751	0.097955976
ACH-000219	A375_SKIN	ispinesib	6851740	0.097958578
ACH-000701	RMUGS_OVARY	filanesib	44224257	0.097980134
ACH-000802	BFTC905_URINARY_TRACT	KX2-391	23635314	0.098031287
ACH-000376	SF295_CENTRAL_NERVOUS_SYSTEM	vindesine	40839	0.098046541

ACH-000117	EFM192A_BREAST	BNC105	24786555	0.09808044
ACH-000427	NCIN87_STOMACH	tanespimycin	6505803	0.098154944
ACH-000853	NCIH661_LUNG	rigosertib	6918736	0.098324749
ACH-000352	HCC1428_BREAST	NVP-AUY922	135539077	0.098378842
ACH-000878	HCC15_LUNG	temoporfin	60751	0.098431193
ACH-000878	HCC15_LUNG	temsirolimus	129009966	0.098433769
ACH-000579	UACC257_SKIN	BNC105	24786555	0.09845594
ACH-000862	KMBC2_URINARY_TRACT	rigosertib	6918736	0.098503394
ACH-000632	HS944T_SKIN	NVP-BEZ235	11977753	0.098577127
ACH-000344	SNU668_STOMACH	raltitrexed	135400182	0.098621585
ACH-000834	UMUC1_URINARY_TRACT	altretamine	2123	0.098628615
ACH-000480	HUH7_LIVER	selinexor	71481097	0.098646582
ACH-000758	MKN74_STOMACH	plinabulin	9949641	0.098694088
ACH-000888	NCIH1793_LUNG	ganetespib	135564985	0.098731392
ACH-000893	NCIH1651_LUNG	paclitaxel	36314	0.098752939
ACH-000447	NCIH2228_LUNG	tanespimycin	6505803	0.098758535
ACH-000762	YD38_UPPER_AERODIGESTIVE_TRACT	ingenol-mebutate	6918670	0.098760747
ACH-000396	J82_URINARY_TRACT	litronesib	25167017	0.098774818
ACH-000785	NCIH2126_LUNG	GSK461364	15983966	0.098846509
ACH-000868	HCC1195_LUNG	litronesib	25167017	0.098924899
ACH-000595	LN229_CENTRAL_NERVOUS_SYSTEM	NVP-AUY922	135539077	0.098927558
ACH-000803	COLO668_LUNG	tanespimycin	6505803	0.098941085
ACH-000472	HSC2_UPPER_AERODIGESTIVE_TRACT	tanespimycin	6505803	0.09898619
ACH-000732	PECAPJ41CLONED2_UPPER_AERODIGESTIVE_TRACT	ispinesib	6851740	0.099105763
ACH-000667	HCC44_LUNG	temoporfin	60751	0.099107126
ACH-000384	SW780_URINARY_TRACT	NVP-AUY922	135539077	0.099131575
ACH-000280	SNU840_OVARY	temsirolimus	129009966	0.099214659
ACH-000344	SNU668_STOMACH	paclitaxel	36314	0.099225021
ACH-000469	YH13_CENTRAL_NERVOUS_SYSTEM	elesclomol	300471	0.099257595
ACH-000888	NCIH1793_LUNG	tanespimycin	6505803	0.0992652
ACH-000361	SKHEP1_LIVER	temoporfin	60751	0.099265434
ACH-000441	SH4_SKIN	vincristine	5388993	0.099294042
ACH-000853	NCIH661_LUNG	tanespimycin	6505803	0.099323549
ACH-000758	MKN74_STOMACH	cabazitaxel	129009963	0.099329356
ACH-000936	EFO27_OVARY	ganetespib	135564985	0.099497956
ACH-000260	SKNAS_AUTONOMIC_GANGLIA	BAY-87-2243	67377767	0.099542439
ACH-000909	JHUEM2_ENDOMETRIUM	temsirolimus	129009966	0.099574914
ACH-000862	KMBC2_URINARY_TRACT	ouabain	11527152	0.099637876
ACH-000376	SF295_CENTRAL_NERVOUS_SYSTEM	everolimus	6442177	0.099770404
ACH-000592	TM31_CENTRAL_NERVOUS_SYSTEM	ingenol-mebutate	6918670	0.099842072
ACH-000441	SH4_SKIN	floxuridine	5702211	0.09991965
ACH-000542	HEYA8_OVARY	trametinib	11707110	0.099936988
ACH-000750	LOXIMVI_SKIN	piperazine	4837	0.099966843

ACH-000528	ABC1_LUNG	tanespimycin	6505803	0.100030815
ACH-000972	HEC151_ENDOMETRIUM	litronesib	25167017	0.100129703
ACH-000324	JHOC5_OVARY	GSK461364	15983966	0.100148305
ACH-000292	NCIH841_LUNG	piperazine	4837	0.10022112
ACH-000976	HUCCT1_BILIARY_TRACT	litronesib	25167017	0.100265902
ACH-000822	SKMEL24_SKIN	filanesib	44224257	0.100269519
ACH-000587	NCIH1975_LUNG	litronesib	25167017	0.100272804
ACH-000549	SNU1076_UPPER_AERODIGESTIVE_TRACT	ingenol-mebutate	6918670	0.10028375
ACH-000927	BT474_BREAST	selinexor	71481097	0.100398799
ACH-000535	BXPC3_PANCREAS	filanesib	44224257	0.100529392
ACH-000661	WM1799_SKIN	ispinesib	6851740	0.100548527
ACH-000781	NCIH2023_LUNG	pralatrexate	148121	0.10056196
ACH-000313	KMRC3_KIDNEY	paclitaxel	36314	0.100569025
ACH-000890	SW1271_LUNG	rigosertib	6918736	0.10057775
ACH-000022	PATU8988S_PANCREAS	tanespimycin	6505803	0.100582263
ACH-000270	HPAC_PANCREAS	BNC105	24786555	0.100618778
ACH-000250	KMRC20_KIDNEY	pralatrexate	148121	0.100822639
ACH-000544	OE21_OESOPHAGUS	temsirolimus	129009966	0.100859606
ACH-000812	COLO783_SKIN	ispinesib	6851740	0.100873548
ACH-000721	HMC18_BREAST	temoporfin	60751	0.100886129
ACH-000657	A2780_OVARY	NVP-BEZ235	11977753	0.100912357
ACH-000008	A101D_SKIN	vinflunine	11967282	0.100972023
ACH-000132	JHOS2_OVARY	temsirolimus	129009966	0.100989013
ACH-000452	TE8_OESOPHAGUS	ingenol-mebutate	6918670	0.101017141
ACH-000211	DAOY_CENTRAL_NERVOUS_SYSTEM	everolimus	6442177	0.101023198
ACH-000313	KMRC3_KIDNEY	pralatrexate	148121	0.101050836
ACH-000222	ASPC1_PANCREAS	fluocinolone-acetonide	6215	0.101072668
ACH-000847	HGC27_STOMACH	litronesib	25167017	0.101144707
ACH-000408	TE5_OESOPHAGUS	rigosertib	6918736	0.10115167
ACH-000318	TE10_OESOPHAGUS	vinflunine	11967282	0.101159929
ACH-000787	LXF289_LUNG	CYT-997	11351021	0.101210833
ACH-000102	GMS10_CENTRAL_NERVOUS_SYSTEM	BI-2536	11364421	0.101246033
ACH-000368	SNU1105_CENTRAL_NERVOUS_SYSTEM	idasanutlin	53358942	0.101337195
ACH-000845	NCIH1373_LUNG	litronesib	25167017	0.101375163
ACH-000503	BICR16_UPPER_AERODIGESTIVE_TRACT	ingenol-mebutate	6918670	0.10152361
ACH-000384	SW780_URINARY_TRACT	ganetespib	135564985	0.101535235
ACH-000023	PATU8988T_PANCREAS	temsirolimus	129009966	0.101560546
ACH-000674	NUGC4_STOMACH	temoporfin	60751	0.10160077
ACH-000209	SNU1079_BILIARY_TRACT	pralatrexate	148121	0.101613481
ACH-000219	A375_SKIN	tanespimycin	6505803	0.101629308
ACH-000796	MCAS_OVARY	SNS-314	24995524	0.101634507
ACH-000750	LOXIMVI_SKIN	temoporfin	60751	0.101645641
ACH-000210	CADOES1_BONE	alvespimycin	5288674	0.101708202

ACH-000558	A172_CENTRAL_NERVOUS_SYSTEM	pralatrexate	148121	0.101717642
ACH-000118	HUPT3_PANCREAS	ispinesib	6851740	0.101742048
ACH-000804	NB1_AUTONOMIC_GANGLIA	temsirolimus	129009966	0.101786598
ACH-000427	NCIN87_STOMACH	BAY-87-2243	67377767	0.101813541
ACH-000869	NCIH1568_LUNG	ouabain	11527152	0.101815973
ACH-000899	WM88_SKIN	tanespimycin	6505803	0.101825022
ACH-000479	KNS81_CENTRAL_NERVOUS_SYSTEM	paclitaxel	36314	0.101828575
ACH-000678	MKN7_STOMACH	KX2-391	23635314	0.101921086
ACH-000603	BEN_LUNG	tanespimycin	6505803	0.101985404
ACH-000266	SNU213_PANCREAS	tanespimycin	6505803	0.102009914
ACH-000958	SW48_LARGE_INTESTINE	BAY-87-2243	67377767	0.10202614
ACH-000311	NCIH2122_LUNG	panobinostat	6918837	0.102222699
ACH-000296	OUMS23_LARGE_INTESTINE	paclitaxel	36314	0.102329913
ACH-000449	MESSA_SOFT_TISSUE	litronesib	25167017	0.102330411
ACH-000997	HCT15_LARGE_INTESTINE	temsirolimus	129009966	0.102384254
ACH-000417	PANC0813_PANCREAS	ispinesib	6851740	0.102405367
ACH-000222	ASPC1_PANCREAS	paclitaxel	36314	0.102430743
ACH-000827	WM793_SKIN	mubritinib	6444692	0.102438253
ACH-000845	NCIH1373_LUNG	ingenol-mebutate	6918670	0.102455127
ACH-000502	TCCPAN2_PANCREAS	BNC105	24786555	0.102457969
ACH-000613	HOS_BONE	temsirolimus	129009966	0.10256568
ACH-000785	NCIH2126_LUNG	temsirolimus	129009966	0.10258854
ACH-000270	HPAC_PANCREAS	paclitaxel	36314	0.102618872
ACH-000625	HEP3B217_LIVER	tanespimycin	6505803	0.102657005
ACH-000570	YKG1_CENTRAL_NERVOUS_SYSTEM	elesclomol	300471	0.102679248
ACH-000955	SNU407_LARGE_INTESTINE	litronesib	25167017	0.10271558
ACH-000796	MCAS_OVARY	temoporfin	60751	0.102734404
ACH-000159	OSRC2_KIDNEY	elesclomol	300471	0.102769358
ACH-000427	NCIN87_STOMACH	pralatrexate	148121	0.102827666
ACH-000308	EFO21_OVARY	ingenol-mebutate	6918670	0.102842187
ACH-000309	SKLU1_LUNG	filanesib	44224257	0.102874721
ACH-000791	RERFLCAD1_LUNG	MLN0128	45375953	0.102889305
ACH-000787	LXF289_LUNG	KX2-391	23635314	0.102932991
ACH-000266	SNU213_PANCREAS	vindesine	40839	0.102935086
ACH-000102	GMS10_CENTRAL_NERVOUS_SYSTEM	CYT-997	11351021	0.103036383
ACH-000332	YAPC_PANCREAS	vinflunine	11967282	0.103051817
ACH-000123	COV434_OVARY	temsirolimus	129009966	0.103099395
ACH-000344	SNU668_STOMACH	pralatrexate	148121	0.103144572
ACH-000599	PATU8902_PANCREAS	temsirolimus	129009966	0.103309825
ACH-000468	PK45H_PANCREAS	MPI-0479605	46909588	0.103313307
ACH-000054	HT1080_SOFT_TISSUE	barasertib-HQPA	16007391	0.103337853
ACH-000900	NCIH23_LUNG	NVP-BEZ235	11977753	0.103391973
ACH-000968	COLO792_SKIN	raltitrexed	135400182	0.103525646

ACH-000832	CAL27_UPPER_AERODIGESTIVE_TRACT	temoporfin	60751	0.103527598
ACH-000927	BT474_BREAST	alvespimycin	5288674	0.103594501
ACH-000822	SKMEL24_SKIN	KX2-391	23635314	0.103595518
ACH-000048	TOV112D_OVARY	temoporfin	60751	0.103669723
ACH-000562	HCC78_LUNG	litronesib	25167017	0.103790704
ACH-000759	MDAMB175VII_BREAST	poziotinib	25127713	0.103822208
ACH-000909	JHUEM2_ENDOMETRIUM	rigosertib	6918736	0.103899849
ACH-000563	EBC1_LUNG	elesclomol	300471	0.103905352
ACH-000329	CCFSTTG1_CENTRAL_NERVOUS_SYSTEM	BNC105	24786555	0.104079674
ACH-000209	SNU1079_BILIARY_TRACT	paclitaxel	36314	0.10409609
ACH-000018	T24_URINARY_TRACT	paclitaxel	36314	0.104118465
ACH-000376	SF295_CENTRAL_NERVOUS_SYSTEM	docetaxel	148124	0.104129536
ACH-000730	SKMEL5_SKIN	temsirolimus	129009966	0.104285163
ACH-000322	HT144_SKIN	NVP-BEZ235	11977753	0.10429517
ACH-000428	UO31_KIDNEY	filanesib	44224257	0.104328523
ACH-000665	SKMES1_LUNG	ingenol-mebutate	6918670	0.104376929
ACH-000967	SNUC2A_LARGE_INTESTINE	D-64131	3921152	0.104398322
ACH-000296	OUMS23_LARGE_INTESTINE	pralatrexate	148121	0.104586547
ACH-000153	NCIH2052_PLEURA	TAK-733	24963252	0.104609036
ACH-000420	SNU449_LIVER	temsirolimus	129009966	0.104617544
ACH-000062	RERFLCMS_LUNG	temoporfin	60751	0.10465452
ACH-000161	CORL105_LUNG	paclitaxel	36314	0.10465554
ACH-000579	UACC257_SKIN	BI-2536	11364421	0.104728397
ACH-000802	BFTC905_URINARY_TRACT	pralatrexate	148121	0.104731903
ACH-000566	SW1710_URINARY_TRACT	idasanutlin	53358942	0.104738808
ACH-000765	WM983B_SKIN	vincristine	5388993	0.104849296
ACH-000836	YD15_SALIVARY_GLAND	ingenol-mebutate	6918670	0.104880065
ACH-000266	SNU213_PANCREAS	NVP-AUY922	135539077	0.104934549
ACH-000688	OV7_OVARY	ingenol-mebutate	6918670	0.104950869
ACH-000351	MKN1_STOMACH	idasanutlin	53358942	0.104981965
ACH-000441	SH4_SKIN	mubritinib	6444692	0.104982873
ACH-000991	SNU81_LARGE_INTESTINE	ispinesib	6851740	0.10498491
ACH-000971	HCT116_LARGE_INTESTINE	temsirolimus	129009966	0.104996568
ACH-000984	HEC6_ENDOMETRIUM	tanespimycin	6505803	0.105099134
ACH-000662	CORL23_LUNG	elesclomol	300471	0.105129308
ACH-000376	SF295_CENTRAL_NERVOUS_SYSTEM	tanespimycin	6505803	0.105152971
ACH-000231	KALS1_CENTRAL_NERVOUS_SYSTEM	rigosertib	6918736	0.105205107
ACH-000408	TE5_OESOPHAGUS	pralatrexate	148121	0.105207224
ACH-000189	RCC10RGB_KIDNEY	paclitaxel	36314	0.1052272
ACH-000277	HCC1419_BREAST	pralatrexate	148121	0.105302019
ACH-000924	NCIH2172_LUNG	everolimus	6442177	0.105317063
ACH-000716	TT2609C02_THYROID	temsirolimus	129009966	0.105375251
ACH-000605	TE6_OESOPHAGUS	temoporfin	60751	0.105405753

ACH-000302	SNU1077_ENDOMETRIUM	docetaxel	148124	0.105445137
ACH-000302	SNU1077_ENDOMETRIUM	alvespimycin	5288674	0.105467976
ACH-000984	HEC6_ENDOMETRIUM	pralatrexate	148121	0.105523917
ACH-000657	A2780_OVARY	irinotecan	60838	0.10552395
ACH-000936	EFO27_OVARY	pralatrexate	148121	0.105563279
ACH-000758	MKN74_STOMACH	cephalomannine	6436208	0.105582867
ACH-000758	MKN74_STOMACH	vindesine	40839	0.105609395
ACH-000970	SNUC5_LARGE_INTESTINE	alvespimycin	5288674	0.105609553
ACH-000527	OVISE_OVARY	BAY-87-2243	67377767	0.105623612
ACH-000397	TEN_ENDOMETRIUM	irinotecan	60838	0.105625231
ACH-000628	NCIH596_LUNG	ingenol-mebutate	6918670	0.105664572
ACH-000561	TT_OESOPHAGUS	barasertib-HQPA	16007391	0.105703381
ACH-000674	NUGC4_STOMACH	NVP-BEZ235	11977753	0.105711945
ACH-000778	HSC3_UPPER_AERODIGESTIVE_TRACT	litronesib	25167017	0.105713989
ACH-000810	SKMEL30_SKIN	BI-2536	11364421	0.105720659
ACH-000007	LS513_LARGE_INTESTINE	tanespimycin	6505803	0.105746466
ACH-000974	SNGM_ENDOMETRIUM	everolimus	6442177	0.105994736
ACH-000274	HS852T_SKIN	FK-866	6914657	0.106048323
ACH-000139	PANC0327_PANCREAS	piperazine	4837	0.106061274
ACH-000434	NCIH1915_LUNG	ingenol-mebutate	6918670	0.106065408
ACH-000018	T24_URINARY_TRACT	pralatrexate	148121	0.106088315
ACH-000332	YAPC_PANCREAS	elesclomol	300471	0.106161493
ACH-000174	CAL62_THYROID	MLN0128	45375953	0.106256296
ACH-000359	MG63_BONE	tanespimycin	6505803	0.106279678
ACH-000603	BEN_LUNG	temsirolimus	129009966	0.10644726
ACH-000880	AGS_STOMACH	litronesib	25167017	0.106603629
ACH-000425	UACC62_SKIN	temsirolimus	129009966	0.106637863
ACH-000335	MSTO211H_PLEURA	temsirolimus	129009966	0.106642221
ACH-000921	NCIH1339_LUNG	ingenol-mebutate	6918670	0.106739315
ACH-000542	HEYA8_OVARY	elesclomol	300471	0.106780708
ACH-000244	DKMG_CENTRAL_NERVOUS_SYSTEM	ispinesib	6851740	0.106801503
ACH-000484	VMRCRCW_KIDNEY	pralatrexate	148121	0.106880381
ACH-000107	CAPAN2_PANCREAS	ingenol-mebutate	6918670	0.106961687
ACH-000484	VMRCRCW_KIDNEY	filanesib	44224257	0.106984771
ACH-000274	HS852T_SKIN	alvespimycin	5288674	0.10703377
ACH-000222	ASPC1_PANCREAS	tanespimycin	6505803	0.10704107
ACH-000324	JHOC5_OVARY	rigosertib	6918736	0.107219314
ACH-000880	AGS_STOMACH	tanespimycin	6505803	0.10732169
ACH-000532	SNU61_LARGE_INTESTINE	ingenol-mebutate	6918670	0.107329239
ACH-000384	SW780_URINARY_TRACT	BI-2536	11364421	0.107397467
ACH-000159	OSRC2_KIDNEY	KX2-391	23635314	0.107466811
ACH-000447	NCIH2228_LUNG	ispinesib	6851740	0.107469969
ACH-000749	DMS273_LUNG	idasanutlin	53358942	0.107488061

ACH-000628	NCIH596_LUNG	GSK461364	15983966	0.107541938
ACH-000800	NCIH446_LUNG	irinotecan	60838	0.107565594
ACH-000957	LS180_LARGE_INTESTINE	pralatrexate	148121	0.107569263
ACH-000842	SW480_LARGE_INTESTINE	trametinib	11707110	0.107637618
ACH-000012	HCC827_LUNG	rigosertib	6918736	0.107749123
ACH-000957	LS180_LARGE_INTESTINE	paclitaxel	36314	0.107755075
ACH-000384	SW780_URINARY_TRACT	tanespimycin	6505803	0.107939564
ACH-000939	SKUT1_SOFT_TISSUE	temoporfin	60751	0.107954086
ACH-000943	RKO_LARGE_INTESTINE	NVP-BEZ235	11977753	0.10797141
ACH-000730	SKMEL5_SKIN	trametinib	11707110	0.107981626
ACH-000657	A2780_OVARY	temoporfin	60751	0.107984375
ACH-000425	UACC62_SKIN	piperazine	4837	0.107990561
ACH-000994	HEC59_ENDOMETRIUM	litronesib	25167017	0.108054472
ACH-000046	ACHN_KIDNEY	litronesib	25167017	0.108078674
ACH-000471	LI7_LIVER	ingenol-mebutate	6918670	0.108088153
ACH-000427	NCIN87_STOMACH	combretastatin-A-4	5351344	0.108099467
ACH-000320	PSN1_PANCREAS	idasanutlin	53358942	0.108131887
ACH-000403	NCIH747_LARGE_INTESTINE	docetaxel	148124	0.108235084
ACH-000652	SUIT2_PANCREAS	pralatrexate	148121	0.108353091
ACH-000766	NCIH1648_LUNG	piperazine	4837	0.108368673
ACH-000924	NCIH2172_LUNG	GSK461364	15983966	0.108447797
ACH-000102	GMS10_CENTRAL_NERVOUS_SYSTEM	ingenol-mebutate	6918670	0.108490074
ACH-000008	A101D_SKIN	piperazine	4837	0.108520659
ACH-000123	COV434_OVARY	NVP-BEZ235	11977753	0.10857769
ACH-000863	DBTRG05MG_CENTRAL_NERVOUS_SYSTEM	elesclomol	300471	0.108594995
ACH-000868	HCC1195_LUNG	D-64131	3921152	0.108614658
ACH-000437	SW1088_CENTRAL_NERVOUS_SYSTEM	temsirolimus	129009966	0.108618243
ACH-000351	MKN1_STOMACH	piperazine	4837	0.108706124
ACH-000766	NCIH1648_LUNG	NVP-BEZ235	11977753	0.108725936
ACH-000159	OSRC2_KIDNEY	BNC105	24786555	0.10873119
ACH-000804	NB1_AUTONOMIC_GANGLIA	ingenol-mebutate	6918670	0.108738598
ACH-000674	NUGC4_STOMACH	MLN0128	45375953	0.108761535
ACH-000669	SW900_LUNG	pralatrexate	148121	0.108763757
ACH-000389	H4_CENTRAL_NERVOUS_SYSTEM	elesclomol	300471	0.108764441
ACH-000845	NCIH1373_LUNG	ispinesib	6851740	0.108801293
ACH-000164	PANC1_PANCREAS	LY2606368	46700756	0.108823969
ACH-000277	HCC1419_BREAST	verubulin	11414799	0.10884778
ACH-000528	ABC1_LUNG	piperazine	4837	0.108911778
ACH-001075	NCIH292_LUNG	litronesib	25167017	0.109016738
ACH-000957	LS180_LARGE_INTESTINE	tanespimycin	6505803	0.109087051
ACH-000954	HEC1A_ENDOMETRIUM	rigosertib	6918736	0.109133831
ACH-000890	SW1271_LUNG	ingenol-mebutate	6918670	0.109141601
ACH-000018	T24_URINARY_TRACT	tanespimycin	6505803	0.10923703

ACH-000351	MKN1_STOMACH	irinotecan	60838	0.109238378
ACH-000458	CJM_SKIN	elesclomol	300471	0.109244404
ACH-000096	G401_SOFT_TISSUE	ingenol-mebutate	6918670	0.109276198
ACH-000945	NCIH650_LUNG	temsirolimus	129009966	0.109344048
ACH-000941	HEC1B_ENDOMETRIUM	pralatrexate	148121	0.109351034
ACH-000627	LCLC103H_LUNG	temsirolimus	129009966	0.109464365
ACH-000648	NCIH28_PLEURA	docetaxel	148124	0.109491353
ACH-000812	COLO783_SKIN	everolimus	6442177	0.109522684
ACH-000778	HSC3_UPPER_AERODIGESTIVE_TRACT	idasanutlin	53358942	0.109593271
ACH-000222	ASPC1_PANCREAS	desonide	5311066	0.109608042
ACH-000909	JHUEM2_ENDOMETRIUM	temoporfin	60751	0.109619085
ACH-000141	SNU308_BILIARY_TRACT	tanespimycin	6505803	0.109629715
ACH-000493	SNU423_LIVER	piperazine	4837	0.109684769
ACH-000614	RVH421_SKIN	temsirolimus	129009966	0.109711914
ACH-000778	HSC3_UPPER_AERODIGESTIVE_TRACT	temoporfin	60751	0.10971407
ACH-000678	MKN7_STOMACH	CYT-997	11351021	0.109774747
ACH-000062	RERFLCMS_LUNG	temsirolimus	129009966	0.109851341
ACH-000776	ONS76_CENTRAL_NERVOUS_SYSTEM	napabucasin	10331844	0.109882318
ACH-000680	SW948_LARGE_INTESTINE	alvespimycin	5288674	0.109908597
ACH-000808	HUH28_BILIARY_TRACT	tanespimycin	6505803	0.109925353
ACH-000454	HCC95_LUNG	pralatrexate	148121	0.11005072
ACH-000482	RERFLCKJ_LUNG	temoporfin	60751	0.110081814
ACH-000142	CAL29_URINARY_TRACT	temoporfin	60751	0.110156233
ACH-000274	HS852T_SKIN	taltobulin	6918637	0.110163773
ACH-000481	NCIH2170_LUNG	ispinesib	6851740	0.110200033
ACH-000565	RCM1_LARGE_INTESTINE	idasanutlin	53358942	0.110203748
ACH-000352	HCC1428_BREAST	filanesib	44224257	0.110241192
ACH-001075	NCIH292_LUNG	D-64131	3921152	0.110293806
ACH-000991	SNU81_LARGE_INTESTINE	ingenol-mebutate	6918670	0.110305966
ACH-000959	SNUC4_LARGE_INTESTINE	elesclomol	300471	0.11032144
ACH-000997	HCT15_LARGE_INTESTINE	tanespimycin	6505803	0.110350217
ACH-000765	WM983B_SKIN	BI-2536	11364421	0.110365826
ACH-000822	SKMEL24_SKIN	pralatrexate	148121	0.110369339
ACH-000480	HUH7_LIVER	bortezomib	387447	0.110461337
ACH-000913	ESS1_ENDOMETRIUM	BAY-87-2243	67377767	0.110466693
ACH-000433	CAKI1_KIDNEY	combretastatin-A-4	5351344	0.110494309
ACH-000738	GB1_CENTRAL_NERVOUS_SYSTEM	GSK461364	15983966	0.1105786
ACH-000527	OVISE_OVARY	ispinesib	6851740	0.110609486
ACH-001321	TT_THYROID	NVP-AUY922	135539077	0.110716352
ACH-000921	NCIH1339_LUNG	altretamine	2123	0.110790921
ACH-000684	KMRC1_KIDNEY	elesclomol	300471	0.110793977
ACH-000186	NCIH2444_LUNG	piperazine	4837	0.11088456
ACH-000389	H4_CENTRAL_NERVOUS_SYSTEM	docetaxel	148124	0.110898363

ACH-000384	SW780_URINARY_TRACT	pralatrexate	148121	0.11090252
ACH-000648	NCIH28_PLEURA	BAY-87-2243	67377767	0.11106381
ACH-000701	RMUGS_OVARY	dinaciclib	46926350	0.111145469
ACH-000433	CAKI1_KIDNEY	BNC105	24786555	0.111323357
ACH-000994	HEC59_ENDOMETRIUM	rigosertib	6918736	0.111327853
ACH-000605	TE6_OESOPHAGUS	irinotecan	60838	0.111373301
ACH-000549	SNU1076_UPPER_AERODIGESTIVE_TRACT	BI-2536	11364421	0.111375052
ACH-000681	A549_LUNG	vincristine	5388993	0.111433636
ACH-000846	FADU_UPPER_AERODIGESTIVE_TRACT	temoporfin	60751	0.11145129
ACH-000885	TOV21G_OVARY	talazoparib	135565082	0.111567395
ACH-000428	UO31_KIDNEY	raltitrexed	135400182	0.111567956
ACH-000599	PATU8902_PANCREAS	elesclomol	300471	0.111618949
ACH-000270	HPAC_PANCREAS	combretastatin-A-4	5351344	0.111631896
ACH-000210	CADOES1_BONE	BNC105	24786555	0.111637282
ACH-000013	ONCODG1_OVARY	temsirolimus	129009966	0.111669456
ACH-000720	TCCSUP_URINARY_TRACT	ispinesib	6851740	0.111697469
ACH-000396	J82_URINARY_TRACT	temsirolimus	129009966	0.111715579
ACH-000223	HCC1937_BREAST	pralatrexate	148121	0.111752306
ACH-000792	BFTC909_KIDNEY	pralatrexate	148121	0.111792156
ACH-000579	UACC257_SKIN	tanespimycin	6505803	0.111950975
ACH-000223	HCC1937_BREAST	BNC105	24786555	0.111961092
ACH-000376	SF295_CENTRAL_NERVOUS_SYSTEM	BAY-87-2243	67377767	0.111971861
ACH-000495	TUHR4TKB_KIDNEY	elesclomol	300471	0.112080422
ACH-000480	HUH7_LIVER	verubulin	11414799	0.112177258
ACH-000972	HEC151_ENDOMETRIUM	ispinesib	6851740	0.112187184
ACH-000990	HEC108_ENDOMETRIUM	ingenol-mebutate	6918670	0.112284015
ACH-000967	SNUC2A_LARGE_INTESTINE	tanespimycin	6505803	0.112322457
ACH-000404	K029AX_SKIN	tanespimycin	6505803	0.112370024
ACH-000736	SNU601_STOMACH	MLN0128	45375953	0.11242618
ACH-000899	WM88_SKIN	paclitaxel	36314	0.112441297
ACH-000921	NCIH1339_LUNG	temsirolimus	129009966	0.112494749
ACH-000630	YD8_UPPER_AERODIGESTIVE_TRACT	elesclomol	300471	0.112502732
ACH-000651	SW620_LARGE_INTESTINE	NVP-BEZ235	11977753	0.112552887
ACH-000765	WM983B_SKIN	filanesib	44224257	0.112577089
ACH-000868	HCC1195_LUNG	GSK461364	15983966	0.112580406
ACH-000895	CL34_LARGE_INTESTINE	temoporfin	60751	0.112595615
ACH-000774	RERFLCAD2_LUNG	docetaxel	148124	0.112660232
ACH-000480	HUH7_LIVER	filanesib	44224257	0.112672078
ACH-000320	PSN1_PANCREAS	temoporfin	60751	0.112824123
ACH-000469	YH13_CENTRAL_NERVOUS_SYSTEM	temsirolimus	129009966	0.112961557
ACH-000625	HEP3B217_LIVER	temsirolimus	129009966	0.113010993
ACH-000941	HEC1B_ENDOMETRIUM	filanesib	44224257	0.113021135
ACH-000863	DBTRG05MG_CENTRAL_NERVOUS_SYSTEM	temoporfin	60751	0.113032335

ACH-000880	AGS_STOMACH	vinflunine	11967282	0.113035391
ACH-000674	NUGC4_STOMACH	piperazine	4837	0.113037629
ACH-000237	JHOM1_OVARY	temsirolimus	129009966	0.113064449
ACH-000886	NCIH2009_LUNG	temsirolimus	129009966	0.113098581
ACH-000035	NCIH1650_LUNG	floxuridine	5702211	0.113123273
ACH-000331	ISTMES2_PLEURA	FK-866	6914657	0.113130011
ACH-000785	NCIH2126_LUNG	ingenol-mebutate	6918670	0.113134447
ACH-000335	MSTO211H_PLEURA	NVP-BEZ235	11977753	0.113148308
ACH-000133	HS729_SOFT_TISSUE	BMS-626529	11317439	0.113187268
ACH-000732	PECAPJ41CLONED2_UPPER_AERODIGESTIVE_TRACT	ingenol-mebutate	6918670	0.113187828
ACH-000991	SNU81_LARGE_INTESTINE	litronesib	25167017	0.113233822
ACH-000307	PK1_PANCREAS	temsirolimus	129009966	0.113330648
ACH-000305	ECGI10_OESOPHAGUS	tosedostat	15547703	0.113331031
ACH-000939	SKUT1_SOFT_TISSUE	piperazine	4837	0.113354491
ACH-000209	SNU1079_BILIARY_TRACT	CYT-997	11351021	0.113478945
ACH-000768	MDAMB231_BREAST	tanespimycin	6505803	0.11350801
ACH-000945	NCIH650_LUNG	dasatinib	3062316	0.113517929
ACH-000826	CAL12T_LUNG	vincristine	5388993	0.113649796
ACH-000561	TT_OESOPHAGUS	MLN0128	45375953	0.113669651
ACH-000018	T24_URINARY_TRACT	ingenol-mebutate	6918670	0.113766286
ACH-000397	TEN_ENDOMETRIUM	pralatrexate	148121	0.113888766
ACH-000900	NCIH23_LUNG	ingenol-mebutate	6918670	0.113942906
ACH-000423	SKMEL3_SKIN	vincristine	5388993	0.113984755
ACH-000133	HS729_SOFT_TISSUE	ispinesib	6851740	0.11402282
ACH-000774	RERFLCAD2_LUNG	tanespimycin	6505803	0.114024514
ACH-000643	HDQP1_BREAST	litronesib	25167017	0.114054821
ACH-000270	HPAC_PANCREAS	pralatrexate	148121	0.11408623
ACH-000209	SNU1079_BILIARY_TRACT	12-O-tetradecanoylphorbol-13-acetate	27924	0.114201561
ACH-000736	SNU601_STOMACH	rigosertib	6918736	0.11425945
ACH-000316	SNU886_LIVER	idasanutlin	53358942	0.11426165
ACH-000670	SBC5_LUNG	temoporfin	60751	0.11435027
ACH-000774	RERFLCAD2_LUNG	BAY-87-2243	67377767	0.114391028
ACH-000318	TE10_OESOPHAGUS	D-64131	3921152	0.114434507
ACH-000066	HCC4006_LUNG	AZD8931	11488320	0.114486081
ACH-000897	FTC238_THYROID	temsirolimus	129009966	0.114544132
ACH-000897	FTC238_THYROID	tanespimycin	6505803	0.114564261
ACH-000496	NCIH1792_LUNG	cobimetinib	16222096	0.114567979
ACH-000155	SW1990_PANCREAS	idasanutlin	53358942	0.114577
ACH-000628	NCIH596_LUNG	temsirolimus	129009966	0.11458604
ACH-000384	SW780_URINARY_TRACT	BNC105	24786555	0.11463689
ACH-000278	COV362_OVARY	litronesib	25167017	0.114648403
ACH-000500	SNU46_UPPER_AERODIGESTIVE_TRACT	tanespimycin	6505803	0.114697577
ACH-001318	PLCPRF5_LIVER	7-aminocephalosporanic-acid	441328	0.114723808

ACH-000837	NCIH322_LUNG	litronesib	25167017	0.114749108
ACH-000232	U251MG_CENTRAL_NERVOUS_SYSTEM	NVP-BEZ235	11977753	0.114755862
ACH-000553	SQ1_LUNG	litronesib	25167017	0.115007017
ACH-000408	TE5_OESOPHAGUS	MPI-0479605	46909588	0.115086891
ACH-000228	BICR31_UPPER_AERODIGESTIVE_TRACT	rigosertib	6918736	0.115124736
ACH-000542	HEYA8_OVARY	NVP-BEZ235	11977753	0.115160792
ACH-000849	MDAMB468_BREAST	litronesib	25167017	0.115207108
ACH-000945	NCIH650_LUNG	idasanutlin	53358942	0.115282736
ACH-000118	HUPT3_PANCREAS	elesclomol	300471	0.115361015
ACH-000759	MDAMB175VII_BREAST	epothilone-b	129010071	0.115376037
ACH-000133	HS729_SOFT_TISSUE	everolimus	6442177	0.115410204
ACH-000444	LU99_LUNG	tosedostat	15547703	0.115511142
ACH-000014	HS294T_SKIN	tanespimycin	6505803	0.115511487
ACH-000704	OAW42_OVARY	tanespimycin	6505803	0.115575144
ACH-000713	CAOV3_OVARY	rigosertib	6918736	0.115691017
ACH-000393	HLF_LIVER	altretamine	2123	0.115728979
ACH-000720	TCCSUP_URINARY_TRACT	ingenol-mebutate	6918670	0.115819192
ACH-000133	HS729_SOFT_TISSUE	idasanutlin	53358942	0.115844186
ACH-000471	LI7_LIVER	temsirolimus	129009966	0.11586
ACH-000669	SW900_LUNG	elesclomol	300471	0.115883473
ACH-000579	UACC257_SKIN	vincristine	5388993	0.115910595
ACH-000869	NCIH1568_LUNG	rigosertib	6918736	0.115991461
ACH-000696	OVCAR8_OVARY	litronesib	25167017	0.116041849
ACH-000592	TM31_CENTRAL_NERVOUS_SYSTEM	vindesine	40839	0.116063991
ACH-000609	SF126_CENTRAL_NERVOUS_SYSTEM	tanespimycin	6505803	0.116065423
ACH-000945	NCIH650_LUNG	temoporfin	60751	0.116141377
ACH-000774	RERFLCAD2_LUNG	paclitaxel	36314	0.116193246
ACH-000826	CAL12T_LUNG	BI-2536	11364421	0.116259389
ACH-000990	HEC108_ENDOMETRIUM	ouabain	11527152	0.116278813
ACH-000139	PANC0327_PANCREAS	temoporfin	60751	0.116311045
ACH-000176	LOUNH91_LUNG	AS-703026	44187362	0.116335104
ACH-000715	SNU1214_UPPER_AERODIGESTIVE_TRACT	ingenol-mebutate	6918670	0.11638962
ACH-000364	U2OS_BONE	tanespimycin	6505803	0.116398986
ACH-000976	HUCCT1_BILIARY_TRACT	ispinesib	6851740	0.116455829
ACH-000843	HARA_LUNG	tosedostat	15547703	0.116518358
ACH-000836	YD15_SALIVARY_GLAND	GSK461364	15983966	0.116563544
ACH-000243	DANG_PANCREAS	temoporfin	60751	0.11656555
ACH-000274	HS852T_SKIN	NSC-319726	5921672	0.116592565
ACH-000118	HUPT3_PANCREAS	idasanutlin	53358942	0.116606218
ACH-000102	GMS10_CENTRAL_NERVOUS_SYSTEM	GSK461364	15983966	0.116649732
ACH-000147	T47D_BREAST	tanespimycin	6505803	0.116686679
ACH-000022	PATU8988S_PANCREAS	filanesib	44224257	0.116694932
ACH-000939	SKUT1_SOFT_TISSUE	altretamine	2123	0.116733021

ACH-000178	HS766T_PANCREAS	cobimetinib	16222096	0.116739213
ACH-000389	H4_CENTRAL_NERVOUS_SYSTEM	ispinesib	6851740	0.116781199
ACH-000670	SBC5_LUNG	tosedostat	15547703	0.116822741
ACH-000599	PATU8902_PANCREAS	ispinesib	6851740	0.116836447
ACH-000296	OUMS23_LARGE_INTESTINE	temsirolimus	129009966	0.116968446
ACH-000535	BXPC3_PANCREAS	vinblastine	13342	0.116973134
ACH-000738	GB1_CENTRAL_NERVOUS_SYSTEM	temsirolimus	129009966	0.117003232
ACH-000433	CAKI1_KIDNEY	paclitaxel	36314	0.117051825
ACH-000803	COLO668_LUNG	elesclomol	300471	0.117051853
ACH-000189	RCC10RGB_KIDNEY	NVP-BEZ235	11977753	0.117194437
ACH-000488	TE11_OESOPHAGUS	temoporfin	60751	0.117246767
ACH-000744	NCIH1623_LUNG	rigosertib	6918736	0.117253244
ACH-000985	LS411N_LARGE_INTESTINE	idasanutlin	53358942	0.11728735
ACH-000900	NCIH23_LUNG	everolimus	6442177	0.117298217
ACH-000898	SNU719_STOMACH	litronesib	25167017	0.117339269
ACH-000163	SW579_THYROID	dabrafenib	44462760	0.117375899
ACH-000941	HEC1B_ENDOMETRIUM	paclitaxel	36314	0.117399431
ACH-000704	OAW42_OVARY	elesclomol	300471	0.117408325
ACH-000420	SNU449_LIVER	paclitaxel	36314	0.117440445
ACH-000630	YD8_UPPER_AERODIGESTIVE_TRACT	docetaxel	148124	0.117458996
ACH-000765	WM983B_SKIN	GSK461364	15983966	0.117482111
ACH-000609	SF126_CENTRAL_NERVOUS_SYSTEM	NVP-BEZ235	11977753	0.11757017
ACH-000967	SNUC2A_LARGE_INTESTINE	ingenol-mebutate	6918670	0.117593279
ACH-000222	ASPC1_PANCREAS	litronesib	25167017	0.117628223
ACH-000277	HCC1419_BREAST	elesclomol	300471	0.117641978
ACH-000096	G401_SOFT_TISSUE	NVP-BEZ235	11977753	0.117892289
ACH-000139	PANC0327_PANCREAS	tipifarnib	159324	0.117934254
ACH-000965	RL952_ENDOMETRIUM	NVP-BEZ235	11977753	0.117938267
ACH-000982	GP2D_LARGE_INTESTINE	temoporfin	60751	0.117971459
ACH-000389	H4_CENTRAL_NERVOUS_SYSTEM	ingenol-mebutate	6918670	0.118046109
ACH-000827	WM793_SKIN	ispinesib	6851740	0.118083879
ACH-001321	TT_THYROID	Ro-4987655	11548630	0.11815912
ACH-000749	DMS273_LUNG	NVP-BEZ235	11977753	0.118170356
ACH-000452	TE8_OESOPHAGUS	KX2-391	23635314	0.118202016
ACH-000582	COLO741_SKIN	ingenol-mebutate	6918670	0.118236639
ACH-000324	JHOC5_OVARY	12-O-tetradecanoylphorbol-13-acetate	27924	0.118367551
ACH-000561	TT_OESOPHAGUS	temoporfin	60751	0.118387504
ACH-000429	A704_KIDNEY	dinaciclib	46926350	0.118451322
ACH-000774	RERFLCAD2_LUNG	dinaciclib	46926350	0.118456842
ACH-000476	JHH4_LIVER	paclitaxel	36314	0.118513181
ACH-000941	HEC1B_ENDOMETRIUM	ingenol-mebutate	6918670	0.118523053
ACH-000364	U2OS_BONE	temoporfin	60751	0.118535301
ACH-000805	COLO679_SKIN	litronesib	25167017	0.118539428

ACH-000669	SW900_LUNG	altretamine	2123	0.118641219
ACH-000018	T24_URINARY_TRACT	AZD8330	16666708	0.118643289
ACH-000324	JHOC5_OVARY	temsirolimus	129009966	0.118649498
ACH-000176	LOUNH91_LUNG	filanesib	44224257	0.1186987
ACH-000527	OVISE_OVARY	D-64131	3921152	0.118801179
ACH-000312	SKNBE2_AUTONOMIC_GANGLIA	tanespimycin	6505803	0.118867294
ACH-000231	KALS1_CENTRAL_NERVOUS_SYSTEM	temsirolimus	129009966	0.118979352
ACH-000868	HCC1195_LUNG	AZD8330	16666708	0.118991628
ACH-000792	BFTC909_KIDNEY	birinapant	49836020	0.11899204
ACH-000007	LS513_LARGE_INTESTINE	ingenol-mebutate	6918670	0.1190526
ACH-000500	SNU46_UPPER_AERODIGESTIVE_TRACT	everolimus	6442177	0.119065249
ACH-000985	LS411N_LARGE_INTESTINE	piperazine	4837	0.119070489
ACH-000587	NCIH1975_LUNG	temsirolimus	129009966	0.119086359
ACH-000899	WM88_SKIN	filanesib	44224257	0.119158409
ACH-000701	RMUGS_OVARY	pralatrexate	148121	0.119198801
ACH-000421	SW837_LARGE_INTESTINE	idasanutlin	53358942	0.11922179
ACH-000759	MDAMB175VII_BREAST	ingenol-mebutate	6918670	0.119233773
ACH-000418	SW1353_BONE	pralatrexate	148121	0.119275512
ACH-000147	T47D_BREAST	litronesib	25167017	0.11941418
ACH-000368	SNU1105_CENTRAL_NERVOUS_SYSTEM	tanespimycin	6505803	0.119443254
ACH-000472	HSC2_UPPER_AERODIGESTIVE_TRACT	GSK461364	15983966	0.119457926
ACH-000384	SW780_URINARY_TRACT	trametinib	11707110	0.119514187
ACH-000161	CORL105_LUNG	elesclomol	300471	0.119562479
ACH-000351	MKN1_STOMACH	NVP-BEZ235	11977753	0.119724782
ACH-000504	SNB75_CENTRAL_NERVOUS_SYSTEM	pralatrexate	148121	0.119807517
ACH-000178	HS766T_PANCREAS	raltitrexed	135400182	0.11981294
ACH-000042	PANC0203_PANCREAS	SNS-314	24995524	0.119893787
ACH-000278	COV362_OVARY	rigosertib	6918736	0.119898074
ACH-000834	UMUC1_URINARY_TRACT	7-aminocephalosporanic-acid	441328	0.119920603
ACH-000012	HCC827_LUNG	tanespimycin	6505803	0.119983418
ACH-000176	LOUNH91_LUNG	pralatrexate	148121	0.119992828
ACH-000189	RCC10RGB_KIDNEY	SNS-314	24995524	0.120118453
ACH-000445	KNS60_CENTRAL_NERVOUS_SYSTEM	elesclomol	300471	0.120121918
ACH-000716	TT2609C02_THYROID	idasanutlin	53358942	0.120147416
ACH-000312	SKNBE2_AUTONOMIC_GANGLIA	temoporfin	60751	0.120209292
ACH-000954	HEC1A_ENDOMETRIUM	NVP-BEZ235	11977753	0.120219249
ACH-000132	JHOS2_OVARY	elesclomol	300471	0.12022111
ACH-000277	HCC1419_BREAST	MPI-0479605	46909588	0.120247083
ACH-000985	LS411N_LARGE_INTESTINE	litronesib	25167017	0.120308036
ACH-000979	DU145_PROSTATE	everolimus	6442177	0.120355088
ACH-000107	CAPAN2_PANCREAS	vincristine	5388993	0.120361889
ACH-000060	PANC1005_PANCREAS	ispinesib	6851740	0.120435856
ACH-000813	T3M10_LUNG	temoporfin	60751	0.120438515

ACH-000863	DBTRG05MG_CENTRAL_NERVOUS_SYSTEM	rigosertib	6918736	0.120440067
ACH-000171	VMRCRCZ_KIDNEY	floxuridine	5702211	0.120462017
ACH-000147	T47D_BREAST	volasertib	10461508	0.120537069
ACH-000091	OV56_OVARY	temoporfin	60751	0.120569136
ACH-000445	KNS60_CENTRAL_NERVOUS_SYSTEM	piperazine	4837	0.120579681
ACH-000650	IGR37_SKIN	idasanutlin	53358942	0.120597544
ACH-000335	MSTO211H_PLEURA	temoporfin	60751	0.120629143
ACH-000086	ACCMESO1_PLEURA	everolimus	6442177	0.120637893
ACH-000376	SF295_CENTRAL_NERVOUS_SYSTEM	KX2-391	23635314	0.120638699
ACH-000164	PANC1_PANCREAS	MPI-0479605	46909588	0.12064524
ACH-000222	ASPC1_PANCREAS	dasatinib	3062316	0.120664515
ACH-000805	COLO679_SKIN	NVP-BEZ235	11977753	0.12069177
ACH-000277	HCC1419_BREAST	pelitinib	6445562	0.120727872
ACH-000758	MKN74_STOMACH	BAY-87-2243	67377767	0.120743166
ACH-000974	SNGM_ENDOMETRIUM	dasatinib	3062316	0.120768492
ACH-000917	TE4_OESOPHAGUS	altretamine	2123	0.120784589
ACH-000713	CAOV3_OVARY	temoporfin	60751	0.120833707
ACH-000264	CALU6_LUNG	idasanutlin	53358942	0.120867069
ACH-000384	SW780_URINARY_TRACT	vinblastine	13342	0.120883407
ACH-001321	TT_THYROID	TAK-733	24963252	0.120976592
ACH-000496	NCIH1792_LUNG	idasanutlin	53358942	0.121047645
ACH-000107	CAPAN2_PANCREAS	BNC105	24786555	0.12113219
ACH-000209	SNU1079_BILIARY_TRACT	ingenol-mebutate	6918670	0.121160178
ACH-000473	RT112_URINARY_TRACT	idasanutlin	53358942	0.121165091
ACH-000182	SNU869_BILIARY_TRACT	ingenol-mebutate	6918670	0.121193097
ACH-000846	FADU_UPPER_AERODIGESTIVE_TRACT	AT13387	11955716	0.121211383
ACH-000517	SNU410_PANCREAS	KX2-391	23635314	0.121256226
ACH-000147	T47D_BREAST	ispinesib	6851740	0.121411315
ACH-000117	EFM192A_BREAST	LY2606368	46700756	0.121469031
ACH-000765	WM983B_SKIN	pralatrexate	148121	0.121540374
ACH-000791	RERFLCAD1_LUNG	pralatrexate	148121	0.121545598
ACH-000561	TT_OESOPHAGUS	NVP-BEZ235	11977753	0.121646406
ACH-000630	YD8_UPPER_AERODIGESTIVE_TRACT	filanesib	44224257	0.121646406
ACH-000756	GI1_CENTRAL_NERVOUS_SYSTEM	temazepam	5391	0.121671233
ACH-000717	COLO680N_OESOPHAGUS	litronesib	25167017	0.121682172
ACH-000384	SW780_URINARY_TRACT	LY2606368	46700756	0.121685012
ACH-000863	DBTRG05MG_CENTRAL_NERVOUS_SYSTEM	temsirolimus	129009966	0.121717174
ACH-000764	SH10TC_STOMACH	piperazine	4837	0.121799056
ACH-000936	EFO27_OVARY	vindesine	40839	0.121802693
ACH-000164	PANC1_PANCREAS	pralatrexate	148121	0.12180276
ACH-000929	NCIH2110_LUNG	dasatinib	3062316	0.121803134
ACH-000117	EFM192A_BREAST	pralatrexate	148121	0.121842919
ACH-000054	HT1080_SOFT_TISSUE	SNS-314	24995524	0.121934266

ACH-000318	TE10_OESOPHAGUS	floxuridine	5702211	0.121948046
ACH-000717	COLO680N_OESOPHAGUS	ingenol-mebutate	6918670	0.122259301
ACH-000270	HPAC_PANCREAS	tanespimycin	6505803	0.122359816
ACH-000266	SNU213_PANCREAS	ganetespib	135564985	0.122364989
ACH-000450	MELHO_SKIN	ispinesib	6851740	0.122403284
ACH-000842	SW480_LARGE_INTESTINE	combretastatin-A-4	5351344	0.122428035
ACH-000302	SNU1077_ENDOMETRIUM	filanesib	44224257	0.122482307
ACH-000553	SQ1_LUNG	piperazine	4837	0.122493925
ACH-000563	EBC1_LUNG	temoporfin	60751	0.122524452
ACH-000756	GI1_CENTRAL_NERVOUS_SYSTEM	volasertib	10461508	0.122531183
ACH-000856	CAL51_BREAST	temsirolimus	129009966	0.122634234
ACH-000159	OSRC2_KIDNEY	combretastatin-A-4	5351344	0.122664343
ACH-000846	FADU_UPPER_AERODIGESTIVE_TRACT	barasertib-HQPA	16007391	0.122665538
ACH-000022	PATU8988S_PANCREAS	pralatrexate	148121	0.122823193
ACH-000738	GB1_CENTRAL_NERVOUS_SYSTEM	paclitaxel	36314	0.122933435
ACH-000946	HEC265_ENDOMETRIUM	SNS-314	24995524	0.122947404
ACH-000781	NCIH2023_LUNG	paclitaxel	36314	0.122980538
ACH-000468	PK45H_PANCREAS	combretastatin-A-4	5351344	0.123028771
ACH-000359	MG63_BONE	altretamine	2123	0.123044288
ACH-001075	NCIH292_LUNG	AS-703026	44187362	0.123046619
ACH-000527	OVISE_OVARY	piperazine	4837	0.12305097
ACH-000482	RERFLCKJ_LUNG	navitoclax	24978538	0.123139546
ACH-000927	BT474_BREAST	colchicine	6167	0.1232354
ACH-000414	NCIH1944_LUNG	temoporfin	60751	0.123274985
ACH-000082	G292CLONEA141B1_BONE	elesclomol	300471	0.123376606
ACH-000810	SKMEL30_SKIN	GSK461364	15983966	0.123434654
ACH-000480	HUH7_LIVER	tosedostat	15547703	0.123490819
ACH-000965	RL952_ENDOMETRIUM	MLN0128	45375953	0.12366102
ACH-000163	SW579_THYROID	altretamine	2123	0.123712766
ACH-000609	SF126_CENTRAL_NERVOUS_SYSTEM	idasanutlin	53358942	0.123817375
ACH-000086	ACCMESO1_PLEURA	rigosertib	6918736	0.123913854
ACH-000169	RD_SOFT_TISSUE	idasanutlin	53358942	0.123918246
ACH-000027	GOS3_CENTRAL_NERVOUS_SYSTEM	tanespimycin	6505803	0.123951972
ACH-000929	NCIH2110_LUNG	EVP4593	509554	0.124111943
ACH-000652	SUIT2_PANCREAS	combretastatin-A-4	5351344	0.124134631
ACH-000630	YD8_UPPER_AERODIGESTIVE_TRACT	paclitaxel	36314	0.124206661
ACH-000274	HS852T_SKIN	vindesine	40839	0.12425679
ACH-000800	NCIH446_LUNG	tanespimycin	6505803	0.124258528
ACH-000582	COLO741_SKIN	EVP4593	509554	0.124317607
ACH-000774	RERFLCAD2_LUNG	elesclomol	300471	0.124365153
ACH-000936	EFO27_OVARY	CYT-997	11351021	0.124416852
ACH-000082	G292CLONEA141B1_BONE	pralatrexate	148121	0.124516146
ACH-000504	SNB75_CENTRAL_NERVOUS_SYSTEM	tanespimycin	6505803	0.124622336

ACH-000614	RVH421_SKIN	piperazine	4837	0.124629367
ACH-000759	MDAMB175VII_BREAST	cabazitaxel	129009963	0.124635606
ACH-000863	DBTRG05MG_CENTRAL_NERVOUS_SYSTEM	piperazine	4837	0.124672996
ACH-000721	HMC18_BREAST	dasatinib	3062316	0.124690915
ACH-000318	TE10_OESOPHAGUS	MLN0128	45375953	0.124707672
ACH-000232	U251MG_CENTRAL_NERVOUS_SYSTEM	everolimus	6442177	0.124794812
ACH-000261	RERFLCAI_LUNG	dasatinib	3062316	0.124904478
ACH-000701	RMUGS_OVARY	docetaxel	148124	0.124944187
ACH-000862	KMBC2_URINARY_TRACT	piperazine	4837	0.125024458
ACH-000685	L33_PANCREAS	7-aminocephalosporanic-acid	441328	0.125025165
ACH-000562	HCC78_LUNG	barasertib-HQPA	16007391	0.125030765
ACH-000189	RCC10RGB_KIDNEY	tanespimycin	6505803	0.125044698
ACH-000878	HCC15_LUNG	barasertib-HQPA	16007391	0.125068755
ACH-000364	U2OS_BONE	litronesib	25167017	0.125072165
ACH-000667	HCC44_LUNG	temsirolimus	129009966	0.125133145
ACH-000019	MCF7_BREAST	litronesib	25167017	0.12528133
ACH-000684	KMRC1_KIDNEY	NVP-BEZ235	11977753	0.125314246
ACH-000456	BCPAP_THYROID	idasanutlin	53358942	0.125324438
ACH-000457	CAL54_KIDNEY	filanesib	44224257	0.125345466
ACH-000788	A2058_SKIN	piperazine	4837	0.125350529
ACH-000450	MELHO_SKIN	elesclomol	300471	0.125366664
ACH-000532	SNU61_LARGE_INTESTINE	rigosertib	6918736	0.125378595
ACH-000950	LOVO_LARGE_INTESTINE	SNS-314	24995524	0.125398499
ACH-000102	GMS10_CENTRAL_NERVOUS_SYSTEM	AZD8330	16666708	0.125400361
ACH-000719	RMGI_OVARY	elesclomol	300471	0.125540566
ACH-000991	SNU81_LARGE_INTESTINE	elesclomol	300471	0.125547935
ACH-000090	PC3_PROSTATE	temoporfin	60751	0.125609917
ACH-000684	KMRC1_KIDNEY	tanespimycin	6505803	0.125792429
ACH-000759	MDAMB175VII_BREAST	paclitaxel	36314	0.125823877
ACH-000716	TT2609C02_THYROID	fluocinolone-acetonide	6215	0.125869506
ACH-000066	HCC4006_LUNG	litronesib	25167017	0.125881514
ACH-000155	SW1990_PANCREAS	litronesib	25167017	0.125962417
ACH-000860	NCIH358_LUNG	temsirolimus	129009966	0.126010502
ACH-000808	HUH28_BILIARY_TRACT	CYT-997	11351021	0.126189868
ACH-000976	HUCCT1_BILIARY_TRACT	idasanutlin	53358942	0.126216367
ACH-000651	SW620_LARGE_INTESTINE	SNS-314	24995524	0.126238804
ACH-000054	HT1080_SOFT_TISSUE	temoporfin	60751	0.126259935
ACH-000837	NCIH322_LUNG	elesclomol	300471	0.126267654
ACH-000212	CAL120_BREAST	tanespimycin	6505803	0.126291192
ACH-000468	PK45H_PANCREAS	tanespimycin	6505803	0.126407935
ACH-000723	YD10B_UPPER_AERODIGESTIVE_TRACT	idasanutlin	53358942	0.126415593
ACH-000174	CAL62_THYROID	NVP-BEZ235	11977753	0.126445289
ACH-000759	MDAMB175VII_BREAST	danusertib	11442891	0.126480051

ACH-000862	KMBC2_URINARY_TRACT	famciclovir	3324	0.126606021
ACH-000351	MKN1_STOMACH	temoporfin	60751	0.126657547
ACH-000456	BCPAP_THYROID	talazoparib	135565082	0.126693861
ACH-000376	SF295_CENTRAL_NERVOUS_SYSTEM	ingenol-mebutate	6918670	0.126711245
ACH-000652	SUIT2_PANCREAS	tanespimycin	6505803	0.126718802
ACH-000826	CAL12T_LUNG	pralatrexate	148121	0.126789363
ACH-000810	SKMEL30_SKIN	ingenol-mebutate	6918670	0.126852237
ACH-000946	HEC265_ENDOMETRIUM	ingenol-mebutate	6918670	0.126858208
ACH-000868	HCC1195_LUNG	ispinesib	6851740	0.126864269
ACH-000587	NCIH1975_LUNG	alvocidib	5287969	0.126919052
ACH-000191	BHT101_THYROID	tanespimycin	6505803	0.126976711
ACH-000595	LN229_CENTRAL_NERVOUS_SYSTEM	pralatrexate	148121	0.127046567
ACH-000260	SKNAS_AUTONOMIC_GANGLIA	idasanutlin	53358942	0.127128178
ACH-000408	TE5_OESOPHAGUS	irinotecan	60838	0.127146791
ACH-000759	MDAMB175VII_BREAST	MPI-0479605	46909588	0.127164167
ACH-000359	MG63_BONE	ispinesib	6851740	0.127190661
ACH-000890	SW1271_LUNG	NVP-BEZ235	11977753	0.127203065
ACH-000774	RERFLCAD2_LUNG	filanesib	44224257	0.127214681
ACH-000759	MDAMB175VII_BREAST	KX2-391	23635314	0.127294299
ACH-000042	PANC0203_PANCREAS	idasanutlin	53358942	0.127372757
ACH-000945	NCIH650_LUNG	tanespimycin	6505803	0.127399414
ACH-000967	SNUC2A_LARGE_INTESTINE	litronesib	25167017	0.127441558
ACH-000651	SW620_LARGE_INTESTINE	idasanutlin	53358942	0.127473729
ACH-000090	PC3_PROSTATE	litronesib	25167017	0.127633482
ACH-000163	SW579_THYROID	birinapant	49836020	0.127673255
ACH-000991	SNU81_LARGE_INTESTINE	everolimus	6442177	0.127811903
ACH-000495	TUHR4TKB_KIDNEY	paclitaxel	36314	0.127812285
ACH-000407	SNU685_ENDOMETRIUM	batimastat	5362422	0.127842992
ACH-000312	SKNBE2_AUTONOMIC_GANGLIA	piperazine	4837	0.127884971
ACH-000827	WM793_SKIN	EVP4593	509554	0.127904087
ACH-000730	SKMEL5_SKIN	ouabain	11527152	0.12803238
ACH-001239	WM2664_SKIN	KX2-391	23635314	0.128039635
ACH-000132	JHOS2_OVARY	tanespimycin	6505803	0.128046275
ACH-000549	SNU1076_UPPER_AERODIGESTIVE_TRACT	elesclomol	300471	0.128162666
ACH-000147	T47D_BREAST	barasertib-HQPA	16007391	0.128179204
ACH-000688	OV7_OVARY	GSK461364	15983966	0.128180413
ACH-000532	SNU61_LARGE_INTESTINE	AT13387	11955716	0.128246153
ACH-000968	COLO792_SKIN	temsirolimus	129009966	0.128258267
ACH-000630	YD8_UPPER_AERODIGESTIVE_TRACT	vinblastine	13342	0.128268064
ACH-000685	L33_PANCREAS	altretamine	2123	0.128301775
ACH-000274	HS852T_SKIN	colchicine	6167	0.128316798
ACH-000231	KALS1_CENTRAL_NERVOUS_SYSTEM	piperazine	4837	0.128400194
ACH-000903	FTC133_THYROID	CYT-997	11351021	0.12851218

ACH-000178	HS766T_PANCREAS	docetaxel	148124	0.128637697
ACH-000505	RKN_SOFT_TISSUE	trametinib	11707110	0.128749121
ACH-000107	CAPAN2_PANCREAS	GSK461364	15983966	0.128752703
ACH-000096	G401_SOFT_TISSUE	dasatinib	3062316	0.128773112
ACH-000376	SF295_CENTRAL_NERVOUS_SYSTEM	paclitaxel	36314	0.128789951
ACH-000842	SW480_LARGE_INTESTINE	litronesib	25167017	0.128814304
ACH-000138	CFPAC1_PANCREAS	litronesib	25167017	0.128828263
ACH-000403	NCIH747_LARGE_INTESTINE	paclitaxel	36314	0.128940278
ACH-000549	SNU1076_UPPER_AERODIGESTIVE_TRACT	mubritinib	6444692	0.12895797
ACH-000505	RKN_SOFT_TISSUE	SNS-314	24995524	0.12896699
ACH-000613	HOS_BONE	ingenol-mebutate	6918670	0.129133788
ACH-000035	NCIH1650_LUNG	ispinesib	6851740	0.129241163
ACH-000862	KMBC2_URINARY_TRACT	altretamine	2123	0.129385662
ACH-000713	CAOV3_OVARY	elesclomol	300471	0.129421588
ACH-000469	YH13_CENTRAL_NERVOUS_SYSTEM	tanespimycin	6505803	0.129454116
ACH-000099	SIMA_AUTONOMIC_GANGLIA	elesclomol	300471	0.129497154
ACH-000142	CAL29_URINARY_TRACT	altretamine	2123	0.129498497
ACH-000273	SF539_CENTRAL_NERVOUS_SYSTEM	temoporfin	60751	0.129499718
ACH-000566	SW1710_URINARY_TRACT	temsirolimus	129009966	0.12951648
ACH-000657	A2780_OVARY	7-aminocephalosporanic-acid	441328	0.129531584
ACH-000776	ONS76_CENTRAL_NERVOUS_SYSTEM	temsirolimus	129009966	0.129554672
ACH-000309	SKLU1_LUNG	7-aminocephalosporanic-acid	441328	0.12976029
ACH-000450	MELHO_SKIN	everolimus	6442177	0.129808295
ACH-000696	OVCAR8_OVARY	temsirolimus	129009966	0.129829146
ACH-000895	CL34_LARGE_INTESTINE	tanespimycin	6505803	0.129884219
ACH-000237	JHOM1_OVARY	piperazine	4837	0.12993955
ACH-000461	SNU1196_BILIARY_TRACT	ispinesib	6851740	0.12995116
ACH-000563	EBC1_LUNG	idasanutlin	53358942	0.129975875
ACH-000903	FTC133_THYROID	BNC105	24786555	0.13002678
ACH-000332	YAPC_PANCREAS	dasatinib	3062316	0.130064683
ACH-000677	SW1573_LUNG	elesclomol	300471	0.130347828
ACH-000813	T3M10_LUNG	everolimus	6442177	0.130418216
ACH-000188	SCC25_UPPER_AERODIGESTIVE_TRACT	temoporfin	60751	0.130485894
ACH-000661	WM1799_SKIN	trametinib	11707110	0.130639812
ACH-000680	SW948_LARGE_INTESTINE	BNC105	24786555	0.130671013
ACH-000771	BICR56_UPPER_AERODIGESTIVE_TRACT	KX2-391	23635314	0.130758991
ACH-001075	NCIH292_LUNG	ispinesib	6851740	0.130793089
ACH-000921	NCIH1339_LUNG	NVP-BEZ235	11977753	0.130807088
ACH-000893	NCIH1651_LUNG	temsirolimus	129009966	0.13086309
ACH-000946	HEC265_ENDOMETRIUM	talazoparib	135565082	0.130925741
ACH-000627	LCLC103H_LUNG	temoporfin	60751	0.130972809
ACH-000351	MKN1_STOMACH	MLN0128	45375953	0.131092467
ACH-000142	CAL29_URINARY_TRACT	piperazine	4837	0.131187564

ACH-000320	PSN1_PANCREAS	NVP-BEZ235	11977753	0.13125957
ACH-000139	PANC0327_PANCREAS	idasanutlin	53358942	0.131267482
ACH-000535	BXPC3_PANCREAS	elesclomol	300471	0.131281408
ACH-000730	SKMEL5_SKIN	AT13387	11955716	0.131312608
ACH-000945	NCIH650_LUNG	rigosertib	6918736	0.131381432
ACH-000457	CAL54_KIDNEY	KX2-391	23635314	0.131570944
ACH-000882	IGR1_SKIN	ingenol-mebutate	6918670	0.131646255
ACH-000479	KNS81_CENTRAL_NERVOUS_SYSTEM	tanespimycin	6505803	0.131727488
ACH-000117	EFM192A_BREAST	GSK461364	15983966	0.131749787
ACH-000771	BICR56_UPPER_AERODIGESTIVE_TRACT	vincristine	5388993	0.131753855
ACH-000906	ES2_OVARY	7-aminocephalosporanic-acid	441328	0.131765462
ACH-000164	PANC1_PANCREAS	BNC105	24786555	0.131792262
ACH-000368	SNU1105_CENTRAL_NERVOUS_SYSTEM	litronesib	25167017	0.131815462
ACH-000625	HEP3B217_LIVER	verubulin	11414799	0.131908969
ACH-000231	KALS1_CENTRAL_NERVOUS_SYSTEM	MLN0128	45375953	0.131966966
ACH-000368	SNU1105_CENTRAL_NERVOUS_SYSTEM	irinotecan	60838	0.131973873
ACH-000176	LOUNH91_LUNG	combretastatin-A-4	5351344	0.131996494
ACH-000674	NUGC4_STOMACH	elesclomol	300471	0.13201834
ACH-000035	NCIH1650_LUNG	BAY-87-2243	67377767	0.132033207
ACH-000965	RL952_ENDOMETRIUM	famciclovir	3324	0.132082551
ACH-000835	GCT_SOFT_TISSUE	tanespimycin	6505803	0.132087762
ACH-000684	KMRC1_KIDNEY	docetaxel	148124	0.132089287
ACH-000643	HDQP1_BREAST	rigosertib	6918736	0.132118312

Table S14. The drug target gene list. Based on the list of drugs obtained from the top 1% of predicted drug-cell line pairs, we found the target genes for these drugs from the PRISM database.

Drug name	Target gene ID
colchicine	GLRA1, GLRA2, TUBA1A, TUBA1B, TUBA1C, TUBA3C, TUBA3D, TUBA3E, TUBA4A, TUBB, TUBB1, TUBB2A, TUBB2B, TUBB3, TUBB4A, TUBB4B, TUBB6, TUBB8
docetaxel	BCL2, MAP2, MAP4, MAPT, NR1I2, TUBB, TUBB1
filanesib	KIF11
JNJ-26481585	HDAC1, HDAC10, HDAC11, HDAC2, HDAC3, HDAC4, HDAC5, HDAC6, HDAC7, HDAC8, HDAC9, MDM2
alvespimycin	HSP90AA1
NVP-AUY922	HSP90AA1, HSP90AB1
NSC-319726	NA
ganetespib	HSP90AA1
paclitaxel	BCL2, MAP2, MAP4, MAPT, NR1I2, TLR4, TUBB, TUBB1

LY2606368	CHEK1
cabazitaxel	TUBA4A, TUBB, TUBB1
FK-866	NAMPT
ispinesib	KIF11
delanzomib	CMA1, CTSG, CYP3A4, ELANE
BNC105	NA
BI-2536	BRD4, PLK1, PLK2, PLK3
bortezomib	PSMA1, PSMA2, PSMA3, PSMA4, PSMA5, PSMA6, PSMA7, PSMA8, PSMB1, PSMB10, PSMB11, PSMB2, PSMB3, PSMB4, PSMB5, PSMB6, PSMB7, PSMB8, PSMB9, PSMD1, PSMD2, RELA
taltobulin	NA
MPI-0479605	TTK
pralatrexate	DHFR, TYMS
AZD8330	NA
piperazine	GABRB3
vindesine	TUBB, TUBB1
dinaciclib	CDK1, CDK2, CDK5, CDK9
epothilone-b	TUBA1A, TUBA1B, TUBA1C, TUBA3C, TUBA4A, TUBA8, TUBB, TUBB1, TUBB3, TUBB4A, TUBB4B
vincristine	TUBA4A, TUBB
selinexor	XPO1
combretastatin-A-4	NA
temsirolimus	MTOR
vinblastine	JUN, TUBA1A, TUBB, TUBD1, TUBE1, TUBG1
panobinostat	HDAC1, HDAC2, HDAC3, HDAC4, HDAC6, HDAC7, HDAC8, HDAC9
tanespimycin	HSP90AA1
elesclomol	HSPA1A
crystal-violet	NA
rigosertib	PLK1
BAY-87-2243	HIF1A
litronesib	KIF11
everolimus	MTOR
floxuridine	TYMS
trametinib	MAP2K1, MAP2K2
temoporfin	NA
ingenol-mebutate	PRKCA, PRKCB, PRKCD, PRKCE, PRKCG
raltitrexed	FPGS, TYMS
carfilzomib	PSMA1, PSMA2, PSMA3, PSMA4, PSMA5, PSMA6, PSMA7, PSMA8, PSMB1, PSMB10, PSMB11, PSMB2, PSMB3, PSMB4, PSMB5, PSMB6, PSMB7, PSMB8, PSMB9

tosedostat	ANPEP, LAP3, NPEPPS
danusertib	AURKA, AURKB, AURKC, FGFR1, NTRK1, RET, SLK
poziotinib	EGFR, ERBB2, ERBB4
vinflunine	NA
GSK461364	PLK1
Ro-4987655	MAP2K1
7-aminocephalosporanic-acid	NA
dasatinib	ABL1, ABL2, BLK, EPHA2, FGR, FRK, FYN, HCK, KIT, LCK, LYN, PDGFRB, SRC, SRMS, STAT5B, YES1
altretamine	NA
NVP-BEZ235	ATR, MTOR, PIK3CA, PIK3CD, PIK3CG
KX2-391	SRC
mubritinib	EGFR, ERBB2
cephalomannine	NA
irinotecan	TOP1, TOP1MT
SNS-314	AURKA, AURKB, AURKC
idasanutlin	MDM2, TP53
plinabulin	NA
alvocidib	CDK1, CDK2, CDK4, CDK5, CDK6, CDK7, CDK8, CDK9, EGFR, PYGM
MLN0128	MTOR, PIK3CA, PIK3CD, PIK3CG
PD-0325901	MAP2K1
PF-477736	CHEK1, CHEK2
AS-703026	MAP2K1, MAP2K2
talazoparib	PARP2
volasertib	PLK1
ouabain	ATP1A1
fluocinolone-acetonide	NR3C1, SERPINA6
CYT-997	TUBB
barasertib-HQPA	AURKB
D-64131	TUBB
TAK-733	MAP2K1
verubulin	TUBB
desonide	NR3C1, PLA2G1B
napabucasin	STAT3
BMS-626529	NA
12-O-tetradecanoylphorbol-13-acetate	KCNT2, TRPV4
AZD8931	EGFR, ERBB2, ERBB3
cobimetinib	NA
dabrafenib	BRAF, LIMK1, NEK11, RAF1, SIK1
tipifarnib	FNTA, FNTB
birinapant	BIRC2, XIAP
pelitinib	EGFR

AT13387	HSP90AA1
temazepam	GABRA1, GABRA2, GABRA3, GABRA4, GABRA5, GABRA6, GABRB1, GABRB2, GABRB3, GABRD, GABRE, GABRG1, GABRG2, GABRG3, GABRP, GABRQ, GABRR1, GABRR2, GABRR3, TSPO
navitoclax	BCL2, BCL2L1, BCL2L2
EVP4593	NA
famciclovir	NA
batimastat	ADAM28, ADAMTS5, MMP12, MMP16, MMP2, MMP8
